# Treatment With Lipopolysaccharide Induces Distinct Changes in Metabolite Profile and Body Weight in 129Sv and Bl6 Mouse Strains

**DOI:** 10.3389/fphar.2020.00371

**Published:** 2020-03-27

**Authors:** Maria Piirsalu, Egon Taalberg, Kersti Lilleväli, Li Tian, Mihkel Zilmer, Eero Vasar

**Affiliations:** ^1^Department of Physiology, Institute of Biomedicine and Translational Medicine, University of Tartu, Tartu, Estonia; ^2^Center of Excellence for Genomics and Translational Medicine, University of Tartu, Tartu, Estonia; ^3^Department of Biochemistry, Institute of Biomedicine and Translational Medicine, University of Tartu, Tartu, Estonia

**Keywords:** lipopolysaccharide, 129Sv strain, Bl6 strain, metabolic profiling, inflammation, innate immunity, hypometabolism

## Abstract

Mouse strains differ significantly in their behaviors and responses to pathogenic and pharmacological agents. This study seeks to characterize behavioral and metabolomic profiles of two widely used mouse lines, 129S6/SvEvTac (129Sv) and C57BL/6NTac (Bl6), to acute administration of lipopolysaccharide (LPS). LPS caused a significant suppression of locomotor activity and a decline in body weight (BW) in both strains within 24 h. However, the BW loss was more pronounced in Bl6 than in 129Sv. Comparison of strains revealed clear differences between their metabolomic profiles. According to the general linear model analysis (GLM), the 1.5 h LPS challenge in Bl6 caused a decrease of propionylcarnitine (C3), glucogenic amino acids, and acetylornithine (Ac-Orn), whereas the response of 129Sv included decreased concentrations of short-chain acylcarnitines (SCACs), citrulline, and elevation of glycerophospholipid (PCaa C42:0) and sphingolipid [SM(OH)C16:1]. 24 h after LPS administration, robust alterations in lipid profile were observed in both strains. LPS treatment caused elevation of sphingolipids, phosphatidylcholine diacyls (PCaa) as well as a decrease in lysophosphatidylcholines (LysoPC). However, the number of elevated PCaa and sphingolipids was considerably higher in 129Sv. In addition to lipids, 24 h LPS challenge in Bl6 mice induced increased levels of kynurenine (KYN), putrescine and decreased levels of citrulline, hexoses, Ac-Orn, and PC acyl-alkyl (PCae 38:2) as well as severe BW loss. In contrast, the 24 h LPS challenge in 129Sv mice induced increased levels of KYN, long-chain acylcarnitines (LCACs) and decreased levels of citrulline as well as moderate BW loss. Altogether, our study revealed both similarities and differences in response to LPS in Bl6 and 129Sv strains. For major differences, Bl6 mice showed stronger reduction of BW 24 h after LPS treatment, accompanied by significantly reduced levels of hexoses, the ratio between LysoPC16:1/LysoPC16:0, and elevated levels of neuroprotective putrescine. In 129Sv mice, the BW loss was milder, accompanied by increased levels of hydroxylated LCACs, probably reflecting shifts in oxidative metabolism of fatty acids. One may suggest that LPS caused stronger hypometabolic state in the Bl6 mice than in the 129Sv strain. Altogether, this study confirms that Bl6 and 129Sv mice display vastly distinct adaptation capacities independent from the nature of stressful challenge.

## Introduction

Majority of studies with transgenic mouse models involve the use of inbred mouse strains, two of the most common being C57BL/6NTac (Bl6) and 129S6/SvEvTac (129Sv). However, these two mouse strains differ significantly in several ways, including their responses to pharmacological and pathogenic agents. Bl6 mice actively cope in stressful situations, whereas the coping strategy of the 129Sv line is inherently passive. Prior environmental enrichment in home cages amplifies the exploratory activity of Bl6 mice in a novel and stressful environment (Abramov et al., 2008), whereas in similar conditions, 129Sv mice display increased anxiety and loss in BW (Abramov et al., 2008; Heinla et al., 2014). Bl6 line demonstrates increased aggressiveness, extensive barbering behavior, and significant preference for alcohol (Belknap et al., 1993; Middaugh et al., 1999; Sarna et al., 2000; Heinla et al., 2014). In contrast, pain sensitivity of the 129Sv strain is significantly reduced compared to the Bl6 mice (Võikar et al., 2004; Abramov et al., 2008). While cat odor elicits anxiety responses from Bl6 mice, it does not stimulate such responses from the 129Sv strain (Raud et al., 2007). Acute treatment with amphetamine elicits different responses in these mouse strains, as well (Chen et al., 2007; Vanaveski et al., 2018), and recent evidence links these differences to a frameshift mutation in the 129Sv Disc1 gene (Mukaida et al., 1996), which causes alterations in dopamine homeostasis and affects cognitive abilities (Koike et al., 2006; Trossbach et al., 2016).

We have recently found that 129Sv and Bl6 strains also differ in the metabolite signatures in their blood samples (Narvik et al., 2018). Acylcarnitine C5- is dominating in 129Sv mice, whereas the metabolite signature of Bl6 contains three biogenic amines: Ac-Orn, alpha-aminoadipic acid (alpha-AAA), carnosine, and LysoPC16:1. Moreover, the levels of hexoses tended to be higher in Bl6 mice compared to 129Sv, possibly allowing higher glucose availability for emerging metabolic needs (Narvik et al., 2018). These metabolic differences could be associated with the domination of active coping behavioral strategies in the Bl6 strain, requiring targeted metabolic expenses.

Responses of 129Sv and Bl6 to pathogens also differ. For example, infecting 129Sv mice with influenza virus causes strong inflammatory cytokine release, and the animals are more prone to die from infection (Davidson et al., 2014). In contrast, the inflammatory cytokine response in the Bl6 strain is modest, and the animals effectively cope with the infection (Davidson et al., 2014).

Immunological and behavioral responses to LPS in different inbred mouse strains have been investigated in several studies, which have shown differences between mouse strains (Mahieu et al., 2006; Meneses et al., 2018). Drastic changes in urinary metabolites in mice exposed to LPS have also been shown (Laiakis et al., 2012). Although rather widely studied, the effect of systemic inflammation on blood plasma metabolites in commonly used inbred mouse strains is poorly understood. Thus, we undertook an extensive metabolome characterization of the response to LPS in two commonly used inbred mouse strains.

In order to explore the possible differences in coping with inflammatory influences, we administered LPS, the activator of innate immune response, into mice of the two above-described strains. The effect of LPS (0.5 mg/kg) was studied on locomotor activity, body temperature, and BW. In addition, blood samples were taken 1.5 and 24 h after the treatment with LPS in order to measure the profile of blood metabolites. The obtained data enhance the knowledge pool of distinct coping strategies to stressful events in 129Sv and Bl6 mouse strains. Bl6 displays a significant hypometabolic response with reduced levels of hexoses accompanied by robust BW decline 24 h after the injection of LPS. In 129Sv mice, reduction of hexoses did not occur, and the LPS challenge was accompanied by less severe BW loss and body temperature reduction. These different metabolic responses in 129Sv and Bl6 mice may explain their distinct response to infections, like influenza. Altogether, this study confirms that Bl6 and 129Sv mice display vastly distinct adaptation capacities independent from the nature of stressful challenge.

## Materials and Methods

### Animals

Wild-type male mice (16–23 week-old) from the two inbred strains, 129S6/SvEvTac (129Sv; n = 56) and C57BL/6NTac (Bl6, n = 56), were bred and housed in the Laboratory Animal Centre at University of Tartu. Mice were kept under standard conditions with unlimited access to food and water on a 12/12 h light/dark cycle (lights on from 07:00 to 19:00 h).

### LPS Treatment

LPS (derived from *E. coli* serotype 0111:B4; Sigma–Aldrich, St. Louis, MO, USA) was dissolved in 0.9% NaCl (saline). Injections were administered intraperitoneally (i.p.) at a dose of 0.5 mg/kg. The vehicle consisted of 0.9% NaCl in an equivalent volume.

**Figure 1:**
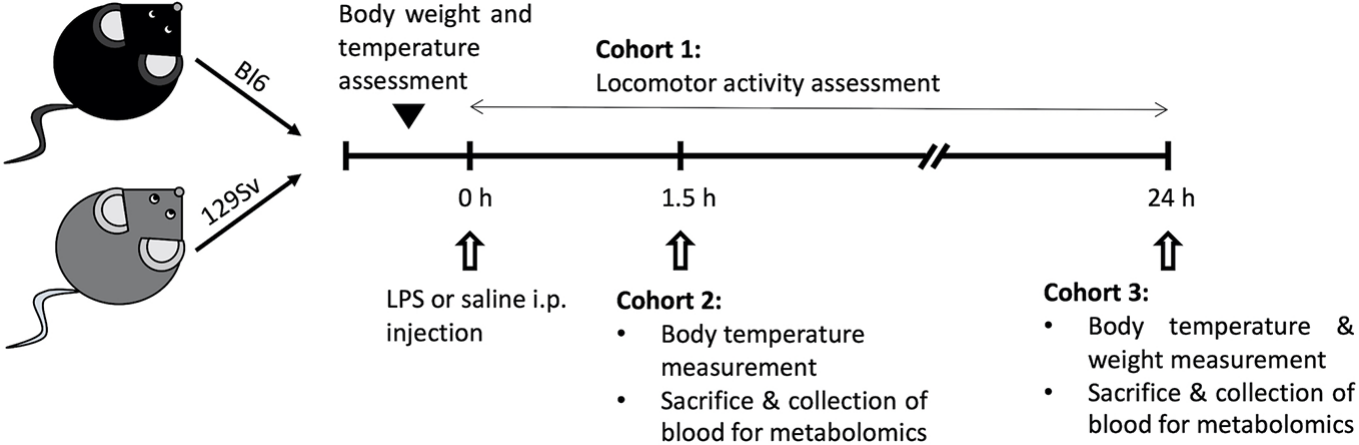
Schematic overview of the experimental design. Male mice on a Bl6 (n = 56) and 129Sv (n = 56) background were used in this study. Mice from both strains were randomly assigned to three different experimental groups: cohort 1 was used to determine the effect of LPS on locomotor activity (Bl6 n = 16; 129Sv n = 16); cohort 2 was used to study the effect of LPS on the profile of blood metabolites after 1.5 h treatment (Bl6 n = 20; 129Sv n = 20); cohort 3 was used to study the effect of LPS on the profile of blood metabolites after 24 h treatment (Bl6 n = 20; 129Sv n = 20). In each cohort, both strains were further divided into two groups: LPS administration group and control group (saline administration).

Mice were randomly divided into three cohorts (**Figure 1**): 1) 1.5 h LPS challenge cohort, containing mice sacrificed and trunk blood collected 1.5 h post-LPS or saline treatment (Bl6 saline, n = 10; Bl6 LPS, n = 10; 129Sv saline, n = 10; 129Sv LPS, n = 10); 2) 24 h LPS challenge cohort, containing mice sacrificed and trunk blood collected 24 h post LPS or saline treatment (Bl6 saline, n = 10; Bl6 LPS, n = 10; 129Sv saline, n = 10; 129Sv LPS, n = 10); cohorts 1 and 2 were used for metabolite measurements and placed back to their home cages after LPS i.p. injection; 3) locomotor activity response group (Bl6 saline, n = 8; Bl6 LPS, n = 8; 129Sv saline, n = 8; 129Sv LPS, n = 8). Locomotor activity was registered during 24 h period after LPS administration.

### Body Weight and Rectal Temperature Determination

Changes in body temperature were evaluated at 0 h, 1.5 h, and 24 h post-LPS and saline i.p. injection. Body weight was measured before injection and 24 h post injection. Body temperature was measured using a rectal thermometer (TSE Technical & Scientific Equipment GmbH, Germany) by inserting a lubricated rectal probe 2 cm into the rectum and maintained until stable readings could be obtained.

### Locomotor Activity

The effect of LPS on locomotor activity, reaction to novel environment, and anxiety-like behavior was monitored in PhenoTyper^®^ (EthoVision 3.0, Noldus Information Technology, Wageningen, The Netherlands). The Phenotyper testing consisted of 24 h trial where animals were individually housed in 30 cm × 30 cm × 35 cm plexiglass cages with sawdust bedding, similar to a home cage. Each animal had an individual cage. Mice had free access to food and water throughout the test. Mice were kept under a 12:12 h light/dark cycle (lights off at 19:00 h), similar to animal room standard conditions. Each cage was equipped with a top unit with integrated infrared sensitive camera and infrared LED lights, which makes tracking possible in the dark phase. Open field arena was virtually divided into two zones, central and peripheral zones. The center zone was defined as half of the overall area of the test arena. Total distance travelled in the whole arena and central zone of the arena (cm) was measured. Animal movements were continuously recorded by video-tracking system.

### Sample Collection

Mice were euthanized by decapitation, and trunk blood was collected into EDTA-coated microcentrifuge tubes and stored on ice. All the tubes were centrifuged at 2,000 g for 15 min at 4°C. Plasma supernatant was separated and stored at −80°C until further analysis.

### Measurement of Metabolites

AbsoluteIDQ™ p180 kit (BIOCRATES Life Sciences AG, Innsbruck, Austria) was used to determine plasma levels of 186 different compounds. Samples were measured on mass-spectrometry QTRAP 4500 (Sciex, Framingham, MA, USA), in combination with high-performance liquid chromatography (HPLC) (Agilent 1260 series, Agilent Technologies, Waldbronn, Germany).

The first stage of the sample preparation was carried out on AbsoluteIDQ Kit plate that was included in the test kit which had 96 wells for accommodating zero-sample, three phosphate-buffered saline samples, seven calibration standards, and at least three quality controls. The plasma samples were thawed and centrifuged at 4°C for 5 min at 2,750 × g. In all the wells, except the well in A1 position, 10 μl of internal standard mix was added. After that 10  μl of calibration standards, phosphate-buffered saline, quality controls and plasma samples were transferred into their respective wells. In each well was added 50 µl of 5% solution of phenyl isothiocyanate in pyridine/ethanol/water (1:1:1, v/v/v) for amino acid derivatization.

After 20 min of incubation the plate was dried at room temperature under dry airflow, and all the compounds were extracted into the solution using 300 µl 5 mM ammonium acetate in methanol. After shaking for 30 min the plate was centrifuged which filtered the extract into the underlying 96-well capture plate through a filter membrane. From the capture plate 50 µl of the solution was transferred to another 96-well plate and diluted with 250 µl 40% (v/v) methanol in water for liquid chromatography (LC–MS) techniques. For flow injection analysis (FIA) 20 µl of the solution was transferred to another 96-well plate and diluted using 380 µl FIA mobile phase solvent which was prepared by diluting Biocrates Solvent I provided with the kit in 290 ml HPLC grade methanol.

Amino acids and biogenic amines in the samples were measured using the LC–MS techniques. Acylcarnitines (Cx:y), hexoses, sphingolipids [SMx:y or SM (OH)x:y], glycerophospholipids [lysophosphatidylcholines (lysoPCx:y), and phosphatidylcholines (PCaa x:y and PC ae x:y)] were measured using the FIA tandem mass spectrometry. For both modes of analyzing, Multiple Reaction Monitoring was used. Concentrations of the metabolites were calculated automatically by the MetIDQ™software (BIOCRATES Life Sciences AG) in μM.

### Statistical Analysis

Results are expressed as mean values ± SD. Statistical analyses for behavioral experiments, metabolomic data, body weight, and temperature were performed using GraphPad Prism 8 software. Shapiro–Wilk test was applied to assess the normality of data distribution. To normalize the distribution of locomotor and metabolomic data, logarithmic transformation (log_10_) of the values was preformed prior to data analysis. Body weight (ΔBW) and temperature (ΔT) changes were expressed as change of the initial weight and temperature (temperature post-administration − temperature pre-administration; weight post-administration − weight pre-administration). Comparison of body weight and temperature change between saline and LPS treatment groups was performed using two-way ANOVA (strain × treatment for ΔBW; time × treatment for ΔT), followed by Bonferroni *post hoc* test. To determine basal metabolite differences between Bl6 and 129Sv, saline treated control mice log_10_-data were compared using t-tests and Bonferroni correction (*p* value less or equal to 0.0002). Comparison of metabolomic data between groups was performed using two-way ANOVA (treatment × time) followed by Bonferroni *post hoc* test. All differences were considered statistically significant at *p* < 0.05. General linear model (GLM) multivariate analysis with a backward elimination procedure was performed to examine the associations between ΔBW, ΔT, metabolites, and their ratios in Bl6 and 129Sv mice. The associations between significantly altered metabolites, body weight, and temperature change 1.5 h and 24 h after LPS administration were analyzed using the Pearson correlation. Applying Cytoscape software and the “MetScape” plugin tool, the significant correlations (*p* > 0.05) were used to construct correlation networks.

### Ethics

All animal procedures were performed in accordance with the European Communities Directive (2010/63/EU) and permit (No. 141, April 17, 2019) from the Estonian National Board of Animal Experiments.

## Results

### LPS-Implicated Locomotor Activity, Body Temperature, and Weight Changes

**Figure 2:**
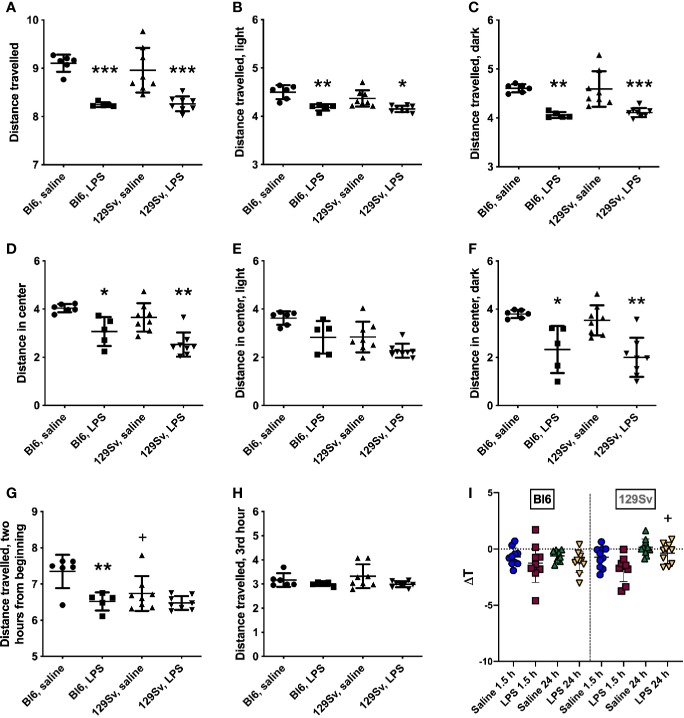
Locomotor activity (log_10_ values, data expressed as mean ± SD) of Bl6 and 129Sv mice in the 24 h LPS challenge. **(A)** Total distance travelled in 24 h period. **(B)** Total distance travelled in lights-on period. **(C)** Total distance travelled in lights-off period. Center distance travelled in 24-h cycle **(D)**, lights-on **(E)** and lights-off **(F)** periods. Distance travelled 2 h from the beginning **(G)** and on the third hour **(H)**. * significant difference between LPS and saline administration groups (Bonferroni’s multiple comparisons): *p ≤ 0.05, **p ≤ 0.01, ***p ≤ 0.001; ^+^significant difference between Bl6 and 129Sv mice in saline administration groups, ^+^*p* ≤ 0.05. **(I)** Body temperature (ΔT) change 1.5 and 24 h after 0.5 mg/kg LPS administration in Bl6 and 129Sv. Values are represented as mean ± SD. Two-way ANOVA followed by Bonferroni *post hoc* test: + a significant difference between LPS treatment groups: ^+^*p* < 0.05.

A single intraperitoneal injection of LPS (0.5 mg/kg) was administered to Bl6 and 129Sv mice, and locomotor activity was recorded for 24 h in Phenotyper cages. For the readings of locomotor activity, original values were converted to log_10_ values in order to make the data correspond to normal distribution. Results were analyzed using two-way ANOVA (strain × treatment) followed by Bonferroni *post hoc* test. Both strains exhibited significant LPS-induced suppression in their motor response in the 24-h period in the whole arena (treatment—*F*_(1,23)_ = 49.19, *p* < 0.0001; **Figures 2A–C**). Additionally, LPS-exposed mice travelled significantly shorter distance in the central zone (treatment—*F*_(1,23)_ = 28.30, *p* < 0.0001) compared to their control counterparts (**Figure 2D**). However, when dividing 24 h cycle into lights-on and lights-off periods, LPS-induced suppression of locomotor activity in the center of the arena was only significant during the dark period (treatment—*F*_(1,23)_ = 29.99, *p* < 0.0001; **Figures 2E, F**).

Difference in motor activity between Bl6 and 129Sv was observed within 2 h from the beginning of Phenotyper testing. The motor activity of the saline-treated 129Sv mice was significantly lower compared to that of the saline-treated Bl6 animals [strain—*F*_(1,23)_ = 4.96, *p* = 0.04; treatment—*F*_(1,23)_ = 13.87, *p* = 0.001; **Figure 2G**]. However, this difference was no longer evident from the third hour onward (**Figure 2H**). This observation could reflect higher anxiety-like trait of 129Sv in the beginning of behavioral testing indicating passive adaptation.

Change in body temperature (ΔT) of Bl6 and 129Sv mice was evaluated 1.5 and 24 h post saline and LPS administration. Body temperature measurements were conducted with mice housed in their home-cage environment. Groups were compared using two-way ANOVA (time × treatment) followed by Bonferroni *post hoc* test. Significant effect of time (*F*_(1,__35)_ = 13.01 *p* =0.00096) and treatment (*F*_(1,__35)_ = 6.39 *p* =0.016) was observed in 129Sv. Comparison of groups demonstrated that ΔT of LPS treated mice was slightly lower compared to that of saline treated controls in both strains, although no statistically significant difference was established between the LPS and saline groups. However, there was a significant difference between 1.5 h and 24 h LPS response in 129Sv. 24 h after LPS administration, the ΔT of 129Sv was significantly higher compared to 1.5 h LPS treatment, revealing different LPS-induced thermoregulation in 1.5 and 24 h time points. In contrast ΔT of Bl6 mice was similar 1.5 and 24 h after LPS administration (**Figure 2I**).

Two-way ANOVA (strain × treatment) demonstrated significant difference in 24 h BW change (ΔBW) between the LPS and saline treated mice in both strains. LPS administration induced a highly significant BW loss in both strains (Treatment: *F*_(1,36)_ = 175.8, *p* < 0.0001; Strain: *F*_(1,36)_ = 20.18, *p* < 0.0001; strain × treatment: *F*_(1,36)_ = 3.80, *p* = 0.06). However, LPS-induced BW loss was substantially more prominent in Bl6 as they lost 12.1 ± 0.68% of BW during the 24-h time, whereas in 129Sv the decline was merely 7.6 ± 0.51% of the initial BW (**Table 1**).

**Table 1:** Body weight change (□BW) and weight loss % change of the initial weight (weight2 − weight1/weight1 × 100%) 24 h after 0.5 mg/kg LPS administration.

	Saline Bl6 (Mean ± SD)	LPS Bl6 (Mean ± SD)	Saline 129Sv (Mean ± SD)	LPS 129Sv (Mean ± SD)	Two-way ANOVA *p*-value		
					Strain	Treatment	Strain × Treatment
24 h ΔBW (g)	−0.50 ± 0.73	−3.44 ± 0.73^****^	−0.01 ± 0.40	−2.2 ± 0.52^****+++^	< 0.0001	< 0.0001	0.06
24 h ΔBW (%)	−1.7 ± 2.49	−12.1 ± 2.16	−0.08 ± 1.43	−7.6 ± 1.62			

Values are represented as mean ± SD. Two-way ANOVA followed by Bonferroni *post hoc* test: * a significant difference between saline and LPS treatment: ****p < 0.0001; + a significant difference between Bl6 and 129Sv mice of the same treatment group: ^+++^p < 0.001.

### Metabolic Profile Differences of Bl6 and 129Sv

To determine the basal differences in metabolic profile between Bl6 and 129Sv, saline treated control animals were compared using paired *t*-tests and Bonferroni correction (*p* value less or equal to 0.0002) as well as with the general linear model (GLM) multivariate analysis.

Six metabolites survived the Bonferroni correction in both 1.5 and 24 h saline treatment groups. These metabolites included SCACs (C5-), biogenic amines Ac-Orn, alpha-AAA, carnosine, sphingolipid (SL) SM (OH) C22:2 and the ratio of C5-/carnitine (C5-/C0). Elevations in Bl6 included Ac-Orn, alpha-AAA and carnosine (**Supplementary Figures 1A–C**). In contrast, elevations in 129Sv included C5- and SM (OH) C22:2 (**Supplementary Figures 1E, F**). Additionally, 24 h saline treated Bl6 mice had higher blood levels of serotonin and lower C4- compared to 129Sv. This difference was missing in the 1.5 h saline treatment groups due to the higher values of serotonin and lower blood plasma levels of C4- in 129Sv, probably indicating acute stress-induced changes in 129Sv.

GLM analysis confirmed a significant main effect [1.5 h saline: *F*_(10,9)_ = 58.37, *p* < 0.0001; 24 h saline: *F*_(10,4)_ = 87.08, *p* = 0.0003] of mouse strain on the levels of various metabolites. In both the 1.5 and 24 h saline treatment groups significantly different metabolite levels between Bl6 and 129Sv included C5-, biogenic amines (Ac-Orn, alpha-AAA, carnosine), LysoPCa C16:1, and SM (OH) C22:2 (all *p* ≤ 0.0001). In addition, there was a significant difference in the blood levels of PC aa C34:3 (*p* = 0.001), PC ae C38:4 (*p* = 0.005), hexoses (*p* = 0.005), and LysoPC a C20:3 (*p* = 0.009) in the 1.5 h group and of PC ae C40:6 (*p* = 0.0003), PC ae C36:2 (*p* = 0.004), SM (OH) C14:1 (*p* = 0.004), and PC ae C38:2 (*p* = 0.008) in the 24 h group (**Supplementary Table 3**).

### Strain Specific Metabolic Shifts Induced by LPS

First, to identify differences between saline and LPS administration, Bl6 and 129Sv strains were analyzed separately using two-way ANOVA [treatment (saline or LPS) × time (1.5 h or 24 h LPS challenge)]. Bonferroni *post hoc* analysis was used when applicable after statistically significant ANOVA results.

#### LPS Induced Alterations of Metabolic Profile in Bl6

LPS administration induced altered levels of 87 metabolites and their ratios in the Bl6 strain. Twenty-one of them were altered early in the 1.5 h LPS challenge, 58 were altered later in the 24 h LPS challenge, and eight of them were altered in both time points. Detailed results are presented in **Supplementary Table 1**.

**Figure 3:**
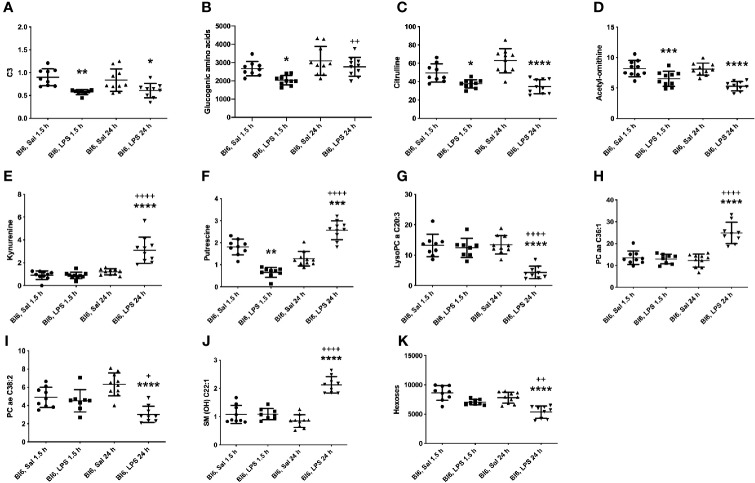
LPS treatment-induced changes in concentrations of **(A)** C3, **(B)** glucogenic amino acids, **(C)** citrulline, **(D)** Ac-Orn, **(E)** KYN, **(F)** putrescine, **(G)** lysophosphatidylcholines (C20:3), **(H)** phosphatidylcholine diacyls (C36:1), **(I)** phosphatidylcholine acyl-alkyls (C38:2), **(J)** sphingolipids (OH C22:1) and **(K)** hexoses in Bl6 mice after 1.5 and 24 h from administration. Data was analyzed by two-way ANOVA followed by the Bonferroni *post hoc* test. *significant *p* value between saline and LPS treatment; ^+^significant *p* value between 1.5 h and 24 h LPS treatments. *p ≤ 0.05, **p ≤ 0.01, ***p ≤ 0.001, ****p ≤ 0.0001, ++*p* ≤ 0.01, ++++*p* ≤ 0.0001.

Metabolites that were decreased 1.5 h as well as 24 h after LPS treatment are SCACs (C3, C4-), amino acids citrulline and tyrosine (Tyr), biogenic amines (Ac-Orn, putrescine), and the ratio between Tyr and phenylalanine (Phe). Putrescine was decreased 1.5 h after LPS treatment; however, 24 h after LPS treatment it was significantly increased compared to saline treated controls (**Figure 3F**). Accordingly, the ratio of spermidine/putrescine exhibited a significant increase 1.5 h after LPS treatment and a significant decrease 24 h after LPS treatment.

1.5 h LPS administration induced a significant reduction in ACs (C3:1, C4:1, C5-, C5:1, C6:1, C12-DC, C14:2-OH; **Figure 3A**), amino acids: alanine (Ala), glycine (Gly), histidine (His), Phe, methionine (Met), proline (Pro), serine (Ser), threonine (Thr), and valine (Val), biogenic amine alpha-AAA as well as in the sum of aromatic amino acids (AAA). Furthermore, the sum of glucogenic amino acids was significantly reduced (**Figure 3B**). In contrast, the ratio of Gly/Ser was elevated.

24 h LPS challenge induced significant alterations in several metabolite levels compared to saline treated animals, including decrease in serotonin, LysoPC acyls (LysoPC a C16:1, LysoPC a C17:0, LysoPC a C18:1, LysoPC a C18:2, LysoPC a C20:3, LysoPC a C20:4), PC acyl-alkyls (PC ae C36:2, PC ae C38:2, PC ae C38:3, PC ae C38:5), hexoses as well as in the ratio between Leu/KYN, serotonin/KYN, LysoPC a C16:1/LysoPC a C16:0 and LysoPC a C18:2/LysoPC a C18:1. On the other hand, significant elevation was observed of LCACs (C16, C16:1, C18, C18:1 and C18:2), biogenic amines [asymmetric dimethylarginine (ADMA), symmetric dimethylarginine (SDMA), KYN], PC diacyls (PC aa C32:3, PC aa C34:1, PC aa C34:2, PC aa C36:1, PC aa C38:4, PC aa C38:6, PC aa C40:5, PC aa C40:6, PC aa C42:4) and in the ratio of SCACs to free carnitine (CRT-1), LCACs to free carnitine (CPT-1), C5-/C0, Arg/citrulline, KYN/Trp, KYN/alpha-AAA and LysoPC a C20:4/LysoPC a C20:3. One of the most significant alterations was the elevation of 12 out of 15 circulating sphingolipids. Most significant results are highlighted in **Figure 3**.

#### LPS Induced Alterations of Metabolic Profile in 129Sv

The pattern of altered levels of metabolites in 129Sv differed from that of Bl6. 1.5 h LPS challenge caused less alterations in 129Sv than seen in Bl6. 1.5 h after LPS administration merely 9 altered levels of metabolites and their ratios were identified, including decrease in SCACs (C2, C3, C4-, C5-), citrulline, ratios of C3/C3, C5-/0, Tyr/Phe and increase in PC diacyl PC aa C42:0. Detailed results are presented in **Supplementary Table 2**.

**Figure 4:**
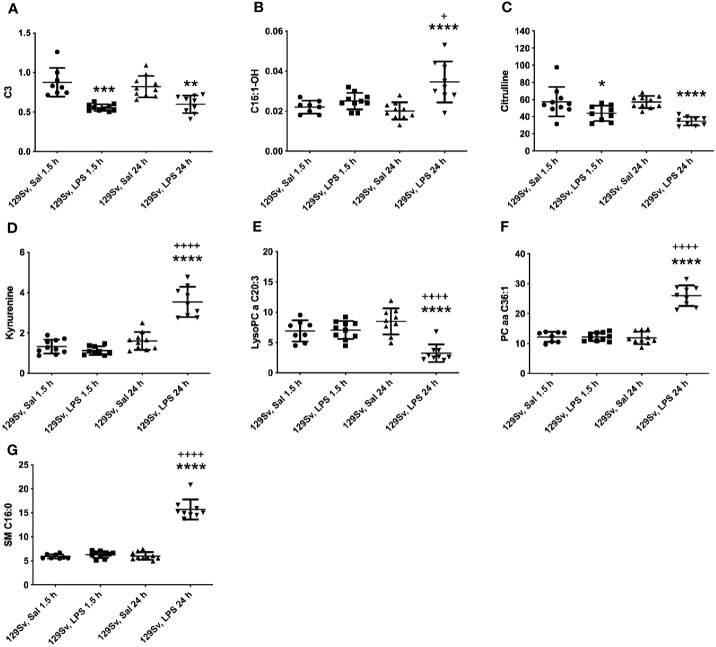
LPS treatment-induced changes in concentrations of **(A)** SCACs (C3), **(B)** hydroxylated LCACs (C16:1-OH), **(C)** citrulline, **(D)** KYN, **(E)** lysophosphatidylcholines (C20:3), **(F)** phosphatidylcholine diacyls (C36:1) and **(G)** sphingolipids (C16:0) in 129Sv mice 1.5 and 24 h after administration. Data was analyzed by two-way ANOVA followed by the Bonferroni *post hoc* test. *significant *p* value between saline and LPS treatment; ^+^significant *p* value between 1.5 h and 24 h LPS treatments. *p ≤ 0.05, **p ≤ 0.01, ***p ≤ 0.001, ****p ≤ 0.0001, ++++*p* ≤ 0.0001.

24 h after LPS administration large-scale alterations were observed in the metabolic profile, including 71 altered levels of metabolites and their ratios. Citrulline was the only amino acid significantly reduced in 129Sv. Accordingly, the ratio Arg/citrulline was elevated. Similarly to Bl6 mice, 129Sv exhibited significant decrease of SCAC (C3) and increase in LCACs (C12, C14:1, C14:1-OH, C14:2, C14:2-OH, C16, C16-OH, C16:1, C16:1-OH, C16:2, C16:2-OH, C18, C18:1, C18:1-OH, C18:2). However, the number of altered LCACs was more extended from that seen in Bl6 mice. Moreover, the sum of hydroxylated LCACs was significantly elevated. Similar to Bl6 strain, ratios of acylcarnities to free carnitine (CRT-1 and CPT-1) were significantly elevated. Among 129Sv mice 24 h LPS treatment caused a significant change in the levels of biogenic amines serotonin and KYN. The level of serotonin was significantly reduced, whereas the value of KYN was elevated. Furthermore, the ratio KYN/Trp was elevated, whereas the ratio serotonin/Trp was reduced. Moreover, the ratio KYN/alpha-AAA was significantly elevated. From 90 circulating glycerophospholipids (GPLs) the level of 25 GPLs was significantly altered, including reduction of several LysoPC acyls (LysoPC a C17:0, LysoPC a C18:2, LysoPC a C20:3, LysoPC a C20:4) and PC acyl-alkyls (PC ae C36:2, PC ae C36:4, PC ae C38:2, PC ae C38:3, PC ae C38:5, PC ae C40:3), as well as elevation of several PC diacyls (PC aa C32:3, PC aa C34:3, PC aa C36:1, PC aa C36:2, PC aa C38:1, PC aa C38:4, PC aa C38:6, PC aa C40:2, PC aa C40:3, PC aa C40:5, PC aa C40:6, PC aa C42:2, PC aa C42:4, PC aa C42:5). Similar to Bl6 mice, the most significant alteration was the elevation of sphingolipids. It is noteworthy that no changes were observed in the level of hexoses in 129Sv mice. The most significant results are highlighted in **Figure 4**.

#### GLM Analysis of LPS Induced Alterations in Metabolic Profile of Bl6

Paired *t*-tests with Bonferroni correction (*p* value less or equal to 0.0002) were used to first sort out significant differences in metabolite levels. Subsequently, GLM analysis was used to confirm differences between LPS and saline treatment groups in 1.5 and 24 h LPS challenge.

The final GLM model demonstrated a significant main effect of 1.5 h LPS treatment [*F*_(9,6)_ = 5.01, *p* = 0.03] on metabolite levels. Significant shifts in the model included SCAC C3 (*p* < 0.0001), Ala (*p* = 0.0001), Gly (*p* = 0.001), His (*p* = 0.0004), Phe (*p* = 0.001), Pro (*p* < 0.0001), Ser (*p* < 0.0001), Tyr (*p* = 0.002), and biogenic amine Ac-Orn (*p* = 0.001). The most prominent effect was observed between the 1.5 h LPS treatment and decline of C3, Ala, Pro, and Ser (**Table 2**).

**Table 2:** Effect of LPS treatment after 1.5 h on metabolite levels among Bl6 mice.

**Metabolites**	Bl6 1.5 h LPS			
	**Beta (*β*)**	***β* (95% Cl)**	***t*-value**	***p*-value**
**Propionylcarnitine (C3)**	−0.84	−1.15, −0.52	−5.68	< 0.0001
**Alanine (Ala)**	−0.81	−1.15, −0.47	−5.16	0.0001
**Glycine (Gly)**	−0.74	−1.12, −0.35	−4.08	0.001
**Histidine (His)**	−0.78	−1.14, −0.41	−4.59	0.0004
**Phenylalanine (Phe)**	−0.73	−1.12, −0.33	−3.96	0.001
**Proline (Pro)**	−0.86	−1.15, −0.56	−6.20	< 0.0001
**Serine (Ser)**	−0.86	−1.15, −0.56	−6.20	< 0.0001
**Tyrosine (Tyr)**	−0.72	−1.12, −0.32	−3.92	0.002
Acetylornithine (Ac-Orn)	−0.73	−1.12, −0.34	−4.01	0.001

Statistically significant regression coefficients (*β*), confidence intervals (CI) and *t*- and *p* values (derived from GLM analysis) of log_10_-transformed metabolite levels.

The GLM model of 24 h LPS treatment demonstrated robust main effect of treatment [*F*_(17,1)_= 221092, *p* = 0.0017] on selected metabolite levels. The final model retained BW change, citrulline, biogenic amines KYN, and putrescine, GPLs (LysoPC a C16:1, LysoPC a C20:3, LysoPC a C20:4, PC aa C36:1, PC aa C40:6, PC ae C38:2), sphingolipids [SM (OH) C22:1, SM (OH) C22:2, SM C16:0, SM C24:0, SM C24:1], and hexoses (all *p* values < 0.0001; **Supplementary Table 4**). Citrulline, LysoPC acyls, PC diacyls, and hexoses were significantly downregulated after the 24 h LPS challenge. On the other hand, KYN, putrescine, PC ae C38:2, and sphingolipids were significantly upregulated (**Table 3**).

**Table 3:** Effect of LPS treatment after 24 h on metabolite levels among Bl6 mice.

**Metabolites**	Bl6 24 h LPS			
	**Beta (*β*)**	***β* (95% Cl)**	***t*-value**	***p*-value**
Δ body weight	−0.90	−1.12, −0.68	−8.64	< 0.0001
**Citrulline (Cit)**	−0.83	−1.11, −0.54	−6.05	< 0.0001
Acetylornithine (Ac-Orn)	−0.86	−1.12, −0.60	−6.90	< 0.0001
**Kynurenine**	0.87	0.62, 1.12	7.23	< 0.0001
**Putrescine**	0.88	0.64, 1.12	7.80	< 0.0001
**lysoPC a C16:1**	−0.88	−1.12, −0.64	−7.64	< 0.0001
**lysoPC a C20:3**	−0.88	−1.12, −0.64	−7.61	< 0.0001
**lysoPC a C20:4**	−0.81	−1.11, −0.52	−5.78	< 0.0001
**PC aa C36:1**	0.85	0.57, 1.12	6.56	< 0.0001
**PC aa C40:6**	0.85	0.58, 1.12	6.68	< 0.0001
**PC ae C38:2**	−0.85	−1.12, −0.59	−6.74	< 0.0001
**SM (OH) C22:1**	0.92	0.72, 1.12	9.58	< 0.0001
**SM (OH) C22:2**	0.89	0.66, 1.12	8.05	< 0.0001
**SM C16:0**	0.94	0.76, 1.12	11.02	< 0.0001
**SM C24:0**	0.85	0.57, 1.12	6.53	< 0.0001
**SM C24:1**	0.95	0.80, 1.11	12.84	< 0.0001
**Hexoses (H1)**	−0.78	−1.10, −0.45	−5.06	< 0.0001

Statistically significant regression coefficients (*β*), confidence intervals (CI) and *t*- and *p* values (derived from GLM analysis) of log_10_-transformed metabolite levels.

#### GLM Analysis of LPS Induced Alterations in Metabolic Profile of 129Sv

Similar to the Bl6 strain, the GLM model of the 1.5 h LPS challenge in 129Sv displayed strong main effect of treatment [*F*_(7,9)_= 18.29, *p* = 0.0001]. Significantly altered shifts of metabolites included SCACs C2 (*p* < 0.0001), C3 (*p* < 0.0001), C4- (*p* < 0.0001) and C5- (*p* = 0.0002), citrulline (*p* = 0.001), PC aa C42:0 (*p* = 0.006), and SM (OH) C16:1 (*p* = 0.002; **Table 4**). All metabolites altered in the 1.5 h LPS challenge exhibited a decline, except for complex lipids [PC aa C42:0 and SM (OH) C16:1], which were elevated (**Supplementary Table 4**).

**Table 4:** Effect of LPS treatment after 1.5 h on metabolite levels among 129Sv mice.

**Metabolites**	129Sv 1.5 h LPS			
	**Beta (*β*)**	***β* (95% Cl)**	***t*-value**	***p*-value**
**Acetylcarnitine (C2)**	−	−1.14, −0.54	−5.93	< 0.0001
**Propionylcarnitine (C3)**	−0.87	−1.14, −0.60	−6.81	< 0.0001
**Butyryl- and isobutyrylcarnitine (C4-)**	−0.85	−1.14, −0.56	−6.22	< 0.0001
**Isovalerylcarnitine and 2-methybutyrylcarnitine (C5-)**	−0.78	−1.12, −0.43	−4.78	0.0002
**PC aa C42:0**	0.64	0.22, 1.06	3.22	0.006
**SM (OH) C16:1**	0.56	0.11, 1.02	2.64	0.02

Statistically significant regression coefficients (*β*), confidence intervals (CI) and *t*- and *p* values (derived from GLM analysis) of log_10_-transformed metabolite levels.

The 24 h LPS administration also showed strong main effect of treatment [*F*_(16,1)_ = 437.3, *p* = 0.004]. The most significant effect (*t* values > 10, *p* values < 0.0001) was observed in BW change and blood levels of PC diacyls (PC aa C36:1, PC aa C40:6) and sphingolipids [SM (OH) C22:1, SM (OH) C22:2, SM C16:0, SM C16:1 and SM C24:1]. Moderate (*t* values > 5 < 10, *p* values ≤ 0.0001) LPS-induced alterations were observed in C18:1, citrulline, KYN, GPLs (LysoPC a C20:3, PC aa C38:6, PC aa C42:5) and sphingolipids [SM (OH) C14:1, SM (OH) C16:1, SM (OH) C24:1, SM C18:0, SM C18:1 and SM C24:0]. Minor alterations (*t*-values < 5, *p* values < 0.001) included LCACs C14:1, C16 and C16-OH (**Table 5**).

**Table 5:** Effect of LPS treatment after 24 h on metabolite levels among 129Sv mice.

**Metabolites**	129Sv 24 h LPS			
	**Beta (*β*)**	***β* (95% Cl)**	***t*-value**	***p*-value**
Δ body weight	−0.93	−1.12, −0.74	−10.19	< 0.0001
**Tetradecenoylcarnitine (C14:1)**	0.76	0.42, 1.11	4.73	0.0002
**Hexadecanoylcarnitine (C16)**	0.77	0.43, 1.11	4.83	0.0002
**Hydroxyhexadecanoylcarnitine (C16-OH)**	0.76	0.42, 1.10	4.70	0.0002
**Octadecenoylcarnitine (C18:1)**	0.78	0.45, 1.11	5.04	0.0001
**Citrulline (Cit)**	−0.89	−1.13, −0.65	−7.77	< 0.0001
**Kynurenine**	0.87	0.61, 1.13	7.01	< 0.0001
**lysoPC a C20:3**	−0.85	−1.13, −0.56	−6.34	< 0.0001
**PC aa C36:1**	0.93	0.74, 1.12	10.19	< 0.0001
**PC aa C38:6**	0.85	0.58, 1.13	6.52	< 0.0001
**PC aa C40:6**	0.95	0.80, 1.11	12.82	< 0.0001
**PC aa C42:5**	0.80	0.48, 1.12	5.36	< 0.0001
**SM (OH) C14:1**	0.88	0.64, 1.13	7.55	< 0.0001
**SM (OH) C16:1**	0.79	0.47, 1.12	5.23	< 0.0001
**SM (OH) C22:1**	0.95	0.79, 1.11	12.55	< 0.0001
**SM (OH) C22:2**	0.95	0.80, 1.11	12.40	< 0.0001
**SM (OH) C24:1**	0.92	0.72, 1.13	9.73	< 0.0001
**SM C16:0**	0.97	0.84, 1.10	15.98	< 0.0001
**SM C16:1**	0.94	0.75, 1.12	10.60	< 0.0001
**SM C18:0**	0.91	0.70, 1.13	8.91	< 0.0001
**SM C18:1**	0.92	0.71, 1.13	9.39	< 0.0001
**SM C24:0**	0.93	0.72, 1.13	9.74	< 0.0001
**SM C24:1**	0.98	0.88, 1.08	20.55	< 0.0001

Statistically significant regression coefficients (*β*), confidence intervals (CI) and *t*- and *p* values (derived from GLM analysis) of log_10_-transformed metabolite levels.

### Association Between Metabolites, Body Weight, and Temperature Change in Bl6 After LPS Challenge

**Figure 5:**
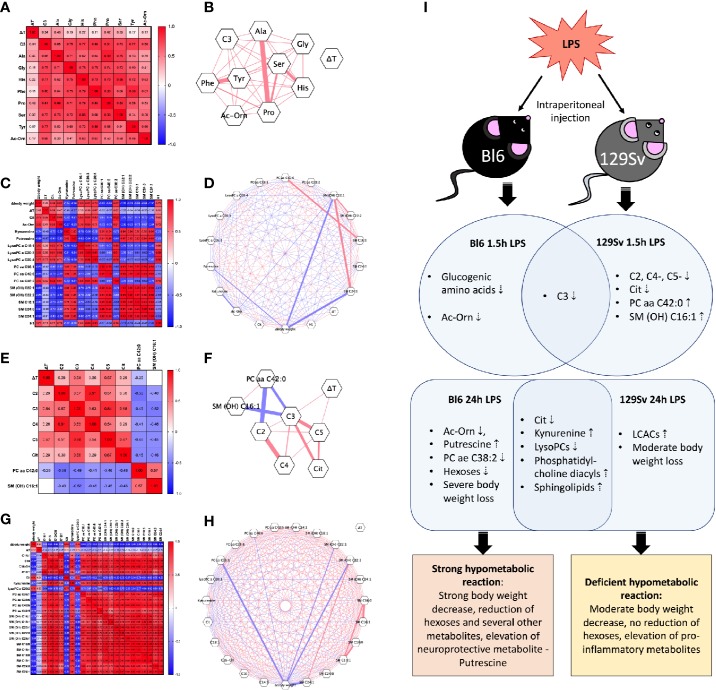
Heat-map of Pearson correlation coefficients between body weight change (Δbody weight), temperature change (ΔT), and all significantly altered metabolites (Log10) in **(A)** Bl6 1.5 h LPS challenge, **(C)** Bl6 24 h LPS challenge, **(E)** 129Sv 1.5 h LPS challenge, and **(G)** 129Sv 24 h LPS challenge. Significant correlations (*p* < 0.05) are highlighted by correlation network diagram in **(B)** Bl6 1.5 h LPS challenge, **(D)** Bl6 24 h LPS challenge, **(F)** 129Sv 1.5 h LPS challenge, and **(H)** 129Sv 24 h LPS challenge. Red lines represent positive and blue lines negative correlations: the strength of the correlation is indicated by line thickness. **(I)** Biomarkers of inflammation 1.5 and 24 h after LPS administration in Bl6 and 129Sv. Data represented in the illustration is derived from the GLM model.

Based on significant results obtained from the GLM analysis, Pearson correlation coefficient matrix was created to measure the relationships between the metabolic profile, BW, and temperature alterations in the 1.5 h and 24 h LPS challenges. Subsequently, correlation networks were constructed based on these matrices. The correlation matrices and networks revealed that some metabolites were positively correlated with each other while others were negatively correlated with each other (**Figures 5A–H**).

Correlation analysis of the 1.5 h LPS challenge in Bl6 mice revealed that all significantly altered levels of metabolites (C3, Ala, Gly, His, Phe, Pro, Ser, Tyr and Ac-Orn) were positively correlated with each other. Strong correlations were observed among amino acids (*r* > 0.53, *p* < 0.05). No significant correlation was established between the 1.5 h body temperature change and metabolites (**Figures 5A, B**). However the 24 h LPS challenge revealed that temperature change was positively correlated with hexoses (*r* = 0.51, *p* = 0.03), citrulline (*r* = 0.66, *p* = 0.001), Ac-Orn (*r* = 0.47, *p* = 0.04), LysoPC a C20:3 (*r* = 0.56, *p* = 0.01), LysoPC a C20:4 (*r* = 0.67, *p* = 0.002) PC ae C38:2 (*r* = 0.62, *p* = 0.005) and negatively correlated with putrescine (*r* = −0.57, *p* = 0.009). Body weight change was positively correlated with citrulline (*r* = 0.76, *p* = 0.0001), Ac-Orn (*r* = 0.82, *p* < 0.0001), LysoPC acyls (*r* > 0.78, *p* < 0.0001), PC ae C38:2 (*r* = 0.82, *p* < 0.0001) and hexoses (*r* = 0.70, *p* = 0.0008), but negatively correlated with KYN (*r* = −0.74, *p* = 0.0002), putrescine (*r* = −0.86, *p* < 0.0001), PC diacyls (*r* < −0.83, *p* < 0.0001), and sphingolipids (*r* < −0.80, *p* < 0.0001). Negative correlation was established between LysoPC acyls and sphingolipids (*r* < −0.52, *p* < 0.05) and a very strong positive correlation was observed between sphingolipids and PC diacyls (*r* > 0.91, *p* < 0.0001; **Figures 5C, D**).

### Association Between Metabolites, Body Weight and Temperature Change in 129Sv After LPS Challenge

Correlation analysis of the 1.5 h LPS challenge in the 129Sv strain revealed significant positive correlation of the 1.5 h body temperature change with SCACs C3 (*r* = 0.54, *p* = 0.03) and C5- (*r* = 0.57, *p* = 0.02). These acylcarnitines showed moderate positive correlation among themselves as well (*r* > 0.51, *p* < 0.05). PC aa C42:0 was negatively correlated with SCACs C2 (*r* = −0.56, *p* = 0.02) and C3 (*r* = −0.49, *p* = 0.04), but positively correlated with SM (OH) C16:1 (*r* = 0.57, *p* = 0.01). Furthermore, positive correlation was established between citrulline and LCACs C3 (*r* = 0.68, *p* = 0.003) and C5- (*r* = 0.67, *p* = 0.003; **Figures 5E, F**).

The 24 h LPS-induced BW change displayed strong positive correlation with citrulline (*r* = 0.80, *p* < 0.0001), lysoPC a C20:3 (*r* = 0.76, *p* = 0.0002) and negative correlation with LCACs (*r* < −0.66, *p* < 0.01), KYN (*r* = −0.71, *p* = 0.0006), PC diacyls (*r* < −0.75, *p* < 0.0001), and sphingolipids (*r* < −0.68, *p* < 0.01). Opposite to the Bl6 strain, no significant correlation was established between 24 h body temperature change and metabolites in 129Sv. Additionally, sphingolipids were positively correlated with LCACs (*r* > 0.51, *p* < 0.05) and PC diacyls (*r* > 0.54, *p* < 0.05). Furthermore, strong positive correlation was observed between LCACs and PC diacyls (*r* > 0.66, *p* < 0.001). Citrulline was negatively correlated with LCACs, KYN, PC diacyls, and sphingolipids. KYN exhibited strong positive correlation with LCACs, PC diacyls, and sphingolipids (**Figures 5G, H**).

## Discussion

Recent studies have demonstrated that metabolomics has become a useful tool for the identification of novel possible biomarkers and their ratios related to different pathological conditions and understanding the molecular mechanisms behind them. Bacterial LPS challenge has been widely used in immunological studies as one of the models of peripherally induced neuroinflammation as well as for modeling depression-like behavior in rodents. However, so far only few studies have examined mouse strain differences in endotoxin-mediated inflammatory response. The purpose of this study was to explore the possible differences in coping with inflammatory processes between Bl6 and 129Sv mouse strains 1.5 h and 24 h after LPS injection. For the association analysis of metabolites, body weight, and temperature change, we chose multivariate GLM analysis that allows us to test the LPS treatment effect on all variables with one test. This analysis takes into account the possible LPS effect on each measured variable and integrates them into one united biological system.

### LPS Depressed Locomotor Activity and Body Weight Decrease

Bl6 and 129Sv strains showed the same overall pattern of motor activity after infection with *E. coli* LPS (0.5 mg/kg, i.p.) as rapid activity reducing effect was observed in both strains (**Figures 2A–C**). Mice became lethargic after LPS administration and displayed depressed locomotor activity throughout 24-h period. Although when splitting 24-h cycle into hourly values, clearly different motor response between Bl6 and 129Sv emerged within 2 h from the beginning of behavioral testing (**Figure 2G**). This could reflect higher anxiety-like behavior of 129Sv at the beginning of the experiment and passive adaptation in stressful environment (Võikar et al., 2001; Abramov et al., 2008; Heinla et al., 2014). Inflammation increases energy demand, and locomotor retardation helps animals conserve energy. Energy is redirected from growth and reproduction programs into maintenance and survival programs (Wang et al., 2019).

LPS induced a significant BW loss in both strains 24 hours after treatment. However, BW loss was more pronounced in Bl6, than in 129Sv mice, even though the starting weight of both strains did not differ. Administration of LPS has been reported to reduce significantly food intake in Bl6 mice (Kim et al., 2013). Furthermore, overnight (18 h) fasting results in about 16% of BW loss in Bl6 strain (Ayala et al., 2006). Thus, the loss of BW may be explained in some extent by reduced food intake.

LPS induced slight reduction of body temperature, although it did not reach statistical significance level. Interestingly, 1.5 h into inflammation, 129Sv display greater decline of body temperature than Bl6. However, as inflammation progressed, the body temperature of 129Sv mice increased, while the body temperature of Bl6 mice was persistently low in the 24 h period. Body temperature of 129Sv 1.5 h after LPS administration was significantly lower compared to the 24 h temperature decline. Impaired thermoregulation could be disadvantageous in the context of immune activation, which could be one of the reasons 129Sv mice seem to be more vulnerable to infection. A recent study demonstrated inflammation-induced hypothermia as a strategy for inducing hypometabolism, which is essential for host tolerance and survival (Ganeshan et al., 2019).

### Basal Metabolic Profile Differences Between Bl6 and 129Sv

Saline-treated Bl6 and 129Sv mice differed in several metabolite concentrations. In both 1.5 and 24 h time points, the levels of biogenic amines (Ac-Orn, alpha-AAA and carnosine) were significantly higher in Bl6 compared to 129Sv. Alpha-AAA is an intermediate in lysine (Lys) metabolic pathway, a marker for oxidative stress and an established biomarker for insulin resistance and diabetes risk (Yuan et al., 2011; Zeitoun-Ghandour et al., 2011; Wang et al., 2013). Several studies in rodents have shown that alpha-AAA is an inhibitor of kynurenic acid (KYNA) synthesis. KYNA is a neuroactive metabolite and antagonist of the glutamatergic N-methyl-D-aspartate (NMDA) as well as AMPA/kainate and alpha 7 nicotinic receptors (Heyes et al., 1992; Wu et al., 1995; Tuboly et al., 2015). The increased level of alpha-AAA in Bl6 mice has been reported to be caused by defect in *Dhtkd1* gene. *Dhtkd1* has been identified as a primary regulator of alpha-AAA and defects in this gene result in the buildup of alpha-AAA (Wu et al., 2014; Leandro et al., 2019). Carnosine is dipeptide (beta-alanyl-L-histidine) that is highly concentrated in excitable tissues such as muscle and brain and is capable of scavenging free radicals (Klebanov et al., 1998). Furthermore, experiments with primary neuronal cultures show neuroprotective properties of carnosine (Bae and Majid, 2013). The higher blood concentrations of these metabolites in Bl6 mice may contribute to their being more capable of coping with infection.

In contrast, one acylcarnitine (C5-) and sphingolipid SM (OH) C22:2 were higher in 129Sv at both 1.5 and 24 h time points. Additionally, the 24 h saline-treated 129Sv mice had higher blood levels of C4- compared to the Bl6 mice, but this difference was missing between the 1.5 h saline treatment groups. This could indicate a possible acute stress-induced reduction of C4- in 129Sv. SCAC C4- is derived from ketone bodies and from ketogenic amino acid Leu, while C5- is the byproduct of branched-chain amino acids (BCAA) Ile and Leu catabolism. The finding that Bl6 mice have higher levels of Ac-Orn, alpha-AAA, carnosine and 129Sv have higher blood levels of C4- and C5- also supports the results of a previous research (Narvik et al., 2018), indicating that these metabolites belong to the metabolomic signature of these strains. C5- acylcarnitine in mouse blood plasma is a mixture of isovalerylcarnitine and 2-methybutyrylcarnitine. Recent evidence suggests that increased levels of C5- acylcarnitine in 129Sv mice are contributed by the accumulation of isovalerylcarnitine, which is caused by a splice site mutation in the *lvd* gene resulting in isovaleryl-CoA dehydrogenase deficiency (Leandro et al., 2019).

### LPS-Induced Metabolite Differences

In addition to LPS-induced body weight differences between strains, metabolites in Bl6, and 129Sv strains were also differently affected by LPS challenge after 1.5 and 24 h. After 1.5 h, the number of affected metabolites was more extensive in Bl6 compared to 129Sv. Nevertheless, inflammatory responses after 24 h included a wide-range of alterations in several different metabolite groups. In this case the number of altered metabolites was substantially larger in 129Sv compared to Bl6.

#### Acylcarnitines

SCACs C3, C4- and C5- were decreased 1.5 h after LPS administration in both strains. Additionally, C2 (acetylcarnitine) was decreased only in 129Sv, and multiple SCACs and MCACs were reduced solely in the Bl6 strain. Previous findings suggest that medium- and long-chain acylcarnitines participate in proinflammatory signaling pathways (Rutkowsky et al., 2014; Ganeshan et al., 2019). Furthermore, several studies have reported alterations of acylcarnitine profile in pathological conditions including type 2 diabetes (Adams et al., 2009), cardiovascular diseases (Makrecka-Kuka et al., 2017), and first episode psychosis (Kriisa et al., 2017). After applying the GLM analysis, only four acylcarnitines remained statistically significant in the 1.5 h LPS challenge group. Decrease of C3 (a metabolite of Val and Ile catabolism) was significant in both strains, while reduction of C2, C4- and C5- was only evident in 129Sv.

The only acylcarnitine, which remained changed throughout the 1.5 and 24 h inflammatory responses in both strains, was C3. Additionally, 24 h after LPS administration several LCACs exhibited significant increase in both strains. Specifically in 129Sv, additional LCACs were significantly increased of which most were hydroxylated acylcarnitines. Furthermore, the sum of hydroxylated LCACs was significantly increased in 129Sv, whereas no alterations were observed in Bl6. Long-chain fatty acids are transported to the mitochondria in the form of acylcarnitines, where *β*-oxidation takes place to produce major portion of metabolic energy. Based on these findings, it seems that LPS induces incomplete long-chain fatty acid *β*-oxidation in 129Sv, which induces accumulation of hydroxylated LCAC intermediates. Besides, due to a certain shift of oxidative catabolism of fatty acids, *e.g.* intensification of omega- and alpha-oxidation pathways, some portion of hydroxylated acylcarnitines could be produced.

The 24 h LPS challenge caused a reduction of SCAC C4- in Bl6 as well as a reduction of free carnitine (C0) in 129Sv. The increase in plasma acylcarnitines and decrease in plasma carnitine suggest increased utilization of carnitine for the production of acylcarnitines in 129Sv. GLM analysis for the 24 h LPS challenge revealed significant alterations in acylcarnitine profile only in 129Sv, implying that changes in acylcarnitine levels after LPS administration are specific for 129Sv.

#### Amino Acids

Citrulline decreased in both strains compared to the saline-treated control groups 1.5 h after LPS administration. Aside from citrulline, no other amino acids were altered in 129Sv. Contrary to this stability in 129Sv mice, Bl6 mice exhibited a reduction in various amino acids (Ala, Gly, His, Phe, Met, Pro, Ser, Thr, Val). Combination of several factors (intensification of synthesis of protector proteins as well as inflammatory mediators, illness-associated malnutrition, intensification of production of ketone bodies, and glyconeogenesis) may cause the above-mentioned declines as Phe, Met, Thr, and Val are essential amino acids, Ala, Gly, His, Met, Pro, Ser, Thr and Val are glucogenic amino acids, and Phe is a gluco-ketogenic amino acid. Therefore, one single factor, like short-term (1.5 h) fasting should not cause a depletion of plasma amino acid levels (Felig et al., 1969). Furthermore, the sum of glucogenic amino acids was significantly reduced in Bl6, whereas no significant changes were found in 129Sv 1.5 h after LPS administration. GLM analysis confirmed that amino acids were specifically reduced in Bl6 1.5 h after LPS administration. Although this profile was somewhat different, including Ala, Gly, His, Phe, Met, Pro and Tyr.

24 h post treatment, only the reduction of citrulline remained statistically significant. Citrulline is one of the key products of Arg catabolism. Nitric oxide synthase (NOS) catalyzes Arg hydrolysis into citrulline and NO. LPS and cytokines are known to induce NOS expression and thus the production of NO (Förstermann and Sessa, 2012). However decreased concentration of citrulline indicates decreased NOS activity and NO production. This was further supported by a decrease of plasma ratio of citrulline/Arg in both strains, which reflects decreased activity of NOS. Furthermore, plasma levels of NOS inhibitors ADMA and SDMA were significantly elevated in the LPS treated Bl6 mice after 24 h, but not in 129Sv. However, it is worth to underline that the plasma concentrations of ADMA and SDMA in 129Sv were already slightly higher in control conditions, resembling Bl6 blood concentrations in inflammatory conditions, which has been also noted previously in these strains in home cage conditions (Narvik et al., 2018). This reflects increased NOS inhibition and thus decreased NO and citrulline production. Low plasma citrulline levels have been previously associated with acute respiratory distress syndrome in sepsis patients (Ware et al., 2013).

#### Biogenic Amines

Alpha-AAA, Ac-Orn, and putrescine are examples of significantly reduced biogenic amines in Bl6 mice 1.5 h after LPS administration. In contrast, the ratio of spermidine/putrescine was elevated in Bl6, indicating increased activity of spermidine synthase and conversion of putrescine to spermidine. No alterations in the biogenic amine profile were observed in 129Sv. As mentioned above alpha-AAA and Ac-Orn are metabolites found more abundant in the blood samples of Bl6 compared to 129Sv in baseline conditions.

LPS-response-specific biogenic amines that were altered in both strains 24 h after LPS administration included KYN and serotonin. While the concentration of serotonin was decreased, the level of KYN in the blood plasma was significantly higher compared to saline-treated mice. Trp is metabolized in the KYN and serotonin pathway. Although no differences were observed in plasma Trp levels of the LPS-treated mice, there was a shift favoring the ratio of KYN/Trp to the ratio of serotonin/Trp. This indicates that Trp metabolism through the KYN pathway is favored during inflammatory conditions. Trp conversion to KYN is catalyzed by the enzyme indoleamine 2,3-dioxygenase (IDO), which is activated by inflammatory cytokines and it has been suggested that plasma levels of KYN directly reflect the activity of IDO (Moffett and Namboodiri, 2003). KYN can be further converted into neuroprotective KYNA or neurotoxic quinolinic acid, which is intermediate for nicotinamide adenine dinucleotide (NAD+) biosynthesis. Blood KYN is transported extensively to brain. About 80% of blood KYN is transported to brain in normal conditions and this is even further amplified reaching up to 98% in inflammatory conditions (Kita et al., 2002). Since KYN competes with Leu for the transport from the blood to the brain *via* large amino transporter LAT1, the fact that the ratio Leu/KYN was in favor of KYN in inflammatory conditions 24 h after LPS administration, may indicate that KYN is able to cross the blood-brain barrier (BBB) more efficiently. This ratio was only significantly altered in Bl6 mice. Whether the KYN pathway favors the shift towards KYNA or quinolinic acid in LPS-induced neuroinflammatory conditions in these strains still needs further investigation. However, KYN and alpha-AAA are both substrates for kynurenine aminotransferase II (KAT-II; also known as alpha-AAA aminotransferase II), which is responsible for the transamination of KYN into KYNA (Buchli et al., 1995; Hallen et al., 2013), and thus alpha-AAA levels indicate the availability of KAT-II for the transamination of KYN. The ratio KYN/alpha-AAA was again in favor of KYN in inflammatory conditions 24 h after LPS administration providing additional insight that KYN may be transaminated to KYNA. Individual differences between strains included reduction of Ac-Orn and increase in putrescine, ADMA and SDMA in Bl6, whereas no strain-specific alterations were observed in 129Sv. After applying GLM, only the reduction of Ac-Orn and elevation of putrescine remained statistically significant in Bl6, and elevation of KYN remained significant in both strains. Together with Arg, Ac-Orn is one of the precursors for ornithine synthesis and from there on, putrescine is synthesized from ornithine. The decrease in Ac-Orn was also accompanied by a slight reduction of plasma ornithine in Bl6 mice. Increased values of putrescine and decreased values of ornithine and Ac-Orn suggest increased putrescine biosynthesis in the later phases of inflammatory response in Bl6 mice. Additionally, the ratio between spermidine and putrescine was decreased in Bl6, indicating deteriorated activity of spermidine synthase and conversion of putrescine to spermidine. Alterations in putrescine synthesis pathway were absent in 129Sv. Putrescine has been shown to possess neuroprotective activity in the CNS (Jänne et al., 2005). Whether Bl6 mice are more capable in coping with LPS-induced neuroinflammation still needs further investigation.

#### Glycerophospholipids and Sphingolipids

Glycerophospholipids (GPLs) and sphingolipids (sphingomyelines, SMs) are considered inflammatory mediators, and altered levels of lipids reflect interplay between inflammation and lipid metabolism. Prominent parts of the GPL family are phoshatidylcholines (PCs), which are main precursors for LysoPCs. In the 1.5 h LPS challenge, the only alteration observed was the elevation of PC aa C42:0 in 129Sv. After utilization of the GLM analysis, SM (OH) C16:1 was included to the list of significantly elevated lipids in 129Sv. No significant alterations in lipid metabolism were observed in Bl6.

In the 24 h post LPS administration, the most striking alteration in both strains was a decrease in LysoPCs and an increase in SMs. Additionally, several PC diacyls were elevated and PC acyl-alkyls were reduced in both strains. Inflammatory response caused the production of different patterning of these metabolites *via* the influence of action of several enzymes in the metabolic network; as LysoPCs decrease, SM and PCs increase. However, the number of elevated SMs and PCs was significantly higher in 129Sv. Specifically, in Bl6, LysoPC a C16:1 and LysoPC a C18:1 were decreased, which was accompanied by an increase in plasma PC aa C34:2. This could indicate disturbances in hydrolysis of PC aa C34:2 into LysoPC a C16:1 and LysoPC a C18:1 in Bl6. Furthermore, the ratios between LysoPCs C16:1/C16:0 and C18:2/C18:1 were significantly lower after LPS administration, whereas no significant alterations in these lysoPC ratios were observed in 129Sv. It has been proposed that lysoPC a C16:0 and lysoPC a C18:0 have the capacity to promote cytokine response *in vitro* (Bach et al., 2010). These changes were missing in 129Sv, which may indicate an increased cytokine response in 129Sv. GPLs and SMs are components of membrane bilayers, which mediates the first line of defense by establishing a physical barrier against pathogens and contains cell surface receptors such as Toll-like receptor 4 (TLR 4) that plays a key role in LPS-mediated signal transduction. Recent evidence suggests that several GPLs and SMs are potential biomarkers for cardiovascular disease (Paapstel et al., 2018) and first-episode psychosis (Leppik et al., 2019).

#### Monosaccharides

Hexoses were significantly reduced in Bl6, whereas no alterations were observed in 129Sv 24 h post infection with LPS. Metabolic energy is generated from the breakdown of monosaccharides, making them an essential source of energy, especially for the brain. Diminished levels of hexoses, including glucose, imply that inflammation contributes to glucose metabolism disorder in Bl6 mice. This suggests that metabolism in Bl6 mice in LPS-induced inflammatory conditions is slowed down. LPS-induced lower levels of blood glucose have been described previously in Bl6 mice (Ganeshan et al., 2019). Hypometabolic condition is important for conserving metabolic energy in inflammatory conditions. Inflammatory response is energetically expensive and requires redistribution of nutrients to support immune activation (Ganeshan and Chawla, 2014). Recent findings suggest that hypometabolic response to endotoxemia is essential for utilization of tissue tolerance as a defense against bacterial pathogens (Ganeshan et al., 2019). It seems that energy conserving hypometabolic state is one possible mechanism used by Bl6 mice to cope with inflammation.

Correlation analysis was performed to describe the nature and strength of the relationship between body weight decline and metabolites. We demonstrated that a 24 h change of body weight in both strains is significantly correlated with changes in metabolites. In Bl6, body weight loss was significantly positively correlated with number of metabolites (citrulline, Ac-Orn, various LysoPC acyls, PC ae 38:2 and hexoses), meaning that severe BW decline was accompanied by a significant decrease in these metabolites. However, few metabolites (KYN, putrescine, PC diacyls, and SMs) displayed negative correlation with weight loss. Similarly, to Bl6, 129Sv demonstrated a significant negative correlation between BW loss and KYN, PC diacyls and SMs. However, in the case of 129Sv, the number of elevated PC diacyls and SMs was considerably higher, emphasizing wider upregulation of inflammatory mediators. Furthermore, the BW loss of 129Sv was also negatively correlated with LCACs, and only two metabolites (citrulline and lysoPC a C20:3) displayed positive correlation with body weight decline. This demonstrates that body weight loss is related to changes in metabolite profile in both strains, and the greater weight loss of Bl6 may contribute to hypometabolism and beneficial alterations of metabolic profile in response to inflammation. Whereas the moderate body weight decrease of 129Sv is mostly associated with excess production of inflammatory mediators.

## Conclusion

Although many of the metabolic shifts caused by acute LPS treatment were similar between Bl6 and 129Sv mouse strains in 1.5 and 24 h, we determined several strain-specific metabolic alterations. 1.5 h after LPS administration, Bl6 exhibited hypometabolism of glucogenic amino acids as well as Ac-Orn. This hypometabolic reaction was further intensified after 24 h, as Ac-Orn, PC ae 38:2, LysoPC a C16:1, LysoPC a C18:1, hexoses, and the ratio between LysoPCs (C16:1 and C16:0) were significantly lower in the LPS treated animals than the saline controls. Furthermore, we demonstrated increased production of putrescine in Bl6 mice, which is known to retain neuroprotective properties (**Figure 5I**).

In 129Sv, elevation of PC aa C42:0 and SM (OH) C16:1 after 1.5 h LPS administration points to disturbances in lipid signaling pathways. Along with elevation of lipids, 129Sv displayed a significant decrease in SCACs and citrulline. 24 h later, LPS administration led to an increase in hydroxylated LCACs which was accompanied by moderate loss of BW in 129Sv. Multiple studies have reported that LCACs can activate proinflammatory signaling pathways (Kouttab and De Simone, 1993; Rutkowsky et al., 2014; McCoin et al., 2015). Thus, the elevation of LCACs in 129Sv could indicate increased inflammatory status (**Figure 5I**).

Our data suggests that Bl6 is actively coping with inflammation by suppressing normal production of metabolites and enhancing the production of neuroprotective metabolites, which is accompanied by stronger BW loss. On the contrary, the BW loss of 129Sv was less severe, and metabolism appears to enhance the proinflammatory status by upregulating the levels of hydroxylated LCACs. This might suggest that LPS causes stronger hypometabolic state in Bl6 mice than in the 129Sv strain. Altogether, this study confirms that Bl6 and 129Sv mice display vastly distinct adaptation capacities independent from the nature of stressful challenge.

## Data Availability Statement

All datasets generated for this study are included in the article/**Supplementary Material**.

## Ethics Statement

The animal study was reviewed and approved by Estonian National Board of Animal Experiments.

## Author Contributions

All authors participated in the experiments of the study and review of the manuscript. EV, MZ, KL, and MP contributed to the conception and design of the study. MP and KL conducted the behavioral experiments. ET made FIA-MS/MS and acute treatment with amphetamine elicits different response LC-MS/MS measurements. EV and MP preformed statistical analysis. MP wrote the first draft of the manuscript. EV, MZ, KL, LT, and ET contributed to manuscript revision, read, and approved the submitted version.

## Funding

This research was supported by the European Union through the European Regional Development Fund (Project No. 2014-2020.4.01.15-0012 and Mobilitas Pluss Program No. MOBTT77) and by team grant PRG685 from the Estonian Research Council.

## Conflict of Interest

The authors declare that the research was carried out in the absence of any personal, professional or financial relationships that could be construed as a potential conflict of interest.
