# Supplementary material for: Treatment With Lipopolysaccharide Induces Distinct Changes in Metabolite Profile and Body Weight in 129Sv and Bl6 Mouse Strains
Source: Front Pharmacol. 2020 Mar 27;11:371. doi: 10.3389/fphar.2020.00371 (PMC7118216; doi:10.3389/fphar.2020.00371)
Supplement: Supplementary file 1 [file DataSheet_1.docx]

Supplementary Material

# Supplementary Table 1

Two-way ANOVA [treatment (saline or LPS) x time (1.5 h or 24 h LPS challenge)] summary table of 1.5 h and 24 h LPS induced changes in metabolite levels (log10 values, median and range) and their ratios in Bl6 strain. Bonferroni post-hoc test was used for multiple comparisons. * Statistically significant difference between control and LPS in 1.5 or 24 administration groups; ^#^ Statistically significant difference between LPS administration groups. ^@^ Statistically significant difference between saline administration groups. * p ≤ 0.05; ** p ≤ 0.01; *** p ≤ 0.001; **** p ≤ 0.0001

| **Metabolites** | **Bl6** | | | | **Two-way ANOVA** | | |
| --- | --- | --- | --- | --- | --- | --- | --- |
|  | **1.5 h** | | **24 h** | |  |  |  |
|  | **Control**  **Median**  **(min – max)** | **LPS**  **Median**  **(min – max)** | **Control**  **Median**  **(min – max)** | **LPS**  **Median**  **(min – max)** |  |  |  |
|  |  |  |  |  |  | ***F*** | ***p*** |
| **Acylcarnitines** | | | | | | | |
| Carnitine (C0) | 1.54  1.44-1.64 | 1.49  1.40-1.55 | 1.44  1.36-1.56 | 1.34^#^  1.09-1.53 | **Treatment** | 7.82 | 0.0087 |
|  |  |  |  |  | **Time** | 14.57 | 0.00059 |
|  |  |  |  |  | **Treatment X time** | 0.84 | 0.37 |
| Acetylcarnitine (C2) | 1.31  1.22-1.40 | 1.27  1.15-1.52 | 1.24  1.00-1.35 | 1.16  1.07-1.43 | **Treatment** | 0.06 | 0.81 |
|  |  |  |  |  | **Time** | 5.13 | 0.03 |
|  |  |  |  |  | **Treatment X time** | 0.00 | 0.99 |
| Propionylcarnitine (C3) | -0.06  -0.16-0.08 | -0.23**  -0.35-(-0.21) | -0.14  -0.26-0.10 | -0.19*  -0.46-(-0.05) | **Treatment** | 22.98 | <0.0001 |
|  |  |  |  |  | **Time** | 0.16 | 0.69 |
|  |  |  |  |  | **Treatment X time** | 0.60 | 0.45 |
| Hydroxybutyryl-carnitine (C3-DC) | -0.93  -1.07-(-0.89) | -1.00  -1.01-(-0.77) | -1.03  -1.08-(-0.89) | -1.00  -1.04-(-0.79) | **Treatment** | 0.33 | 0.57 |
|  |  |  |  |  | **Time** | 0.39 | 0.54 |
|  |  |  |  |  | **Treatment X time** | 2.37 | 0.13 |
| Hydroxypropionyl-carnitine (C3-OH) | -1.09  -1.19-(-0.86) | -1.21  -1.41-(-0.99) | -1.13  -1.32-(-0.77) | -1.20  -1.36-(-0.79) | **Treatment** | 1.8 | 0.19 |
|  |  |  |  |  | **Time** | 0.03 | 0.86 |
|  |  |  |  |  | **Treatment X time** | 0.81 | 0.37 |
| Butyryl- and isobutyrylcarnitine (C4-) | -0.08  -0.19-(-0.03) | -0.20**  -0.29-(-0.11) | -0.18  -0.23-(-0.05) | -0.24**  -0.33-(-0.16) | **Treatment** | 25.47 | <0.0001 |
|  |  |  |  |  | **Time** | 7.92 | 0.008 |
|  |  |  |  |  | **Treatment X time** | 0.005 | 0.94 |
| Propenoylcarnitine (C3:1) | -1.26  -1.35-(-1.09) | -1.35*  -1.47-(-1.19) | -1.24  -1.30-(-1.13) | -1.18^#^  -1.32-(-1.12) | **Treatment** | 2.40 | 0.13 |
|  |  |  |  |  | **Time** | 3.93 | 0.06 |
|  |  |  |  |  | **Treatment X time** | 7.65 | 0.009 |
| Butenoylcarnitine (C4:1) | -0.96  -1.04-(-0.92) | -1.05*  -1.15-(-1.00) | -1.03  -1.12-(-0.94) | -1.02  -1.14-(-0.97) | **Treatment** | 4.35 | 0.04 |
|  |  |  |  |  | **Time** | 0.77 | 0.39 |
|  |  |  |  |  | **Treatment X time** | 7.19 | 0.01 |
| Isovalerylcarnitine and 2-methybutyrylcarnitine (C5-) | -0.57  -0.67-(-0.47) | -0.69**  -0.82-(-0.62) | -0.63  -0.69-(-0.50) | -0.60  -0.78-(-0.53) | **Treatment** | 7.74 | 0.009 |
|  |  |  |  |  | **Time** | 0.75 | 0.39 |
|  |  |  |  |  | **Treatment X time** | 7.11 | 0.01 |
| Glutarylcarnitine (C5-DC) | -1.44  -1.60-(-1.22) | -1.52  -1.70-(-1.46) | -1.55  -1.70-(-1.37) | -1.48  -1.64-(-1.44) | **Treatment** | 1.38 | 0.25 |
|  |  |  |  |  | **Time** | 0.634 | 0.43 |
|  |  |  |  |  | **Treatment X time** | 4.36 | 0.04 |
| Hydroxyvaleryl-carnitine (C5-OH) | -0.97  -1.01-(-0.83) | -0.99  -1.11-(-0.96) | -1.01  -1.08-(-0.85) | -0.94  -1.14-(-0.83) | **Treatment** | 1.33 | 0.26 |
|  |  |  |  |  | **Time** | 0.002 | 0.96 |
|  |  |  |  |  | **Treatment X time** | 2.03 | 0.16 |
| Tiglylcarnitine (C5:1) | -1.12  -1.14-(-1.01) | -1.20*  -1.34-(-1.15) | -1.19  -1.31-(-1.07) | -1.14  -1.24-(-1.01) | **Treatment** | 1.76 | 0.19 |
|  |  |  |  |  | **Time** | 0.0006 | 0.98 |
|  |  |  |  |  | **Treatment X time** | 11.41 | 0.002 |
| Glutaconylcarnitine (C5:1-DC) | -1.44  -1.55-(-1.26) | -1.47  -1.66-(-1.43) | -1.47  -1.57-(-1.32) | -1.52  -1.77-(-1.40) | **Treatment** | 6.44 | 0.02 |
|  |  |  |  |  | **Time** | 1.57 | 0.22 |
|  |  |  |  |  | **Treatment X time** | 0.18 | 0.67 |
| Hexanoylcarnitine (C6) | -1.64  -1.72-(-1.54) | -1.61  -1.68-(-1.42) | -1.61  -1.74-(-1.52) | -1.54  -1.72-(-1.46) | **Treatment** | 2.35 | 0.14 |
|  |  |  |  |  | **Time** | 0.45 | 0.51 |
|  |  |  |  |  | **Treatment X time** | 0.01 | 0.91 |
| Hexenoylcarnitine (C6:1) | -1.96  -2.00-(-1.89) | -2.00*  -2.10-(-1.96) | -2.00  -2.05-(-1.96) | -1.96  -2.10-(-1.92) | **Treatment** | 1.13 | 0.30 |
|  |  |  |  |  | **Time** | 0.16 | 0.69 |
|  |  |  |  |  | **Treatment X time** | 10.48 | 0.003 |
| Pimelylcarnitine (C7-DC) | -1.49  -1.68-(-1.38) | -1.49  -1.64-(-1.42) | -1.61  -1.77-(-1.49) | -1.52  -1.64-(-1.32) | **Treatment** | 2.05 | 0.16 |
|  |  |  |  |  | **Time** | 3.23 | 0.08 |
|  |  |  |  |  | **Treatment X time** | 2.43 | 0.13 |
| Octanoylcarnitine (C8) | -0.78  -0.89-(-0.71) | -0.88  -0.96-(-0.76) | -0.83  -0.91-(-0.76) | -0.81  -0.90-(-0.78) | **Treatment** | 2.59 | 0.12 |
|  |  |  |  |  | **Time** | 0.001 | 0.98 |
|  |  |  |  |  | **Treatment X time** | 4.66 | 0.04 |
| Nonanoylcarnitine (C9) | -1.05  -1.20-(-0.89) | -1.07  -1.15-(-1.00) | -1.03  -1.21-(-0.95) | -1.06  -1.12-(-0.99) | **Treatment** | 0.17 | 0.68 |
|  |  |  |  |  | **Time** | 0.40 | 0.53 |
|  |  |  |  |  | **Treatment X time** | 0.01 | 0.91 |
| Decanoylcarnitine (C10) | -0.49  -0.62-(-0.39) | -0.56  -0.72-(-0.44) | -0.45  -0.60-(-0.37) | -0.46^#^  -0.58-(-0.32) | **Treatment** | 1.54 | 0.22 |
|  |  |  |  |  | **Time** | 8.52 | 0.006 |
|  |  |  |  |  | **Treatment X time** | 2.30 | 0.14 |
| Decenoylcarnitine (C10:1) | -0.98  -1.01-(-0.82) | -1.02  -1.12-(-0.91) | -1.04  -1.12-(-0.90) | -0.99  -1.04-(-0.85) | **Treatment** | 0.08 | 0.78 |
|  |  |  |  |  | **Time** | 0.13 | 0.72 |
|  |  |  |  |  | **Treatment X time** | 4.80 | 0.04 |
| Decadienyl-carnitine (C10:2) | -0.89  -1.03-(-0.77) | -0.93  -0.97-(-0.86) | -0.91  -0.99-(-0.82) | -0.91  -1.12-(-0.85) | **Treatment** | 1.66 | 0.21 |
|  |  |  |  |  | **Time** | 0.73 | 0.40 |
|  |  |  |  |  | **Treatment X time** | 0.07 | 0.79 |
| Dodecanoyl-carnitine (C12) | -1.00  -1.05-(-0.82) | -1.07  -1.14-(-0.83) | -1.02  -1.19-(-0.79) | -1.11  -1.17-(-0.81) | **Treatment** | 1.84 | 0.18 |
|  |  |  |  |  | **Time** | 0.28 | 0.60 |
|  |  |  |  |  | **Treatment X time** | 0.38 | 0.54 |
| Dodecanedioyl-carnitine C12-DC | -0.41  -0.50-(-0.36) | -0.51**  -0.53-(-0.45) | -0.47  -0.54-(-0.40) | -0.47  -0.56-(-0.42) | **Treatment** | 9.33 | 0.005 |
|  |  |  |  |  | **Time** | 1.30 | 0.26 |
|  |  |  |  |  | **Treatment X time** | 5.12 | 0.03 |
| Dodecenoyl-carnitine (C12:1) | -0.84  -1.09-(-0.74) | -0.83  -0.98-(-0.55) | -0.88  -0.95-(-0.81) | -0.88  -0.98-(-0.52) | **Treatment** | 1.97 | 0.17 |
|  |  |  |  |  | **Time** | 1.17 | 0.68 |
|  |  |  |  |  | **Treatment X time** | 0.0002 | 0.99 |
| Tetradecanoyl-carnitine (C14) | -1.34  -1.42-(-1.28) | -1.34  -1.52-(-0.96) | -1.40  -1.66-(-1.24) | -1.30  -1.57-(-0.91) | **Treatment** | 4.30 | 0.046 |
|  |  |  |  |  | **Time** | 0.29 | 0.59 |
|  |  |  |  |  | **Treatment X time** | 2.20 | 0.15 |
| Tetradecenoyl-carnitine (C14:1) | -1.48  -1.54-(-1.41) | -1.41  -1.54-(-1.23) | -1.51  -1.55-(-1.34) | -1.39  -1.57-(-0.98) | **Treatment** | 4.66 | 0.04 |
|  |  |  |  |  | **Time** | 0.48 | 0.50 |
|  |  |  |  |  | **Treatment X time** | 2.35 | 0.13 |
| Hydroxytetra-decenoylcarnitine (C14:1-OH) | -1.72  -2.00-(-1.51) | -1.76  -1.92-(-1.66) | -1.78  -1.92-(-1.59) | -1.72  -1.85-(-1.49) | **Treatment** | 0.34 | 0.57 |
|  |  |  |  |  | **Time** | 0.12 | 0.73 |
|  |  |  |  |  | **Treatment X time** | 1.16 | 0.29 |
| Tetradecadienyl-carnitine (C14:2) | -1.89  -2.10-(-1.66) | -1.85  -2.00-(-1.66) | -1.96  -2.10-(-1.77) | -1.89  -2.10-(-1.49) | **Treatment** | 1.88 | 0.18 |
|  |  |  |  |  | **Time** | 0.27 | 0.61 |
|  |  |  |  |  | **Treatment X time** | 0.39 | 0.54 |
| Hydroxytetra-decadienylcarnitine C14:2-OH | -1.82  -1.96-(-1.57) | -1.96*  -2.00-(-1.72) | -1.84  -1.96-(-1.70) | -1.85  -1.96-(-1.74) | **Treatment** | 7.89 | 0.008 |
|  |  |  |  |  | **Time** | 0.03 | 0.88 |
|  |  |  |  |  | **Treatment X time** | 4.57 | 0.04 |
| Hexadecanoyl-carnitine (C16) | -0.74  -0.91-(-0.67) | -0.64  -0.93-(-0.44) | -0.88  -1.16-(-0.67) | -0.67**  -0.89-(-0.38) | **Treatment** | 14.57 | 0.0006 |
|  |  |  |  |  | **Time** | 0.56 | 0.46 |
|  |  |  |  |  | **Treatment X time** | 2.17 | 0.15 |
| Hydroxyhexa-decanoylcarnitine (C16-OH) | -1.32  -1.54-(-1.08) | -1.40  -1.55-(-1.31) | -1.31  -1.44-(-1.26) | -1.28  -1.41-(-1.19) | **Treatment** | 0.37 | 0.55 |
|  |  |  |  |  | **Time** | 3.54 | 0.07 |
|  |  |  |  |  | **Treatment X time** | 3.32 | 0.08 |
| Hexadecenoyl-carnitine (C16:1) | -1.28  -1.38-(-1.17) | -1.24  -1.41-(-0.98) | -1.35  -1.59-(-1.09) | -1.17*  -1.43-(-0.80) | **Treatment** | 6.35 | 0.02 |
|  |  |  |  |  | **Time** | 0.0002 | 0.99 |
|  |  |  |  |  | **Treatment X time** | 2.76 | 0.11 |
| Hydroxyhexa-decenoylcarnitine (C16:1-OH) | -1.66  -1.70-(-1.42) | -1.72  -1.80-(-1.60) | -1.71  -1.80-(-1.57) | -1.66  -1.72-(-1.46) | **Treatment** | 0.10 | 0.75 |
|  |  |  |  |  | **Time** | 0.08 | 0.78 |
|  |  |  |  |  | **Treatment X time** | 7.75 | 0.009 |
| Hexadecadienyl-carnitine C16:2 | -1.52  -1.59-(-1.21) | -1.53  -1.74-(-1.16) | -1.52  -1.72-(-1.39) | -1.42  -1.60-(-1.22) | **Treatment** | 1.24 | 0.27 |
|  |  |  |  |  | **Time** | 0.005 | 0.95 |
|  |  |  |  |  | **Treatment X time** | 1.84 | 0.18 |
| Hydroxyhexa-decadienylcarnitine (C16:2-OH) | -1.57  -1.70-(-1.41) | -1.62  -1.77-(-1.47) | -1.57  -1.68-(-1.48) | -1.52  -1.66-(-1.47) | **Treatment** | 0.75 | 0.39 |
|  |  |  |  |  | **Time** | 0.92 | 0.35 |
|  |  |  |  |  | **Treatment X time** | 2.88 | 0.10 |
| Octadecanoylcarnitine (C18) | -1.29  -1.40-(-1.28) | -1.27  -1.46-(-1.11) | -1.46^@^  -1.64-(-1.34) | -1.19****^#^  -1.40-(-1.00) | **Treatment** | 26.17 | <0.0001 |
|  |  |  |  |  | **Time** | 0.09 | 0.77 |
|  |  |  |  |  | **Treatment X time** | 19.36 | 0.0001 |
| Octadecenoyl-carnitine (C18:1) | -0.92  -1.15-(-0.87) | -0.89  -1.10-(-0.58) | -0.92  -1.15-(-0.87) | -0.89***  -1.10-(-0.58) | **Treatment** | 20.87 | <0.0001 |
|  |  |  |  |  | **Time** | 0.04 | 0.85 |
|  |  |  |  |  | **Treatment X time** | 5.53 | 0.03 |
| Hydroxyocta-decenoylcarnitine (C18:1-OH) | -1.49  -1.60-(-1.36) | -1.48  -1.68-(-1.29) | -1.55  -1.59-(-1.42) | -1.41  -1.64-(-1.30) | **Treatment** | 3.38 | 0.08 |
|  |  |  |  |  | **Time** | 0.09 | 0.77 |
|  |  |  |  |  | **Treatment X time** | 3.44 | 0.07 |
| Octadecadienyl-carnitine (C18:2) | -1.30  -1.44-(-1.14) | -1.18  -1.44-(-0.86) | -1.44  -1.54-(-1.12) | -1.08***  -1.37-(-0.79) | **Treatment** | 20.68 | <0.0001 |
|  |  |  |  |  | **Time** | 0.03 | 0.86 |
|  |  |  |  |  | **Treatment X time** | 5.17 | 0.03 |
| **Amino Acids** | | | | | | | |
| Alanine (Ala) | 2.64  2.57-2.79 | 2.48*  2.41-2.59 | 2.73  2.54-2.98 | 2.79^###^  2.55-2.90 | **Treatment** | 3.73 | 0.06 |
|  |  |  |  |  | **Time** | 25.49 | <0.0001 |
|  |  |  |  |  | **Treatment X time** | 4.60 | 0.04 |
| Arginine (Arg) | 2.16  2.02-2.22 | 1.97  1.55-2.20 | 2.18  2.04-2.59 | 2.26^##^  1.89-2.59 | **Treatment** | 1.87 | 0.18 |
|  |  |  |  |  | **Time** | 12.38 | 0.001 |
|  |  |  |  |  | **Treatment X time** | 4.19 | 0.05 |
| Asparagine (Asn) | 1.56  1.39-1.89 | 1.43  1.27-1.52 | 1.65  1.55-2.17 | 1.70^##^  1.51-1.82 | **Treatment** | 5.18 | 0.03 |
|  |  |  |  |  | **Time** | 15.22 | 0.0005 |
|  |  |  |  |  | **Treatment X time** | 1.50 | 0.23 |
| Aspartate (Asp) | 1.86  1.66-2.14 | 1.79  1.57-2.06 | 1.87  1.73-2.20 | 1.74  1.58-1.92 | **Treatment** | 3.89 | 0.06 |
|  |  |  |  |  | **Time** | 0.35 | 0.56 |
|  |  |  |  |  | **Treatment X time** | 0.52 | 0.48 |
| Citrulline (Cit) | 1.65  1.58-1.82 | 1.58*  1.47-1.66 | 1.82  1.60-1.93 | 1.52****  1.40-1.65 | **Treatment** | 44.31 | <0.0001 |
|  |  |  |  |  | **Time** | 0.73 | 0.40 |
|  |  |  |  |  | **Treatment X time** | 5.73 | 0.02 |
| Glutamine (Gln) | 2.81  2.70-2.93 | 2.75  2.54-2.85 | 2.80  2.64-3.00 | 2.79  2.74-2.90 | **Treatment** | 2.86 | 0.10 |
|  |  |  |  |  | **Time** | 1.21 | 0.28 |
|  |  |  |  |  | **Treatment X time** | 2.33 | 0.14 |
| Glutamate (Glu) | 2.13  1.93-2.37 | 2.08  1.84-2.61 | 2.20  1.87-2.54 | 2.04  1.88-2.20 | **Treatment** | 0.97 | 0.33 |
|  |  |  |  |  | **Time** | 4.19 | 0.049 |
|  |  |  |  |  | **Treatment X time** | 1.47 | 0.23 |
| Glycine (Gly) | 2.54  2.42-2.65 | 2.43*  2.36-2.48 | 2.59  2.43-2.71 | 2.54  2.28-2.64 | **Treatment** | 10.33 | 0.003 |
|  |  |  |  |  | **Time** | 7.36 | 0.01 |
|  |  |  |  |  | **Treatment X time** | 0.69 | 0.41 |
| Histidine (His) | 1.96  1.89-2.04 | 1.85*  1.79-1.94 | 2.00  1.81-2.20 | 1.97^#^  1.88-2.06 | **Treatment** | 7.32 | 0.01 |
|  |  |  |  |  | **Time** | 11.18 | 0.002 |
|  |  |  |  |  | **Treatment X time** | 1.86 | 0.18 |
| Isoleucine (Ile) | 2.09  1.96-2.25 | 2.06  1.85-2.18 | 2.17  2.11-2.30 | 2.16^#^  2.05-2.21 | **Treatment** | 6.33 | 0.02 |
|  |  |  |  |  | **Time** | 11.73 | 0.002 |
|  |  |  |  |  | **Treatment X time** | 0.41 | 0.53 |
| Leucine (Leu) | 2.31  2.08-2.45 | 2.30  2.14-2.43 | 2.41  2.30-2.65 | 2.40^#^  2.32-2.54 | **Treatment** | 1.39 | 0.25 |
|  |  |  |  |  | **Time** | 15.98 | 0.0004 |
|  |  |  |  |  | **Treatment X time** | 0.53 | 0.47 |
| Lysine (Lys) | 2.53  2.31-2.71 | 2.41  2.22-2.46 | 2.44  2.36-2.82 | 2.49  2.32-2.79 | **Treatment** | 2.46 | 0.13 |
|  |  |  |  |  | **Time** | 1.08 | 0.31 |
|  |  |  |  |  | **Treatment X time** | 3.57 | 0.07 |
| Methionine (Met) | 1.92  1.85-2.16 | 1.77*  1.65-1.98 | 2.11  1.94-2.37 | 2.02^##^  1.71-2.29 | **Treatment** | 10.85 | 0.002 |
|  |  |  |  |  | **Time** | 17.54 | 0.0002 |
|  |  |  |  |  | **Treatment X time** | 1.19 | 0.28 |
| Ornithine (Orn) | 2.05  1.86-2.31 | 1.93  1.82-2.22 | 2.03  1.93-2.18 | 1.97  1.67-2.13 | **Treatment** | 10.25 | 0.003 |
|  |  |  |  |  | **Time** | 0.22 | 0.64 |
|  |  |  |  |  | **Treatment X time** | 0.001 | 0.98 |
| Phenylalanine (Phe) | 1.97  1.91-2.07 | 1.91*  1.80-1.95 | 2.06^@@^  2.00-2.29 | 2.11^####^  2.01-2.18 | **Treatment** | 4.25 | 0.05 |
|  |  |  |  |  | **Time** | 54.38 | <0.0001 |
|  |  |  |  |  | **Treatment X time** | 3.74 | 0.06 |
| Proline (Pro) | 2.07  1.99-2.35 | 1.81**  1.76-1.93 | 2.19  1.91-2.53 | 2.15^##^  1.76-2.33 | **Treatment** | 13.98 | 0.0007 |
|  |  |  |  |  | **Time** | 16.18 | 0.0003 |
|  |  |  |  |  | **Treatment X time** | 2.73 | 0.11 |
| Serine (Ser) | 2.16  1.98-2.30 | 1.86***  1.75-2.03 | 2.21  2.08-2.50 | 2.13^###^  2.02-2.43 | **Treatment** | 17.36 | 0.0002 |
|  |  |  |  |  | **Time** | 19.52 | 0.0001 |
|  |  |  |  |  | **Treatment X time** | 5.21 | 0.03 |
| Threonine (Thr) | 2.23  2.11-2.32 | 2.08*  2.01-2.30 | 2.30  2.24-2.48 | 2.36^####^  2.15-2.45 | **Treatment** | 3.67 | 0.06 |
|  |  |  |  |  | **Time** | 34.66 | <0.0001 |
|  |  |  |  |  | **Treatment X time** | 8.88 | 0.0056 |
| Tryptophan (Trp) | 2.06  1.91-2.23 | 2.02  1.91-2.11 | 2.17^@^  2.08-2.29 | 2.12  1.87-2.27 | **Treatment** | 4.18 | 0.049 |
|  |  |  |  |  | **Time** | 11.60 | 0.002 |
|  |  |  |  |  | **Treatment X time** | 0.58 | 0.45 |
| Tyrosine (Tyr) | 1.85  1.74-2.05 | 1.69**  1.52-1.85 | 2.04^@@^  1.96-2.29 | 1.94*^####^  1.75-2.15 | **Treatment** | 21.46 | <0.0001 |
|  |  |  |  |  | **Time** | 36.56 | <0.0001 |
|  |  |  |  |  | **Treatment X time** | 0.57 | 0.46 |
| Valine (Val) | 2.40  2.36-2.56 | 2.32*  2.17-2.53 | 2.51  2.40-2.64 | 2.43  2.37-2.52 | **Treatment** | 14.68 | 0.0006 |
|  |  |  |  |  | **Time** | 10.19 | 0.003 |
|  |  |  |  |  | **Treatment X time** | 0.12 | 0.73 |
| **Biogenic Amines** | | | | | | | |
| Acetylornithine (Ac-Orn) | 0.90  0.79-1.03 | 0.82***  0.68-0.94 | 0.90  0.82-1.00 | 0.72****  0.61-0.80 | **Treatment** | 60.27 | <0.0001 |
|  |  |  |  |  | **Time** | 4.04 | 0.05 |
|  |  |  |  |  | **Treatment X time** | 1.54 | 0.22 |
| Asymmetric dimethylarginine (ADMA) | -0.18  -0.35-0.05 | -0.14  -0.24-0.09 | -0.32^@^  -0.42-(-0.20) | -0.20*  -0.46-0.11 | **Treatment** | 6.23 | 0.02 |
|  |  |  |  |  | **Time** | 7.74 | 0.009 |
|  |  |  |  |  | **Treatment X time** | 1.81 | 0.19 |
| Alpha-Aminoadipic acid  (alpha-AAA) | 0.90  0.72-1.02 | 0.75*  0.44-0.93 | 0.87  0.73-1.04 | 0.88  0.66-0.99 | **Treatment** | 6.78 | 0.01 |
|  |  |  |  |  | **Time** | 1.84 | 0.19 |
|  |  |  |  |  | **Treatment X time** | 4.70 | 0.04 |
| Carnosine | 0.86  0.67-1.00 | 0.85  0.70-1.03 | 0.74  0.63-0.89 | 0.79  0.52-0.95 | **Treatment** | 0.03 | 0.87 |
|  |  |  |  |  | **Time** | 8.48 | 0.007 |
|  |  |  |  |  | **Treatment X time** | 0.39 | 0.54 |
| Creatinine | 1.20  1.18-1.22 | 1.21  1.18-1.24 | 1.19  1.18-1.21 | 1.21  1.18-1.26 | **Treatment** | 5.50 | 0.02 |
|  |  |  |  |  | **Time** | 1.49 | 0.23 |
|  |  |  |  |  | **Treatment X time** | 0.33 | 0.57 |
| Histamine | 0.17  -0.17-0.31 | 0.11  -0.09-0.22 | 0.09  -0.12-0.23 | 0.12  -0.002-0.37 | **Treatment** | 0.37 | 0.55 |
|  |  |  |  |  | **Time** | 0.01 | 0.90 |
|  |  |  |  |  | **Treatment X time** | 1.62 | 0.21 |
| Putrescine | 0.26  0.06-0.37 | -0.13**  -0.85-0.55 | 0.08  -0.05-0.31 | 0.42***^####^  0.26-0.51 | **Treatment** | 0.012 | 0.91 |
|  |  |  |  |  | **Time** | 11.09 | 0.002 |
|  |  |  |  |  | **Treatment X time** | 39.60 | <0.0001 |
| Symmetric dimethylarginine (SDMA) | -0.26  -0.35-(-0.14) | -0.17  -0.33-0.00 | -0.32  -0.37-(-0.23) | -0.19**  -0.32-(-0.09) | **Treatment** | 19.88 | 0.0001 |
|  |  |  |  |  | **Time** | 3.65 | 0.07 |
|  |  |  |  |  | **Treatment X time** | 0.14 | 0.71 |
| Serotonin (5-HT) | 1.32  0.44-1.56 | 1.35  0.85-1.45 | 1.35  1.08-1.51 | 1.05*  0.57-1.35 | **Treatment** | 4.69 | 0.04 |
|  |  |  |  |  | **Time** | 2.02 | 0.17 |
|  |  |  |  |  | **Treatment X time** | 5.03 | 0.03 |
| Spermidine | 0.69  0.57-1.02 | 0.65  0.60-0.82 | 0.69  0.58-0.79 | 0.65  0.58-0.87 | **Treatment** | 0.20 | 0.66 |
|  |  |  |  |  | **Time** | 0.001 | 0.97 |
|  |  |  |  |  | **Treatment X time** | 0.74 | 0.40 |
| Spermine | 0.08  0.03-0.34 | 0.08  0.02-0.19 | 0.05  -0.07-0.21 | 0.02  -0.09-0.16 | **Treatment** | 0.94 | 0.34 |
|  |  |  |  |  | **Time** | 4.12 | 0.05 |
|  |  |  |  |  | **Treatment X time** | 0.08 | 0.78 |
| Trans-4-Hydroxyproline (t4-OH-Pro) | 1.27  1.17-1.43 | 1.16  1.08-1.29 | 1.18  0.97-1.26 | 1.33  1.10-1.50 | **Treatment** | 0.22 | 0.64 |
|  |  |  |  |  | **Time** | 0.21 | 0.65 |
|  |  |  |  |  | **Treatment X time** | 10.30 | 0.003 |
| Taurine | 2.59  2.53-2.65 | 2.59  2.54-2.68 | 2.57  2.50-2.63 | 2.59  2.53-2.62 | **Treatment** | 0.70 | 0.41 |
|  |  |  |  |  | **Time** | 3.06 | 0.09 |
|  |  |  |  |  | **Treatment X time** | 0.005 | 0.94 |
| Kynurenine | -0.003  -0.18-0.10 | -0.06  -0.42-0.18 | 0.10  -0.09-0.20 | 0.48****^####^  0.26-0.75 | **Treatment** | 13.31 | 0.00096 |
|  |  |  |  |  | **Time** | 50.84 | <0.0001 |
|  |  |  |  |  | **Treatment X time** | 31.77 | <0.0001 |
| Methionine sulfoxide (Met-SO) | -0.04  -0.26-0.24 | -0.35  -1.03-0.59 | 0.09  -0.87-0.43 | -0.27  -1.24-0.13 | **Treatment** | 6.84 | 0.014 |
|  |  |  |  |  | **Time** | 0.41 | 0.53 |
|  |  |  |  |  | **Treatment X time** | 0.40 | 0.53 |
| **Glycerophospholipids** | | | | | | | |
| ***Lysophosphatidylcholine acyls*** | | | | | | | |
| lysoPC a C14:0 | 1.11  0.98-1.17 | 1.22  0.90-1.31 | 1.09  0.92-1.19 | 1.02  0.98-1.05 | **Treatment** | 0.08 | 0.79 |
|  |  |  |  |  | **Time** | 2.82 | 0.10 |
|  |  |  |  |  | **Treatment X time** | 2.53 | 0.12 |
| lysoPC a C16:0 | 2.72  2.60-2.76 | 2.73  2.60-2.81 | 2.71  2.55-2.78 | 2.67  2.53-2.77 | **Treatment** | 0.11 | 0.75 |
|  |  |  |  |  | **Time** | 2.41 | 0.13 |
|  |  |  |  |  | **Treatment X time** | 0.75 | 0.39 |
| lysoPC a C16:1 | 1.12  1.03-1.33 | 1.13  1.02-1.26 | 1.15  0.99-1.29 | 0.91****^####^  0.89-1.00 | **Treatment** | 17.92 | 0.0001 |
|  |  |  |  |  | **Time** | 11.97 | 0.002 |
|  |  |  |  |  | **Treatment X time** | 18.16 | 0.0002 |
| lysoPC a C17:0 | 0.78  0.65-0.95 | 0.84  0.71-0.96 | 0.85  0.74-0.94 | 0.67**^#^  0.56-0.85 | **Treatment** | 3.79 | 0.06 |
|  |  |  |  |  | **Time** | 2.22 | 0.15 |
|  |  |  |  |  | **Treatment X time** | 8.76 | 0.006 |
| lysoPC a C18:0 | 2.19  2.07-2.41 | 2.23  2.08-2.29 | 2.22  2.04-2.35 | 2.31  2.06-2.45 | **Treatment** | 1.09 | 0.30 |
|  |  |  |  |  | **Time** | 1.21 | 0.28 |
|  |  |  |  |  | **Treatment X time** | 1.73 | 0.29 |
| lysoPC a C18:1 | 1.93  1.82-2.10 | 1.95  1.83-2.09 | 1.95  1.84-2.06 | 1.77***^##^  1.61-1.90 | **Treatment** | 8.02 | 0.008 |
|  |  |  |  |  | **Time** | 9.43 | 0.004 |
|  |  |  |  |  | **Treatment X time** | 9.73 | 0.004 |
| lysoPC a C18:2 | 2.39  2.26-2.46 | 2.36  2.18-2.44 | 2.36  2.33-2.50 | 2.16****^####^  1.95-2.28 | **Treatment** | 30.05 | <0.0001 |
|  |  |  |  |  | **Time** | 14.33 | 0.0006 |
|  |  |  |  |  | **Treatment X time** | 13.50 | 0.0009 |
| lysoPC a C20:3 | 1.13  0.93-1.33 | 1.11  0.90-1.27 | 1.11  0.93-1.28 | 0.59****^####^  0.34-0.93 | **Treatment** | 37.90 | <0.0001 |
|  |  |  |  |  | **Time** | 28.20 | <0.0001 |
|  |  |  |  |  | **Treatment X time** | 30.95 | <0.0001 |
| lysoPC a C20:4 | 1.73  1.54-1.91 | 1.75  1.56-1.89 | 1.68  1.56-1.75 | 1.35****^####^  1.16-1.55 | **Treatment** | 14.20 | 0.0007 |
|  |  |  |  |  | **Time** | 27.24 | <0.0001 |
|  |  |  |  |  | **Treatment X time** | 19.72 | 0.0001 |
| lysoPC a C24:0 | -0.07  -0.40-0.17 | 0.05  -0.25-0.29 | -0.07  -0.47-0.19 | 0.03  -0.17-0.48 | **Treatment** | 5.26 | 0.03 |
|  |  |  |  |  | **Time** | 0.28 | 0.60 |
|  |  |  |  |  | **Treatment X time** | 0.13 | 0.72 |
| lysoPC a C26:0 | 0.06  -0.29-0.35 | 0.29  -0.22-0.55 | -0.04  -0.38-0.52 | -0.05  -0.46-0.50 | **Treatment** | 0.12 | 0.73 |
|  |  |  |  |  | **Time** | 1.39 | 0.25 |
|  |  |  |  |  | **Treatment X time** | 1.70 | 0.20 |
| lysoPC a C26:1 | -0.39  -0.74-(-0.14) | -0.20  -0.68-0.03 | -0.53  -0.82-(-0.10) | -0.54  -0.72-(-0.02) | **Treatment** | 1.21 | 0.28 |
|  |  |  |  |  | **Time** | 1.94 | 0.17 |
|  |  |  |  |  | **Treatment X time** | 0.99 | 0.33 |
| lysoPC a C28:0 | -0.26  -0.61-0.03 | -0.10  -0.48-0.15 | -0.36  -0.78-0.12 | -0.48  -0.68-0.16 | **Treatment** | 0.15 | 0.70 |
|  |  |  |  |  | **Time** | 1.43 | 0.24 |
|  |  |  |  |  | **Treatment X time** | 0.87 | 0.36 |
| lysoPC a C28:1 | -0.39  -0.80-(-0.12) | -0.17  -0.63-0.05 | -0.48  -1.00-0.04 | -0.55  -0.71-0.05 | **Treatment** | 0.92 | 0.35 |
|  |  |  |  |  | **Time** | 1.76 | 0.19 |
|  |  |  |  |  | **Treatment X time** | 1.01 | 0.32 |
| ***Phosphatidylcholine diacyls*** | | | | | | | |
| PC aa C24:0 | -0.22  -0.49-0.10 | -0.07  -0.44-0.20 | -0.31  -0.66-0.10 | -0.30  -0.62-0.19 | **Treatment** | 0.62 | 0.44 |
|  |  |  |  |  | **Time** | 1.20 | 0.28 |
|  |  |  |  |  | **Treatment X time** | 0.43 | 0.52 |
| PC aa C26:0 | 0.57  0.20-0.90 | 0.69  0.25-0.94 | 0.48  0.08-0.95 | 0.38  0.19-0.96 | **Treatment** | 0.36 | 0.55 |
|  |  |  |  |  | **Time** | 0.77 | 0.39 |
|  |  |  |  |  | **Treatment X time** | 0.51 | 0.48 |
| PC aa C28:1 | -0.08  -0.51-0.22 | 0.09  -0.40-0.34 | -0.12  -0.39-0.35 | -0.17  -0.35-0.35 | **Treatment** | 0.39 | 0.54 |
|  |  |  |  |  | **Time** | 0.15 | 0.70 |
|  |  |  |  |  | **Treatment X time** | 0.74 | 0.39 |
| PC aa C30:0 | 0.11  -0.10-0.44 | 0.14  -0.05-0.43 | 0.18  -0.08-0.68 | 0.10  -0.03-0.38 | **Treatment** | 0.80 | 0.38 |
|  |  |  |  |  | **Time** | 0.37 | 0.55 |
|  |  |  |  |  | **Treatment X time** | 1.43 | 0.24 |
| PC aa C32:0 | 1.03  0.83-1.22 | 1.04  0.77-1.23 | 1.12  0.81-1.46 | 0.99  0.87-1.21 | **Treatment** | 0.51 | 0.48 |
|  |  |  |  |  | **Time** | 0.76 | 0.39 |
|  |  |  |  |  | **Treatment X time** | 0.48 | 0.49 |
| PC aa C32:1 | 0.70  0.43-0.88 | 0.62  0.45-0.88 | 0.79  0.32-1.15 | 0.55  0.45-0.80 | **Treatment** | 2.63 | 0.11 |
|  |  |  |  |  | **Time** | 0.01 | 0.92 |
|  |  |  |  |  | **Treatment X time** | 1.13 | 0.30 |
| PC aa C32:2 | -0.18  -0.32-(-0.03) | -0.18  -0.28-(-0.02) | -0.20  -0.39-0.009 | -0.29  -0.43-(-0.16) | **Treatment** | 1.02 | 0.32 |
|  |  |  |  |  | **Time** | 4.32 | 0.05 |
|  |  |  |  |  | **Treatment X time** | 2.38 | 0.13 |
| PC aa C32:3 | -0.92  -1.22-(-0.80) | -0.94  -1.07-(-0.78) | -1.04  -1.22-(-0.77) | -0.80*  -1.03-(-0.63) | **Treatment** | 7.00 | 0.01 |
|  |  |  |  |  | **Time** | 0.88 | 0.36 |
|  |  |  |  |  | **Treatment X time** | 2.22 | 0.15 |
| PC aa C34:1 | 1.91  1.78-2.00 | 1.93  1.75-2.01 | 1.86  1.61-2.04 | 1.97*  1.88-2.06 | **Treatment** | 5.37 | 0.03 |
|  |  |  |  |  | **Time** | 0.13 | 0.72 |
|  |  |  |  |  | **Treatment X time** | 2.79 | 0.10 |
| PC aa C34:2 | 2.46  2.34-2.60 | 2.44  2.34-2.50 | 2.46  2.31-2.56 | 2.55*^#^  2.48-2.61 | **Treatment** | 1.70 | 0.20 |
|  |  |  |  |  | **Time** | 4.36 | 0.05 |
|  |  |  |  |  | **Treatment X time** | 8.39 | 0.007 |
| PC aa C34:3 | 0.95  0.85-1.09 | 0.95  0.83-1.04 | 0.90  0.75-1.05 | 0.92  0.81-1.03 | **Treatment** | 0.61 | 0.44 |
|  |  |  |  |  | **Time** | 2.76 | 0.11 |
|  |  |  |  |  | **Treatment X time** | 1.82 | 0.19 |
| PC aa C34:4 | -0.57  -0.69-(-0.45) | -0.52  -0.74-(-0.44) | -0.61  -0.74-(-0.44) | -0.71^#^  -0.89-(-0.62) | **Treatment** | 3.41 | 0.07 |
|  |  |  |  |  | **Time** | 11.10 | 0.002 |
|  |  |  |  |  | **Treatment X time** | 2.60 | 0.12 |
| PC aa C36:0 | 0.32  0.22-0.40 | 0.30  0.15-0.40 | 0.35  0.21-0.47 | 0.31  0.12-0.42 | **Treatment** | 3.28 | 0.08 |
|  |  |  |  |  | **Time** | 0.03 | 0.87 |
|  |  |  |  |  | **Treatment X time** | 0.61 | 0.44 |
| PC aa C36:1 | 1.11  0.99-1.31 | 1.14  0.98-1.20 | 1.12  0.83-1.20 | 1.39****^####^  1.27-1.52 | **Treatment** | 21.46 | <0.0001 |
|  |  |  |  |  | **Time** | 13.46 | 0.0009 |
|  |  |  |  |  | **Treatment X time** | 26.31 | <0.0001 |
| PC aa C36:2 | 2.12  2.05-2.34 | 2.09  2.00-2.23 | 2.21  2.05-2.29 | 2.26^##^  2.19-2.43 | **Treatment** | 0.09 | 0.77 |
|  |  |  |  |  | **Time** | 13.43 | 0.0009 |
|  |  |  |  |  | **Treatment X time** | 5.91 | 0.02 |
| PC aa C36:3 | 1.71  1.60-1.82 | 1.70  1.54-1.77 | 1.78  1.62-1.85 | 1.61*  1.54-1.81 | **Treatment** | 8.91 | 0.005 |
|  |  |  |  |  | **Time** | 0.03 | 0.86 |
|  |  |  |  |  | **Treatment X time** | 2.27 | 0.14 |
| PC aa C36:4 | 1.92  1.82-2.01 | 1.94  1.77-1.99 | 1.89  1.77-1.98 | 1.83  1.76-2.00 | **Treatment** | 0.92 | 0.34 |
|  |  |  |  |  | **Time** | 1.45 | 0.24 |
|  |  |  |  |  | **Treatment X time** | 0.13 | 0.72 |
| PC aa C36:5 | 0.61  0.51-0.76 | 0.59  0.41-0.67 | 0.57  0.49-0.85 | 0.53  0.44-0.67 | **Treatment** | 2.92 | 0.10 |
|  |  |  |  |  | **Time** | 0.01 | 0.91 |
|  |  |  |  |  | **Treatment X time** | 0.02 | 0.90 |
| PC aa C36:6 | -0.69  -0.82-(-0.51) | -0.64  -0.74-(-0.47) | -0.70  -0.95-(-0.48) | -0.68  -0.87-(-0.61) | **Treatment** | 0.58 | 0.45 |
|  |  |  |  |  | **Time** | 1.41 | 0.24 |
|  |  |  |  |  | **Treatment X time** | 1.08 | 0.31 |
| PC aa C38:0 | 0.05  -0.03-0.14 | 0.05  -0.03-0.16 | 0.07  -0.10-0.25 | 0.05  -0.07-0.18 | **Treatment** | 0.12 | 0.73 |
|  |  |  |  |  | **Time** | 0.11 | 0.74 |
|  |  |  |  |  | **Treatment X time** | 0.29 | 0.59 |
| PC aa C38:1 | -0.05  -0.18-0.06 | -0.05  -0.12-0.01 | -0.02  -0.35-0.10 | 0.01  -0.18-0.22 | **Treatment** | 2.04 | 0.16 |
|  |  |  |  |  | **Time** | 0.93 | 0.34 |
|  |  |  |  |  | **Treatment X time** | 0.89 | 0.35 |
| PC aa C38:3 | 1.20  1.02-1.37 | 1.15  1.02-1.29 | 1.24  0.95-1.31 | 1.12  1.02-1.31 | **Treatment** | 0.56 | 0.46 |
|  |  |  |  |  | **Time** | 0.14 | 0.71 |
|  |  |  |  |  | **Treatment X time** | <0.0001 | 1.00 |
| PC aa C38:4 | 1.63  1.46-1.81 | 1.64  1.47-1.71 | 1.60  1.31-1.70 | 1.68*  1.59-1.92 | **Treatment** | 3.81 | 0.06 |
|  |  |  |  |  | **Time** | 0.35 | 0.56 |
|  |  |  |  |  | **Treatment X time** | 5.58 | 0.02 |
| PC aa C38:5 | 1.20  1.07-1.35 | 1.25  1.09-1.33 | 1.22  1.03-1.33 | 1.19  1.05-1.34 | **Treatment** | 0.01 | 0.91 |
|  |  |  |  |  | **Time** | 0.48 | 0.49 |
|  |  |  |  |  | **Treatment X time** | 0.10 | 0.75 |
| PC aa C38:6 | 1.86  1.73-1.95 | 1.88  1.76-1.95 | 1.82  1.40-1.92 | 2.05****^##^  1.95-2.16 | **Treatment** | 19.7 | 0.0001 |
|  |  |  |  |  | **Time** | 2.3 | 0.1 |
|  |  |  |  |  | **Treatment X time** | 13.3 | 0.001 |
| PC aa C40:2 | -0.38  -0.48-(-0.24) | -0.35  -0.45-(-0.31) | -0.39  -0.64-(-0.21) | -0.49  -0.63-(-0.34) | **Treatment** | 2.15 | 0.15 |
|  |  |  |  |  | **Time** | 5.91 | 0.02 |
|  |  |  |  |  | **Treatment X time** | 1.97 | 0.17 |
| PC aa C40:3 | -0.25  -0.35-(-0.08) | -0.21  -0.37-(-0.17) | -0.27  -0.56-(-0.06) | -0.41^#^  -0.66-(-0.27) | **Treatment** | 2.70 | 0.11 |
|  |  |  |  |  | **Time** | 10.30 | 0.003 |
|  |  |  |  |  | **Treatment X time** | 2.46 | 0.13 |
| PC aa C40:4 | 0.28  0.16-0.47 | 0.26  0.18-0.35 | 0.27  -0.06-0.41 | 0.29  0.17-0.38 | **Treatment** | 0.009 | 0.93 |
|  |  |  |  |  | **Time** | 0.74 | 0.40 |
|  |  |  |  |  | **Treatment X time** | 1.28 | 0.27 |
| PC aa C40:5 | 0.39  0.26-0.61 | 0.37  0.29-0.53 | 0.43  0.14-0.55 | 0.56*^#^  0.39-0.70 | **Treatment** | 4.79 | 0.04 |
|  |  |  |  |  | **Time** | 4.44 | 0.04 |
|  |  |  |  |  | **Treatment X time** | 4.83 | 0.04 |
| PC aa C40:6 | 1.26  1.17-1.49 | 1.28  1.18-1.41 | 1.31  0.81-1.40 | 1.69****^####^  1.61-1.85 | **Treatment** | 29.57 | <0.0001 |
|  |  |  |  |  | **Time** | 19.17 | 0.0001 |
|  |  |  |  |  | **Treatment X time** | 27.55 | <0.0001 |
| PC aa C42:0 | -0.91  -1.08-(-0.84) | -0.88  -1.16-(-0.71) | -0.93  -1.15-(-0.73) | -0.98  -1.02-(-0.75) | **Treatment** | 0.12 | 0.73 |
|  |  |  |  |  | **Time** | 0.01 | 0.94 |
|  |  |  |  |  | **Treatment X time** | 0.06 | 0.81 |
| PC aa C42:1 | -0.92  -1.03-(-0.81) | -0.94  -1.08-(-0.85) | -0.85  -1.14-(-0.76) | -0.95  -1.07-(-0.77) | **Treatment** | 0.73 | 0.40 |
|  |  |  |  |  | **Time** | 0.74 | 0.40 |
|  |  |  |  |  | **Treatment X time** | 0.16 | 0.69 |
| PC aa C42:2 | -0.63  -0.76-(-0.53) | -0.58  -0.73-(-0.50) | -0.62  -0.81-(-0.44) | -0.67  -0.85-(-0.50) | **Treatment** | 0.03 | 0.87 |
|  |  |  |  |  | **Time** | 1.94 | 0.17 |
|  |  |  |  |  | **Treatment X time** | 0.93 | 0.34 |
| PC aa C42:4 | -0.72  -0.81-(-0.55) | -0.72  -0.85-(-0.58) | -0.72  -0.96-(-0.55) | -0.63*  -0.77-(-0.46) | **Treatment** | 2.60 | 0.12 |
|  |  |  |  |  | **Time** | 0.42 | 0.52 |
|  |  |  |  |  | **Treatment X time** | 5.43 | 0.03 |
| PC aa C42:5 | -0.59  -0.74-(-0.52) | -0.62  -0.68-(-0.50) | -0.47  -0.56-(-0.37) | -0.57  -0.74-(-0.44) | **Treatment** | 1.92 | 0.18 |
|  |  |  |  |  | **Time** | 0.09 | 0.76 |
|  |  |  |  |  | **Treatment X time** | 1.70 | 0.20 |
| PC aa C42:6 | -0.2  -0.21-0.02 | -0.08  -0.18-(-0.01) | -0.09  -0.55-0.03 | -0.06  -0.21-0.01 | **Treatment** | 1.22 | 0.28 |
|  |  |  |  |  | **Time** | 0.47 | 0.50 |
|  |  |  |  |  | **Treatment X time** | 0.70 | 0.41 |
| ***Phosphatidylcholine acyl-alkyls*** | | | | | | | |
| PC ae C30:1 | -0.25  -0.73-0.12 | -0.10  -0.61-0.17 | -0.31  -0.74-0.17 | -0.35  -0.83-0.16 | **Treatment** | 0.14 | 0.71 |
|  |  |  |  |  | **Time** | 1.02 | 0.32 |
|  |  |  |  |  | **Treatment X time** | 0.80 | 0.38 |
| PC ae C30:2 | -0.84  -1.19-(-0.53) | -0.70  -1.13-(-0.51) | -0.92  -1.23-(-0.54) | -0.90  -1.11-(-0.44) | **Treatment** | 0.74 | 0.40 |
|  |  |  |  |  | **Time** | 0.40 | 0.53 |
|  |  |  |  |  | **Treatment X time** | 0.009 | 0.92 |
| PC ae C32:1 | -0.33  -0.57-(-0.11) | -0.28  -0.56-(-0.10) | -0.32  -0.43-(-0.01) | -0.38  -0.50-0.05 | **Treatment** | 0.0005 | 0.98 |
|  |  |  |  |  | **Time** | 0.20 | 0.66 |
|  |  |  |  |  | **Treatment X time** | 0.17 | 0.68 |
| PC ae C32:2 | -0.64  -0.88-(-0.45) | -0.55  -0.96-(-0.37) | -0.74  -0.84-(-0.37) | -0.83  -0.93-(-0.39) | **Treatment** | 0.11 | 0.74 |
|  |  |  |  |  | **Time** | 1.43 | 0.24 |
|  |  |  |  |  | **Treatment X time** | 0.34 | 0.56 |
| PC ae C34:0 | -0.49  -0.67-(-0.42) | -0.48  -0.53-(-0.34) | -0.50  -0.55-(-0.25) | -0.56  -0.63-(-0.36) | **Treatment** | 0.08 | 0.78 |
|  |  |  |  |  | **Time** | 0.42 | 0.52 |
|  |  |  |  |  | **Treatment X time** | 5.46 | 0.03 |
| PC ae C34:1 | 0.29  0.17-0.36 | 0.30  0.13-0.37 | 0.29  0.14-0.38 | 0.27  0.21-0.39 | **Treatment** | 0.17 | 0.69 |
|  |  |  |  |  | **Time** | 0.45 | 0.51 |
|  |  |  |  |  | **Treatment X time** | 0.18 | 0.68 |
| PC ae C34:2 | 0.35  0.25-0.46 | 0.32  0.18-0.46 | 0.34  0.23-0.44 | 0.34  0.26-0.43 | **Treatment** | 0.51 | 0.48 |
|  |  |  |  |  | **Time** | 0.01 | 0.91 |
|  |  |  |  |  | **Treatment X time** | 0.60 | 0.44 |
| PC ae C34:3 | -0.24  -0.35-(-0.04) | -0.21  -0.39-(-0.11) | -0.18  -0.37-(-0.08) | -0.32  -0.40-(-0.09) | **Treatment** | 1.96 | 0.17 |
|  |  |  |  |  | **Time** | 0.58 | 0.45 |
|  |  |  |  |  | **Treatment X time** | 2.21 | 0.15 |
| PC ae C36:0 | -0.39  -0.43-(-0.32) | -0.35  -0.52-(-0.25) | -0.40  -0.56-(-0.20) | -0.44  -0.51-(-0.30) | **Treatment** | 0.007 | 0.93 |
|  |  |  |  |  | **Time** | 1.50 | 0.23 |
|  |  |  |  |  | **Treatment X time** | 0.45 | 0.51 |
| PC ae C36:1 | 0.38  0.26-0.46 | 0.42  0.28-0.51 | 0.43  0.20-0.56 | 0.41  0.34-0.54 | **Treatment** | 0.86 | 0.36 |
|  |  |  |  |  | **Time** | 0.31 | 0.58 |
|  |  |  |  |  | **Treatment X time** | 0.07 | 0.79 |
| PC ae C36:2 | 0.76  0.66-0.89 | 0.76  0.62-0.88 | 0.85  0.73-0.95 | 0.74*  0.61-0.85 | **Treatment** | 7.53 | 0.01 |
|  |  |  |  |  | **Time** | 0.78 | 0.38 |
|  |  |  |  |  | **Treatment X time** | 2.07 | 0.16 |
| PC ae C36:3 | 0.13  0.04-0.24 | 0.15  -0.02-0.20 | 0.13  0.00-0.25 | 0.13  0.05-0.20 | **Treatment** | 0.02 | 0.89 |
|  |  |  |  |  | **Time** | 0.07 | 0.79 |
|  |  |  |  |  | **Treatment X time** | 0.04 | 0.84 |
| PC ae C36:4 | 0.18  0.08-0.35 | 0.24  0.08-0.47 | 0.25  0.18-0.36 | 0.16  0.08-0.31 | **Treatment** | 2.43 | 0.13 |
|  |  |  |  |  | **Time** | 0.30 | 0.59 |
|  |  |  |  |  | **Treatment X time** | 3.82 | 0.06 |
| PC ae C36:5 | -0.05  -0.06-0.18 | 0.02  -0.20-0.15 | 0.06  -0.14-0.18 | 0.11  -0.02-0.17 | **Treatment** | 0.14 | 0.71 |
|  |  |  |  |  | **Time** | 4.36 | 0.04 |
|  |  |  |  |  | **Treatment X time** | 0.80 | 0.38 |
| PC ae C38:0 | 0.25  0.15-0.37 | 0.26  0.12-0.36 | 0.27  -0.06-0.48 | 0.30  0.19-0.39 | **Treatment** | 0.71 | 0.40 |
|  |  |  |  |  | **Time** | 0.06 | 0.81 |
|  |  |  |  |  | **Treatment X time** | 0.56 | 0.46 |
| PC ae C38:1 | -0.08  -0.16-0.03 | -0.10  -0.22-0.09 | -0.04  -0.26-0.16 | -0.15  -0.23-0.12 | **Treatment** | 1.78 | 0.19 |
|  |  |  |  |  | **Time** | 0.36 | 0.55 |
|  |  |  |  |  | **Treatment X time** | 1.37 | 0.25 |
| PC ae C38:2 | 0.68  0.54-0.82 | 0.66  0.43-0.85 | 0.80  0.60-0.91 | 0.45****^#^  0.32-0.68 | **Treatment** | 26.44 | <0.0001 |
|  |  |  |  |  | **Time** | 0.80 | 0.38 |
|  |  |  |  |  | **Treatment X time** | 16.31 | 0.0003 |
| PC ae C38:3 | 0.12  -0.02-0.23 | 0.11  -0.02-0.21 | 0.18  0.04-0.29 | -0.03**  -0.09-0.21 | **Treatment** | 6.32 | 0.02 |
|  |  |  |  |  | **Time** | 0.46 | 0.50 |
|  |  |  |  |  | **Treatment X time** | 5.60 | 0.02 |
| PC ae C38:4 | 0.33  0.23-0.48 | 0.37  0.17-0.50 | 0.37  0.24-0.45 | 0.25  0.14-0.39 | **Treatment** | 0.71 | 0.40 |
|  |  |  |  |  | **Time** | 0.80 | 0.38 |
|  |  |  |  |  | **Treatment X time** | 1.36 | 0.25 |
| PC ae C38:5 | 0.12  0.06-0.34 | 0.19  0.05-0.38 | 0.23  0.18-0.32 | 0.07***^#^  -0.07-0.16 | **Treatment** | 8.02 | 0.008 |
|  |  |  |  |  | **Time** | 1.78 | 0.19 |
|  |  |  |  |  | **Treatment X time** | 12.45 | 0.001 |
| PC ae C38:6 | 0.03  -0.07-0.15 | 0.06  -0.09-0.41 | 0.09  -0.10-0.19 | 0.14  0.08-0.20 | **Treatment** | 3.61 | 0.07 |
|  |  |  |  |  | **Time** | 2.45 | 0.13 |
|  |  |  |  |  | **Treatment X time** | 0.14 | 0.71 |
| PC ae C40:1 | 0.29  0.21-0.40 | 0.31  0.16-0.48 | 0.29  -0.02-0.41 | 0.20  0.06-0.33 | **Treatment** | 0.28 | 0.60 |
|  |  |  |  |  | **Time** | 4.34 | 0.05 |
|  |  |  |  |  | **Treatment X time** | 1.44 | 0.24 |
| PC ae C40:2 | -0.33  -0.40-(-0.23) | -0.26  -0.39-(-0.20) | -0.30  -0.57-(-0.17) | -0.35  -0.53-(-0.20) | **Treatment** | 0.08 | 0.78 |
|  |  |  |  |  | **Time** | 0.93 | 0.34 |
|  |  |  |  |  | **Treatment X time** | 1.81 | 0.19 |
| PC ae C40:3 | -0.29  -0.43-(-0.12) | -0.29  -0.39-(-0.20) | -0.24  -0.44-(-0.15) | -0.42  -0.48-(-0.20) | **Treatment** | 2.62 | 0.12 |
|  |  |  |  |  | **Time** | 1.45 | 0.24 |
|  |  |  |  |  | **Treatment X time** | 2.08 | 0.16 |
| PC ae C40:4 | 0.07  -0.06-0.26) | 0.12  -0.11-0.28 | 0.09  -0.03-0.23 | -0.02  -0.15-0.11 | **Treatment** | 1.35 | 0.25 |
|  |  |  |  |  | **Time** | 1.72 | 0.20 |
|  |  |  |  |  | **Treatment X time** | 2.62 | 0.12 |
| PC ae C40:5 | -0.15  -0.17-0.00 | -0.09  -0.18-0.01 | -0.09  -0.24-0.08 | -0.05  -0.15-0.07 | **Treatment** | 0.48 | 0.49 |
|  |  |  |  |  | **Time** | 0.97 | 0.33 |
|  |  |  |  |  | **Treatment X time** | 0.04 | 0.85 |
| PC ae C40:6 | 0.14  0.02-0.29 | 0.17  0.06-0.29 | 0.15  -0.08-0.28 | 0.30**  0.18-0.34 | **Treatment** | 9.38 | 0.004 |
|  |  |  |  |  | **Time** | 3.66 | 0.06 |
|  |  |  |  |  | **Treatment X time** | 3.12 | 0.09 |
| PC ae C42:1 | -0.34  -0.51-(-0.12) | -0.28  -0.46-(-0.14) | -0.39  -0.61-(-0.16) | -0.33  -0.49-(-0.04) | **Treatment** | 2.44 | 0.13 |
|  |  |  |  |  | **Time** | 0.05 | 0.83 |
|  |  |  |  |  | **Treatment X time** | 0.22 | 0.64 |
| PC ae C42:2 | -0.54  -0.59-(-0.34) | -0.45  -0.60-(-0.39) | -0.48  -0.72-(-0.35) | -0.62  -0.82-(-0.33) | **Treatment** | 0.09 | 0.76 |
|  |  |  |  |  | **Time** | 2.33 | 0.14 |
|  |  |  |  |  | **Treatment X time** | 1.57 | 0.22 |
| PC ae C42:3 | -0.17  -0.25-(-0.02) | -0.10  -0.26-0.10 | -0.17  -0.49-0.09 | -0.29  -0.40-0.06 | **Treatment** | 0.12 | 0.73 |
|  |  |  |  |  | **Time** | 3.45 | 0.07 |
|  |  |  |  |  | **Treatment X time** | 0.66 | 0.42 |
| PC ae C44:3 | -0.89  -1.06-(-0.62) | -0.83  -1.17-(-0.61) | -0.90  -1.19-(-0.59) | -0.82  -0.98-(-0.39) | **Treatment** | 1.86 | 0.18 |
|  |  |  |  |  | **Time** | 0.69 | 0.41 |
|  |  |  |  |  | **Treatment X time** | 1.30 | 0.26 |
| PC ae C44:4 | -0.96  -1.08-(-0.80) | -0.86  -1.07-(-0.72) | -0.90  -1.15-(-0.69) | -0.84  -1.05-(-0.65) | **Treatment** | 1.65 | 0.21 |
|  |  |  |  |  | **Time** | 0.81 | 0.37 |
|  |  |  |  |  | **Treatment X time** | 0.16 | 0.70 |
| PC ae C44:6 | -1.02  -1.20-(-0.84) | -0.95  -1.11-(-0.90) | -0.95  -1.14-(-0.78) | -0.98  -1.07-(-0.79) | **Treatment** | 0.17 | 0.69 |
|  |  |  |  |  | **Time** | 1.00 | 0.33 |
|  |  |  |  |  | **Treatment X time** | 1.78 | 0.19 |
| **Sphingolipids** | | | | | | | |
| SM(OH) C14:1 | -0.54  -0.68-(-0.37) | -0.52  -0.72-(-0.30) | -0.48  -0.63-(-0.33) | -0.15****^####^  -0.30-0.03 | **Treatment** | 15.51 | 0.0004 |
|  |  |  |  |  | **Time** | 27.85 | <0.0001 |
|  |  |  |  |  | **Treatment X time** | 16.08 | 0.0003 |
| SM(OH) C16:1 | -1.19  -1.31-(-1.00) | -1.19  -1.28-(-1.11) | -1.16  -1.29-(-0.95) | -0.90**^###^  -1.14-(-0.72) | **Treatment** | 7.75 | 0.009 |
|  |  |  |  |  | **Time** | 17.06 | 0.0002 |
|  |  |  |  |  | **Treatment X time** | 9.26 | 0.005 |
| SM(OH) C22:1 | -0.06  -0.09-0.22 | 0.03  -0.08-0.16 | -0.06  -0.28-0.10 | 0.33****^####^  0.26-0.42 | **Treatment** | 42.13 | <0.0001 |
|  |  |  |  |  | **Time** | 8.09 | 0.008 |
|  |  |  |  |  | **Treatment X time** | 37.28 | <0.0001 |
| SM(OH) C22:2 | -0.34  -0.37-(-0.12) | -0.24  -0.42-(-0.09) | -0.32  -0.48-(-0.15) | -0.02****^####^  -0.08-0.15 | **Treatment** | 31.06 | <0.0001 |
|  |  |  |  |  | **Time** | 11.54 | 0.002 |
|  |  |  |  |  | **Treatment X time** | 20.15 | <0.0001 |
| SM(OH) C24:1 | -0.91  -1.04-(-0.70) | -0.99  -1.10-(-0.82) | -0.89  -1.23-(-0.67) | -0.70***^####^  -0.78-(-0.54) | **Treatment** | 3.40 | 0.07 |
|  |  |  |  |  | **Time** | 10.55 | 0.003 |
|  |  |  |  |  | **Treatment X time** | 20.39 | <0.0001 |
| SM C16:0 | 0.67  0.58-0.86 | 0.66  0.56-0.77 | 0.70  0.63-0.76 | 1.12****^####^  0.98-1.26 | **Treatment** | 47.08 | <0.0001 |
|  |  |  |  |  | **Time** | 64.74 | <0.0001 |
|  |  |  |  |  | **Treatment X time** | 56.75 | <0.0001 |
| SM C16:1 | 0.02  -0.15-0.12 | 0.04  -0.08-0.15 | 0.03  -0.29-0.12 | 0.29****^####^  0.20-0.38 | **Treatment** | 19.68 | 0.0001 |
|  |  |  |  |  | **Time** | 9.77 | 0.004 |
|  |  |  |  |  | **Treatment X time** | 18.13 | 0.0002 |
| SM C18:0 | -0.35  -0.42-(-0.25) | -0.40  -0.54-(-0.26) | -0.39  -0.49-(-0.24) | -0.11****^####^  -0.21-0.29 | **Treatment** | 15.85 | 0.0004 |
|  |  |  |  |  | **Time** | 19.79 | <0.0001 |
|  |  |  |  |  | **Treatment X time** | 28.22 | <0.0001 |
| SM C18:1 | -0.79  -0.90-(-0.71) | -0.85  -1.08-(-0.73) | -0.81  -0.95-(-0.68) | -0.55****^####^  -0.63-0.26 | **Treatment** | 15.02 | 0.0005 |
|  |  |  |  |  | **Time** | 24.67 | <0.0001 |
|  |  |  |  |  | **Treatment X time** | 29.41 | <0.0001 |
| SM C20:2 | -1.98  -2.30-(-1.68) | -1.80  -2.10-(-1.64) | -1.96  -2.40-(-1.72) | -1.55*  -2.05-(-1.09) | **Treatment** | 7.17 | 0.01 |
|  |  |  |  |  | **Time** | 1.23 | 0.28 |
|  |  |  |  |  | **Treatment X time** | 3.17 | 0.09 |
| SM C24:0 | 0.55  0.46-0.71 | 0.54  0.43-0.68 | 0.51  0.26-0.61 | 0.77****^###^  0.66-0.82 | **Treatment** | 19.66 | 0.0001 |
|  |  |  |  |  | **Time** | 2.69 | 0.11 |
|  |  |  |  |  | **Treatment X time** | 23.77 | <0.0001 |
| SM C24:1 | 0.72  0.64-0.87 | 0.74  0.61-0.83 | 0.63  0.43-0.79 | 1.16****^####^  1.11-1.31 | **Treatment** | 91.22 | <0.0001 |
|  |  |  |  |  | **Time** | 33.55 | <0.0001 |
|  |  |  |  |  | **Treatment X time** | 90.28 | <0.0001 |
| SM C26:0 | -1.82  -2.30-(-1.34) | -1.85  -2.70-(-1.52) | -1.93  -2.70-(-1.46) | -1.72  -2.10-(-1.22) | **Treatment** | 0.14 | 0.72 |
|  |  |  |  |  | **Time** | 0.11 | 0.74 |
|  |  |  |  |  | **Treatment X time** | 1.51 | 0.23 |
| SM C26:1 | -1.67  -1.96-(-1.28) | -1.39  -2.40-(-1.15) | -1.47  -2.70-(-1.39) | -1.40  -1.72-(-1.32) | **Treatment** | 1.58 | 0.22 |
|  |  |  |  |  | **Time** | 0.005 | 0.95 |
|  |  |  |  |  | **Treatment X time** | 0.49 | 0.49 |
| **Monosaccharides** | | | | | | | |
| Hexoses (H1) | 3.95  3.80-4.00 | 3.84  3.82-3.89 | 3.90  3.81-3.95 | 3.76****^##^  3.59-3.81 | **Treatment** | 35.24 | <0.0001 |
|  |  |  |  |  | **Time** | 15.61 | 0.0004 |
|  |  |  |  |  | **Treatment X time** | 3.89 | 0.057 |
| **Metabolite ratios** | | | | | | | |
| C2/C0 | -0.22  -0.33-(-0.16) | -0.21  -0.29-0.02 | -0.23  -0.36-(0.05) | -0.18  -0.41-0.24 | **Treatment** | 2.52 | 0.12 |
|  |  |  |  |  | **Time** | 0.71 | 0.40 |
|  |  |  |  |  | **Treatment X time** | 0.32 | 0.58 |
| C3/C0 | -1.62  -1.75-(-1.46) | -1.73**  -1.78-(-1.67) | -1.59  -1.64-(-1.44) | -1.58^##^  -1.68-(-1.38) | **Treatment** | 5.07 | 0.03 |
|  |  |  |  |  | **Time** | 12.55 | 0.001 |
|  |  |  |  |  | **Treatment X time** | 7.22 | 0.01 |
| C4-/C0 | -1.63  -1.75-(-1.57) | -1.70  -1.78-(-1.59) | -1.63  -1.70-(-1.46) | -1.63  -1.79-(-1.33) | **Treatment** | 0.46 | 0.50 |
|  |  |  |  |  | **Time** | 3.35 | 0.08 |
|  |  |  |  |  | **Treatment X time** | 0.55 | 0.46 |
| C5-/C0 | -2.11  -2.24-(-2.00) | -2.19  -2.27-(-2.11) | -2.06  -2.19-(-1.94) | -1.94*^####^  -2.14-(-1.76) | **Treatment** | 0.68 | 0.42 |
|  |  |  |  |  | **Time** | 22.80 | <0.0001 |
|  |  |  |  |  | **Treatment X time** | 9.43 | 0.004 |
| C18/C18:1 | -0.36  -0.47-(-0.19) | -0.43  -0.65-(-0.31) | -0.37  -0.56-(-0.20) | -0.43  -0.59-(-0.33) | **Treatment** | 4.80 | 0.04 |
|  |  |  |  |  | **Time** | 0.32 | 0.58 |
|  |  |  |  |  | **Treatment X time** | 0.26 | 0.61 |
| C16 + C18 | -0.21  -0.35-(-0.14) | -0.17  -0.38-0.06 | -0.33  -0.52-(-0.12) | -0.14***  -0.29-0.15 | **Treatment** | 13.93 | 0.0007 |
|  |  |  |  |  | **Time** | 0.001 | 0.98 |
|  |  |  |  |  | **Treatment X time** | 4.97 | 0.03 |
| C14 + C16 + C18 | -0.13  -0.26-(-0.05) | -0.11  -0.29-0.14 | -0.23  -0.41-(-0.04) | -0.06**  -0.21-0.23 | **Treatment** | 11.98 | 0.002 |
|  |  |  |  |  | **Time** | 0.00 | 1.00 |
|  |  |  |  |  | **Treatment X time** | 4.65 | 0.04 |
| C14 + C16 + C18/C0 | -1.69  -1.73-(-1.63) | -1.62  -1.69-(-1.36) | -1.71  -1.82-(-1.41) | -1.31**  -1.73-(-0.93) | **Treatment** | 14.15 | 0.0007 |
|  |  |  |  |  | **Time** | 4.05 | 0.05 |
|  |  |  |  |  | **Treatment X time** | 3.64 | 0.065 |
| CRT-1 | -2.22  -2.28-(-2.07) | -2.07  -2.21-(-1.81) | -2.25  -2.40-(-1.96) | -1.84***  -2.29-(-1.41) | **Treatment** | 17.99 | 0.0002 |
|  |  |  |  |  | **Time** | 1.99 | 0.17 |
|  |  |  |  |  | **Treatment X time** | 3.28 | 0.08 |
| CPT-1 | -1.77  -1.81-(-1.70) | -1.69  -1.78-(-1.44) | -1.80  -1.92-(-1.50) | -1.38***  -1.81-(-1.00) | **Treatment** | 15.81 | 0.0004 |
|  |  |  |  |  | **Time** | 3.95 | 0.06 |
|  |  |  |  |  | **Treatment X time** | 3.91 | 0.06 |
| Hydroxylated LCACs | -0.77  -0.88-(-0.63) | -0.84  -0.94-(-0.70) | -0.79  -0.87-(-0.74) | -0.80  -0.85-(-0.65) | **Treatment** | 0.03 | 0.86 |
|  |  |  |  |  | **Time** | 1.06 | 0.31 |
|  |  |  |  |  | **Treatment X time** | 5.53 | 0.03 |
| Branched-chain amino acids (BCAA) | 2.76  2.70-2.91 | 2.73  2.57-2.88 | 2.87  2.78-3.03 | 2.80^#^  2.75-2.92 | **Treatment** | 8.96 | 0.005 |
|  |  |  |  |  | **Time** | 14.97 | 0.0005 |
|  |  |  |  |  | **Treatment X time** | 0.27 | 0.61 |
| Aromatic amino acids (AAA) | 2.23  2.14-2.33 | 2.12*  1.98-2.20 | 2.35^@@@^  2.28-2.58 | 2.33^####^  2.22-2.47 | **Treatment** | 12.36 | 0.001 |
|  |  |  |  |  | **Time** | 50.72 | <0.0001 |
|  |  |  |  |  | **Treatment X time** | 1.04 | 0.31 |
| BCAA/AAA | 0.57  0.50-0.63 | 0.60  0.53-0.72 | 0.49^@^  0.31-0.59 | 0.47^####^  0.37-0.59 | **Treatment** | 1.32 | 0.26 |
|  |  |  |  |  | **Time** | 26.73 | <0.0001 |
|  |  |  |  |  | **Treatment X time** | 0.62 | 0.43 |
| Glucogenic amino acids | 3.42  3.33-3.54 | 3.30*  3.21-3.39 | 3.46  3.33-3.64 | 3.45^##^  3.30-3.57 | **Treatment** | 9.79 | 0.004 |
|  |  |  |  |  | **Time** | 13.09 | 0.0009 |
|  |  |  |  |  | **Treatment X time** | 2.09 | 0.16 |
| Tyrosine/  Phenylalanine | -0.15  -0.21-(-0.04) | -0.20**  -0.29-(-0.09) | -0.02  -0.09-0.05 | -0.17***  -0.29-(-0.04) | **Treatment** | 27.91 | <0.0001 |
|  |  |  |  |  | **Time** | 10.59 | 0.003 |
|  |  |  |  |  | **Treatment X time** | 2.67 | 0.11 |
| Glycine/Serine | 0.37  0.29-0.57 | 0.55*  0.37-0.68 | 0.35  0.16-0.55 | 0.39^##^  0.14-0.47 | **Treatment** | 5.46 | 0.03 |
|  |  |  |  |  | **Time** | 12.27 | 0.001 |
|  |  |  |  |  | **Treatment X time** | 3.16 | 0.08 |
| Glutamine/  Glutamate | 0.64  0.09-0.88 | 0.54  0.05-0.83 | 0.60  0.34-0.87 | 0.74^#^  0.66-0.88 | **Treatment** | 0.29 | 0.59 |
|  |  |  |  |  | **Time** | 4.74 | 0.04 |
|  |  |  |  |  | **Treatment X time** | 3.47 | 0.07 |
| Asparagine/  Aspartate | -0.23  -0.47-(-0.12) | -0.37  -0.61-(-0.20) | -0.13  -0.29-0.08 | -0.09^####^  -0.21-0.11 | **Treatment** | 0.28 | 0.60 |
|  |  |  |  |  | **Time** | 30.98 | <0.0001 |
|  |  |  |  |  | **Treatment X time** | 5.51 | 0.02 |
| Arginine/Citrulline | 0.43  0.32-0.62 | 0.41  -0.03-0.58 | 0.38  0.22-0.79 | 0.72**^###^  0.42-1.08 | **Treatment** | 4.28 | 0.05 |
|  |  |  |  |  | **Time** | 9.16 | 0.005 |
|  |  |  |  |  | **Treatment X time** | 10.63 | 0.003 |
| ADMA/Arginine | -2.32  -2.47-(-2.06) | -2.12  -2.38-(-1.97) | -2.52^@^  -2.84-(-2.35) | -2.58^###^  -2.73-(-2.06) | **Treatment** | 3.53 | 0.07 |
|  |  |  |  |  | **Time** | 33.60 | <0.0001 |
|  |  |  |  |  | **Treatment X time** | 1.89 | 0.18 |
| Methionine/  Methionine-SO | 1.98  1.77-2.12 | 2.07  1.31-2.23 | 2.00  1.78-2.33 | 2.21  2.01-2.69 | **Treatment** | 1.92 | 0.18 |
|  |  |  |  |  | **Time** | 5.59 | 0.03 |
|  |  |  |  |  | **Treatment X time** | 2.82 | 0.10 |
| Spermidine/  Putrescine | 0.46  0.39-0.65 | 0.82****  0.71-0.88 | 0.61  0.38-0.76 | 0.35****^####^  0.08-0.45 | **Treatment** | 0.21 | 0.65 |
|  |  |  |  |  | **Time** | 36.62 | <0.0001 |
|  |  |  |  |  | **Treatment X time** | 89.05 | <0.0001 |
| Serotonin/  Kynurenine | 1.37  0.36-1.56 | 1.38  0.95-1.68 | 1.35  0.92-1.47 | 0.62****^####^  -0.14-0.98 | **Treatment** | 12.15 | 0.001 |
|  |  |  |  |  | **Time** | 21.27 | <0.0001 |
|  |  |  |  |  | **Treatment X time** | 22.36 | <0.0001 |
| Kynurenine/Alpha-Aminoadipic acid | -0.91  -1.08-(-0.73) | -0.88  -0.99-(-0.67) | -0.75  -1.10-(-0.64) | -0.41****^####^  -0.53-(-0.07) | **Treatment** | 29.04 | <0.0001 |
|  |  |  |  |  | **Time** | 47.69 | <0.0001 |
|  |  |  |  |  | **Treatment X time** | 18.60 | 0.0001 |
| Leucine/Kynurenine | 2.34  2.21-2.42 | 2.33  2.12-2.59 | 2.33  2.20-2.50 | 1.96****^####^  1.72-2.16 | **Treatment** | 30.38 | <0.0001 |
|  |  |  |  |  | **Time** | 26.52 | <0.0001 |
|  |  |  |  |  | **Treatment X time** | 29.46 | <0.0001 |
| Kynurenine/  Tryptophan | -2.09  -2.15-(-1.84) | -2.11  -2.32-(-1.86) | -2.08  -2.31-(-1.96) | -1.64****^####^  -1.89-(-1.35) | **Treatment** | 27.14 | <0.0001 |
|  |  |  |  |  | **Time** | 22.13 | <0.0001 |
|  |  |  |  |  | **Treatment X time** | 34.61 | <0.0001 |
| Serotonin/  Tryptophan | -0.61  -0.95-(-0.54) | -0.66  -0.78-(-0.58) | -0.83  -1.07-(-0.70) | -1.14**^####^  -1.49-(-0.83) | **Treatment** | 7.21 | 0.01 |
|  |  |  |  |  | **Time** | 37.31 | <0.0001 |
|  |  |  |  |  | **Treatment X time** | 8.89 | 0.005 |
| lysoPC a C16:1/ lysoPC a C16:0 | -1.58  -1.67-(-1.42) | -1.57  -1.67-(-1.50) | -1.56  -1.76-(-1.40) | -1.74****^##^  -1.83-(-1.61) | **Treatment** | 14.74 | 0.0005 |
|  |  |  |  |  | **Time** | 4.35 | 0.045 |
|  |  |  |  |  | **Treatment X time** | 11.75 | 0.002 |
| lysoPC a C18:2/  lysoPC a C18:1 | 0.44  0.36-0.51 | 0.38  0.33-0.44 | 0.45  0.30-0.52 | 0.35**  0.28-0.40 | **Treatment** | 18.42 | 0.0002 |
|  |  |  |  |  | **Time** | 1.50 | 0.23 |
|  |  |  |  |  | **Treatment X time** | 0.94 | 0.34 |
| lysoPC a C20:4/ lysoPC a C20:3 | 0.59  0.58-0.61 | 0.62  0.55-0.87 | 0.55  0.39-0.70 | 0.77****^#^  0.62-0.88 | **Treatment** | 25.76 | <0.0001 |
|  |  |  |  |  | **Time** | 2.65 | 0.11 |
|  |  |  |  |  | **Treatment X time** | 9.86 | 0.004 |

# Supplementary Table 2

Two-way ANOVA [treatment (saline or LPS) x time (1.5 h or 24 h LPS challenge)] summary table of 1.5 h and 24 h LPS induced changes in metabolite levels (log10 values, median and range) and their ratios in 129Sv strain. Bonferroni post-hoc test was used for multiple comparisons. * Statistically significant difference between control and LPS in 1.5 or 24 administration groups; ^#^ Statistically significant difference between LPS administration groups. ^@^ Statistically significant difference between saline administration groups. * p ≤ 0.05; ** p ≤ 0.01; *** p ≤ 0.001; **** p ≤ 0.0001

| **Metabolites** | **129Sv** | | | | | | | | **Two-way ANOVA** | | | | | |  |
| --- | --- | --- | --- | --- | --- | --- | --- | --- | --- | --- | --- | --- | --- | --- | --- |
|  | **1.5 h** | | | | **24 h** | | | |  |  |  |  |  |  |  |
|  | **Control**  **Median**  **(min – max)** | | **LPS**  **Median**  **(min – max)** | | **Control**  **Median**  **(min – max)** | | **LPS**  **Median**  **(min – max)** | |  |  |  |  |  |  |  |
|  |  |  |  |  |  |  |  |  |  | | ***F*** | | ***p*** | |  |
| **Acylcarnitines** | | | | | | | | | | | | | | |  |
| Carnitine (C0) | | 1.52  1.37-1.60 | | 1.48  1.39-1.52 | | 1.50  1.36-1.58 | | 1.33**^##^  1.11-1.47 | | **Treatment** | | 13.09 | | <0.0001 | |
|  |  |  |  |  |  |  |  |  |  | **Time** | | 10.46 | | 0.003 | |
|  |  |  |  |  |  |  |  |  |  | **Treatment X time** | | 4.75 | | 0.04 | |
| Acetylcarnitine (C2) | | 1.36  1.21-1.46 | | 1.18**  1.09-1.31 | | 1.32  1.26-1.46 | | 1.37^##^  1.07-1.47 | | **Treatment** | | 7.49 | | 0.01 | |
|  |  |  |  |  |  |  |  |  |  | **Time** | | 6.82 | | 0.01 | |
|  |  |  |  |  |  |  |  |  |  | **Treatment X time** | | 8.98 | | 0.005 | |
| Propionylcarnitine (C3) | | -0.09  -0.15-0.10 | | -0.26****  -0.30-(-0.20) | | -0.10  -0.18-(-0.04) | | -0.19***  -0.39-(-0.14) | | **Treatment** | | 52.03 | | <0.0001 | |
|  |  |  |  |  |  |  |  |  |  | **Time** | | 0.0002 | | 0.99 | |
|  |  |  |  |  |  |  |  |  |  | **Treatment X time** | | 1.25 | | 0.27 | |
| Hydroxybutyryl-carnitine (C3-DC) | | -0.93  -0.97-(-0.73) | | -0.93  -1.07-(-0.81) | | -0.96  -1.04-(-0.76) | | -0.80  -0.93-(-0.67) | | **Treatment** | | 0.45 | | 0.51 | |
|  |  |  |  |  |  |  |  |  |  | **Time** | | 0.20 | | 0.66 | |
|  |  |  |  |  |  |  |  |  |  | **Treatment X time** | | 2.72 | | 0.11 | |
| Hydroxypropionyl-carnitine (C3-OH) | | -1.20  -1.29-(-0.73) | | -1.22  -1.34-(-0.78) | | -1.20  -1.35-(-0.90) | | -1.15  -1.46-(-0.84) | | **Treatment** | | 0.72 | | 0.40 | |
|  |  |  |  |  |  |  |  |  |  | **Time** | | 0.13 | | 0.72 | |
|  |  |  |  |  |  |  |  |  |  | **Treatment X time** | | 0.03 | | 0.87 | |
| Butyryl- and isobutyrylcarnitine (C4-) | | -0.06  -0.16-0.01 | | -0.20**  -0.27-(-0.11) | | -0.01  -0.09-0.07 | | -0.05^###^  -0.23-0.08 | | **Treatment** | | 15.73 | | 0.0004 | |
|  |  |  |  |  |  |  |  |  |  | **Time** | | 22.10 | | <0.0001 | |
|  |  |  |  |  |  |  |  |  |  | **Treatment X time** | | 3.62 | | 0.07 | |
| Propenoylcarnitine (C3:1) | | -1.28  -1.32-(-1.19) | | -1.27  -1.33-(-1.19) | | -1.29  -1.36-(-1.18) | | -1.21  -1.38-(-1.01) | | **Treatment** | | 2.83 | | 0.10 | |
|  |  |  |  |  |  |  |  |  |  | **Time** | | 0.86 | | 0.36 | |
|  |  |  |  |  |  |  |  |  |  | **Treatment X time** | | 1.83 | | 0.19 | |
| Butenoylcarnitine (C4:1) | | -1.04  -1.12-(-0.95) | | -1.05  -1.11-(-0.90) | | -1.03  -1.18-(-0.97) | | -1.05  -1.19-(-0.81) | | **Treatment** | | 0.22 | | 0.64 | |
|  |  |  |  |  |  |  |  |  |  | **Time** | | 0.25 | | 0.62 | |
|  |  |  |  |  |  |  |  |  |  | **Treatment X time** | | 0.001 | | 0.98 | |
| Isovalerylcarnitine and 2-methybutyrylcarnitine (C5-) | | -0.35  -0.41-(-0.20) | | -0.50***  -0.60-(-0.38) | | -0.39  -0.47-(-0.23) | | -0.35^##^  -0.50-(-0.22) | | **Treatment** | | 8.51 | | 0.006 | |
|  |  |  |  |  |  |  |  |  |  | **Time** | | 3.04 | | 0.091 | |
|  |  |  |  |  |  |  |  |  |  | **Treatment X time** | | 12.80 | | 0.001 | |
| Glutarylcarnitine (C5-DC) | | -1.52  -1.57-(-1.43) | | -1.50  -1.62-(-1.38) | | -1.55  -1. 70-(-1.39) | | -1.50  -1.64-(-1.32) | | **Treatment** | | 1.71 | | 0.20 | |
|  |  |  |  |  |  |  |  |  |  | **Time** | | 0.15 | | 0.70 | |
|  |  |  |  |  |  |  |  |  |  | **Treatment X time** | | 0.23 | | 0.64 | |
| Hydroxyvaleryl-carnitine (C5-OH) | | -0.95  -0.99-(-0.83) | | -0.93  -1.00-(-0.87) | | -0.95  -1.04-(-0.90) | | -0.94  -1.09-(-0.84) | | **Treatment** | | 0.51 | | 0.48 | |
|  |  |  |  |  |  |  |  |  |  | **Time** | | 2.29 | | 0.14 | |
|  |  |  |  |  |  |  |  |  |  | **Treatment X time** | | 0.08 | | 0.78 | |
| Tiglylcarnitine (C5:1) | | -1.20  -1.24-(-1.10) | | -1.14  -1.24-(-1.07) | | -1.16  -1.27-(-1.10) | | -1.19  -1.24-(-1.03) | | **Treatment** | | 1.08 | | 0.31 | |
|  |  |  |  |  |  |  |  |  |  | **Time** | | 0.03 | | 0.86 | |
|  |  |  |  |  |  |  |  |  |  | **Treatment X time** | | 0.62 | | 0.43 | |
| Glutaconylcarnitine (C5:1-DC) | | -1.45  -1.57-(-1.23) | | -1.43  -1.52-(-1.34) | | -1.48  -1.62-(-1.32) | | -1.48  -1.59-(-1.25) | | **Treatment** | | 0.24 | | 0.63 | |
|  |  |  |  |  |  |  |  |  |  | **Time** | | 0.75 | | 0.39 | |
|  |  |  |  |  |  |  |  |  |  | **Treatment X time** | | 0.20 | | 0.66 | |
| Hexanoylcarnitine (C6) | | -1.55  -1.68-(-1.43) | | -1.61  -1.64-(-1.54) | | -1.54  -1.68-(-1.47) | | -1.54  -1.72-(-1.38) | | **Treatment** | | 0.19 | | 0.66 | |
|  |  |  |  |  |  |  |  |  |  | **Time** | | 1.57 | | 0.22 | |
|  |  |  |  |  |  |  |  |  |  | **Treatment X time** | | 2.91 | | 0.10 | |
| Hexenoylcarnitine (C6:1) | | -1.94  -2.10-(-1.89) | | -1.96  -2.05-(-1.92) | | -1.98  -2.05-(-1.92) | | -2.00  -2.05-(-1.92) | | **Treatment** | | 0.19 | | 0.66 | |
|  |  |  |  |  |  |  |  |  |  | **Time** | | 1.57 | | 0.22 | |
|  |  |  |  |  |  |  |  |  |  | **Treatment X time** | | 2.91 | | 0.10 | |
| Pimelylcarnitine (C7-DC) | | -1.54  -1.64-(-1.38) | | -1.51  -1.57-(-1.47) | | -1.51  -1.59-(-1.41) | | -1.55  -1.60-(-1.28) | | **Treatment** | | 0.19 | | 0.66 | |
|  |  |  |  |  |  |  |  |  |  | **Time** | | 1.57 | | 0.22 | |
|  |  |  |  |  |  |  |  |  |  | **Treatment X time** | | 2.91 | | 0.10 | |
| Octanoylcarnitine (C8) | | -0.80  -0.88-(-0.70) | | -0.81  -0.92-(-0.71) | | -0.85  -0.91-(-0.75) | | -0.81  -0.88-(-0.65) | | **Treatment** | | 0.66 | | 0.42 | |
|  |  |  |  |  |  |  |  |  |  | **Time** | | 0.15 | | 0.70 | |
|  |  |  |  |  |  |  |  |  |  | **Treatment X time** | | 1.02 | | 0.32 | |
| Nonanoylcarnitine (C9) | | -1.07  -1.21-(-0.98) | | -1.06  -1.17-(-0.98) | | -1.07  -1.14-(-1.00) | | -1.04  -1.14-(-0.91) | | **Treatment** | | 0.82 | | 0.37 | |
|  |  |  |  |  |  |  |  |  |  | **Time** | | 1.19 | | 0.28 | |
|  |  |  |  |  |  |  |  |  |  | **Treatment X time** | | 0.21 | | 0.65 | |
| Decanoylcarnitine (C10) | | -0.59  -0.67-(-0.45) | | -0.56  -0.71-(-0.29) | | -0.46  -0.60-(-0.31) | | -0.44  -0.63-(-0.27) | | **Treatment** | | 1.20 | | 0.28 | |
|  |  |  |  |  |  |  |  |  |  | **Time** | | 5.07 | | 0.03 | |
|  |  |  |  |  |  |  |  |  |  | **Treatment X time** | | 0.06 | | 0.80 | |
| Decenoylcarnitine (C10:1) | | -1.01  -1.06-(-0.95) | | -1.00  -1.11-(-0.84) | | -0.98  -1.13-(-0.84) | | -0.99  -1.07-(-0.82) | | **Treatment** | | 0.67 | | 0.42 | |
|  |  |  |  |  |  |  |  |  |  | **Time** | | 0.49 | | 0.49 | |
|  |  |  |  |  |  |  |  |  |  | **Treatment X time** | | 0.04 | | 0.85 | |
| Decadienyl-carnitine (C10:2) | | -0.93  -1.00-(-0.81) | | -0.93  -1.01-(-0.77) | | -0.90  -1.10-(-0.67) | | -0.88  -0.97-(-0.83) | | **Treatment** | | 0.67 | | 0.42 | |
|  |  |  |  |  |  |  |  |  |  | **Time** | | 0.49 | | 0.49 | |
|  |  |  |  |  |  |  |  |  |  | **Treatment X time** | | 0.04 | | 0.85 | |
| Dodecanoyl-carnitine (C12) | | -1.03  -1.11-(-0.96) | | -1.00  -1.12-(-0.90) | | -1.02  -1.10-(-0.90) | | -0.91*^#^  -1.00-(-0.82) | | **Treatment** | | 7.23 | | 0.01 | |
|  |  |  |  |  |  |  |  |  |  | **Time** | | 7.43 | | 0.01 | |
|  |  |  |  |  |  |  |  |  |  | **Treatment X time** | | 2.18 | | 0.15 | |
| Dodecanedioyl-carnitine C12-DC | | -0.51  -0.56-(-0.42) | | -0.47  -0.55-(-0.40) | | -0.50  -0.60-(-0.37) | | -0.49  -0.53-(-0.36) | | **Treatment** | | 1.02 | | 0.32 | |
|  |  |  |  |  |  |  |  |  |  | **Time** | | 0.17 | | 0.68 | |
|  |  |  |  |  |  |  |  |  |  | **Treatment X time** | | 0.12 | | 0.73 | |
| Dodecenoyl-carnitine (C12:1) | | -0.84  -1.06-(-0.64) | | -0.82  -1.05-(-0.74) | | -0.82  -0.93-(-0.75) | | -0.78  -0.85-(-0.54) | | **Treatment** | | 0.89 | | 0.35 | |
|  |  |  |  |  |  |  |  |  |  | **Time** | | 3.32 | | 0.08 | |
|  |  |  |  |  |  |  |  |  |  | **Treatment X time** | | 0.94 | | 0.34 | |
| Tetradecanoyl-carnitine (C14) | | -1.31  -1.46-(-1.15) | | -1.30  -1.48-(-1.13) | | -1.26  -1.42-(-1.11) | | -1.14  -1.38-(-0.97) | | **Treatment** | | 3.22 | | 0.08 | |
|  |  |  |  |  |  |  |  |  |  | **Time** | | 5.60 | | 0.02 | |
|  |  |  |  |  |  |  |  |  |  | **Treatment X time** | | 2.10 | | 0.16 | |
| Tetradecenoyl-carnitine (C14:1) | | -1.48  -1.55-(-1.19) | | -1.41  -1.54-(-1.29) | | -1.45  -1.54-(-1.24) | | -1.12***^###^  -1.40-(-1.03) | | **Treatment** | | 14.82 | | 0.001 | |
|  |  |  |  |  |  |  |  |  |  | **Time** | | 12.24 | | 0.001 | |
|  |  |  |  |  |  |  |  |  |  | **Treatment X time** | | 9.81 | | 0.004 | |
| Hydroxytetra-decenoylcarnitine (C14:1-OH) | | -1.73  -1.89-(-1.59) | | -1.73  -1.82-(-1.66) | | -1.77  -1.89-(-1.64) | | -1.62***^##^  -1.66-(-1.37) | | **Treatment** | | 9.28 | | 0.005 | |
|  |  |  |  |  |  |  |  |  |  | **Time** | | 5.00 | | 0.03 | |
|  |  |  |  |  |  |  |  |  |  | **Treatment X time** | | 11.38 | | 0.002 | |
| Tetradecadienyl-carnitine (C14:2) | | -1.82  -2.22-(-1.70) | | -1.87  -1.96-(-1.68) | | -1.96  -2.22-(-1.64) | | -1.74**  -1.96-(-1.38) | | **Treatment** | | 7.51 | | 0.01 | |
|  |  |  |  |  |  |  |  |  |  | **Time** | | 0.78 | | 0.38 | |
|  |  |  |  |  |  |  |  |  |  | **Treatment X time** | | 5.15 | | 0.03 | |
| Hydroxytetra-decadienylcarnitine (C14:2-OH) | | -1.80  -1.85-(-1.72) | | -1.92  -2.00-(-1.66) | | -1.84  -1.96-(-1.70) | | -1.64*^##^  -1.89-(-1.456) | | **Treatment** | | 1.18 | | 0.29 | |
|  |  |  |  |  |  |  |  |  |  | **Time** | | 5.53 | | 0.02 | |
|  |  |  |  |  |  |  |  |  |  | **Treatment X time** | | 12.25 | | 0.001 | |
| Hexadecanoyl-carnitine (C16) | | -0.72  -0.79-(-0.64) | | -0.70  -0.77-(-0.60) | | -0.78  -0.91-(-0.69) | | -0.63**** -0.71-(-0.44) | | **Treatment** | | 18.56 | | 0.0001 | |
|  |  |  |  |  |  |  |  |  |  | **Time** | | 0.15 | | 0.70 | |
|  |  |  |  |  |  |  |  |  |  | **Treatment X time** | | 10.88 | | 0.002 | |
| Hydroxyhexa-decanoylcarnitine (C16-OH) | | -1.36  -1.49-(-1.21) | | -1.34  -1.55-(-1.23) | | -1.34  -1.41-(-1.24) | | -1.18**^###^  -1.29-(-1.06) | | **Treatment** | | 8.79 | | 0.006 | |
|  |  |  |  |  |  |  |  |  |  | **Time** | | 14.89 | | 0.0005 | |
|  |  |  |  |  |  |  |  |  |  | **Treatment X time** | | 4.86 | | 0.03 | |
| Hexadecenoyl-carnitine (C16:1) | | -1.21  -1.40-(-1.07) | | -1.25  -1.34-(-1.12) | | -1.28  -1.35-(-1.06) | | -0.92***^###^  -1.26-(-0.80) | | **Treatment** | | 8.19 | | 0.007 | |
|  |  |  |  |  |  |  |  |  |  | **Time** | | 7.98 | | 0.008 | |
|  |  |  |  |  |  |  |  |  |  | **Treatment X time** | | 10.85 | | 0.002 | |
| Hydroxyhexa-decenoylcarnitine (C16:1-OH) | | -1.65  -1.74-(-1.57) | | -1.61  -1.72-(-1.49) | | -1.70  -1.89-(-1.55) | | -1.49****^#^  -1.72-(-1.28) | | **Treatment** | | 20.25 | | <0.0001 | |
|  |  |  |  |  |  |  |  |  |  | **Time** | | 1.83 | | 0.18 | |
|  |  |  |  |  |  |  |  |  |  | **Treatment X time** | | 7.66 | | 0.009 | |
| Hexadecadienyl-carnitine (C16:2) | | -1.51  -1.57-(-1.38) | | -1.48  -1.62-(-1.39) | | -1.44  -1.62-(-1.40) | | -1.32**^##^  -1.49-(-1.10) | | **Treatment** | | 8.71 | | 0.006 | |
|  |  |  |  |  |  |  |  |  |  | **Time** | | 9.20 | | 0.005 | |
|  |  |  |  |  |  |  |  |  |  | **Treatment X time** | | 8.08 | | 0.008 | |
| Hydroxyhexa-decadienylcarnitine (C16:2-OH) | | -1.56  -1.68-(-1.40) | | -1.58  -1.64-(-1.52) | | -1.59  -1.74-(-1.49) | | -1.49**^#^  -1.60-(-1.37) | | **Treatment** | | 5.38 | | 0.03 | |
|  |  |  |  |  |  |  |  |  |  | **Time** | | 1.47 | | 0.23 | |
|  |  |  |  |  |  |  |  |  |  | **Treatment X time** | | 10.98 | | 0.002 | |
| Octadecanoylcarnitine (C18) | | -1.27  -1.37-(-1.12) | | -1.24  -1.36-(-1.14) | | -1.39  -1.49-(-1.23) | | -1.18***  -1.33-(-1.01) | | **Treatment** | | 11.00 | | 0.002 | |
|  |  |  |  |  |  |  |  |  |  | **Time** | | 0.68 | | 0.41 | |
|  |  |  |  |  |  |  |  |  |  | **Treatment X time** | | 8.96 | | 0.005 | |
| Octadecenoyl-carnitine (C18:1) | | -0.97  -1.17-(-0.80) | | -0.91  -1.01-(-0.81) | | -0.97  -1.17-(-0.80) | | -0.91****^#^  -1.01-(-0.81) | | **Treatment** | | 20.79 | | <0.0001 | |
|  |  |  |  |  |  |  |  |  |  | **Time** | | 2.54 | | 0.12 | |
|  |  |  |  |  |  |  |  |  |  | **Treatment X time** | | 8.54 | | 0.006 | |
| Hydroxyocta-decenoylcarnitine (C18:1-OH) | | -1.50  -1.60-(-1.42) | | -1.46  -1.54-(-1.32) | | -1.57  -1.74-(-1.37) | | -1.40**  -1.60-(-1.18) | | **Treatment** | | 13.32 | | 0.0009 | |
|  |  |  |  |  |  |  |  |  |  | **Time** | | 0.04 | | 0.85 | |
|  |  |  |  |  |  |  |  |  |  | **Treatment X time** | | 3.47 | | 0.07 | |
| Octadecadienyl-carnitine (C18:2) | | -1.26  -1.43-(-1.14) | | -1.21  -1.36-(-1.08) | | -1.29  -1.49-(-1.00) | | -0.92***^##^  -1.24-(-0.68) | | **Treatment** | | 15.92 | | 0.0003 | |
|  |  |  |  |  |  |  |  |  |  | **Time** | | 6.26 | | 0.02 | |
|  |  |  |  |  |  |  |  |  |  | **Treatment X time** | | 7.98 | | 0.008 | |
| **Amino Acids** | | | | | | | | | | | | | | |  |
| Alanine (Ala) | 2.72  2.59-2.94 | | 2.58  2.35-3.01 | | 2.69  2.60-2.89 | | 2.82  2.56-2.92 | | **Treatment** | | 0.26 | | 0.61 | |  |
|  |  |  |  |  |  |  |  |  | **Time** | | 1.90 | | 0.18 | |  |
|  |  |  |  |  |  |  |  |  | **Treatment X time** | | 4.86 | | 0.03 | |  |
| Arginine (Arg) | 2.23  1.86-2.60 | | 2.09  1.80-2.70 | | 2.20  1.97-2.58 | | 2.62^#^  2.10-2.76 | | **Treatment** | | 1.66 | | 0.21 | |  |
|  |  |  |  |  |  |  |  |  | **Time** | | 4.44 | | 0.04 | |  |
|  |  |  |  |  |  |  |  |  | **Treatment X time** | | 4.29 | | 0.05 | |  |
| Asparagine (Asn) | 1.73  1.39-2.10 | | 1.65  1.42-2.20 | | 1.66  1.49-2.08 | | 1.89  1.45-2.16 | | **Treatment** | | 0.05 | | 0.83 | |  |
|  |  |  |  |  |  |  |  |  | **Time** | | 0.05 | | 0.83 | |  |
|  |  |  |  |  |  |  |  |  | **Treatment X time** | | 1.05 | | 0.31 | |  |
| Aspartate (Asp) | 1.84  1.68-2.05 | | 1.81  1.65-2.30 | | 1.70  1.40-1.80 | | 1.65^##^  1.33-1.78 | | **Treatment** | | 0.05 | | 0.82 | |  |
|  |  |  |  |  |  |  |  |  | **Time** | | 18.97 | | 0.0001 | |  |
|  |  |  |  |  |  |  |  |  | **Treatment X time** | | 0.75 | | 0.39 | |  |
| Citrulline (Cit) | 1.73  1.50-1.99 | | 1.64*  1.51-1.75 | | 1.75  1.66-1.83 | | 1.55****  1.45-1.63 | | **Treatment** | | 42.36 | | <0.0001 | |  |
|  |  |  |  |  |  |  |  |  | **Time** | | 4.14 | | 0.05 | |  |
|  |  |  |  |  |  |  |  |  | **Treatment X time** | | 3.05 | | 0.09 | |  |
| Glutamine (Gln) | 2.75  2.58-2.91 | | 2.76  2.56-2.96 | | 2.69  2.58-2.79 | | 2.80  2.74-2.90 | | **Treatment** | | 3.80 | | 0.06 | |  |
|  |  |  |  |  |  |  |  |  | **Time** | | 0.02 | | 0.88 | |  |
|  |  |  |  |  |  |  |  |  | **Treatment X time** | | 1.63 | | 0.21 | |  |
| Glutamate (Glu) | 2.34  1.97-2.77 | | 2.37  1.99-2.69 | | 2.27  2.06-2.43 | | 2.26  1.83-2.53 | | **Treatment** | | 0.30 | | 0.59 | |  |
|  |  |  |  |  |  |  |  |  | **Time** | | 1.57 | | 0.22 | |  |
|  |  |  |  |  |  |  |  |  | **Treatment X time** | | 0.03 | | 0.86 | |  |
| Glycine (Gly) | 2.67  2.53-2.81 | | 2.56  2.39-2.79 | | 2.49  2.38-2.63 | | 2.55  2.43-2.74 | | **Treatment** | | 0.04 | | 0.85 | |  |
|  |  |  |  |  |  |  |  |  | **Time** | | 2.90 | | 0.10 | |  |
|  |  |  |  |  |  |  |  |  | **Treatment X time** | | 4.18 | | 0.05 | |  |
| Histidine (His) | 2.01  1.89-2.26 | | 1.92  1.83-2.30 | | 1.99  1.92-2.23 | | 2.00  1.82-2.32 | | **Treatment** | | 1.14 | | 0.29 | |  |
|  |  |  |  |  |  |  |  |  | **Time** | | 0.06 | | 0.80 | |  |
|  |  |  |  |  |  |  |  |  | **Treatment X time** | | 0.34 | | 0.56 | |  |
| Isoleucine (Ile) | 2.16  2.06-2.39 | | 2.07  1.87-2.46 | | 2.15  2.00-2.33 | | 2.27  2.01-2.53 | | **Treatment** | | 0.03 | | 0.86 | |  |
|  |  |  |  |  |  |  |  |  | **Time** | | 0.94 | | 0.34 | |  |
|  |  |  |  |  |  |  |  |  | **Treatment X time** | | 3.72 | | 0.06 | |  |
| Leucine (Leu) | 2.40  2.30-2.76 | | 2.36  1.93-2.86 | | 2.37  2.20-2.72 | | 2.61  2.29-2.87 | | **Treatment** | | 0.22 | | 0.65 | |  |
|  |  |  |  |  |  |  |  |  | **Time** | | 0.65 | | 0.43 | |  |
|  |  |  |  |  |  |  |  |  | **Treatment X time** | | 3.95 | | 0.06 | |  |
| Lysine (Lys) | 2.49  2.34-2.80 | | 2.35  2.16-2.90 | | 2.46  2.41-2.91 | | 2.88^#^  2.34-2.99 | | **Treatment** | | 1.47 | | 0.23 | |  |
|  |  |  |  |  |  |  |  |  | **Time** | | 5.55 | | 0.02 | |  |
|  |  |  |  |  |  |  |  |  | **Treatment X time** | | 4.74 | | 0.04 | |  |
| Methionine (Met) | 2.09  1.79-2.43 | | 2.03  1.81-2.43 | | 1.98  1.85-2.42 | | 2.35  1.92-2.55 | | **Treatment** | | 1.02 | | 0.32 | |  |
|  |  |  |  |  |  |  |  |  | **Time** | | 0.95 | | 0.34 | |  |
|  |  |  |  |  |  |  |  |  | **Treatment X time** | | 3.42 | | 0.07 | |  |
| Ornithine (Orn) | 1.99  1.85-2.19 | | 1.97  1.75-2.19 | | 1.92  1.84-2.04 | | 1.89  1.54-1.97 | | **Treatment** | | 2.95 | | 0.10 | |  |
|  |  |  |  |  |  |  |  |  | **Time** | | 7.75 | | 0.009 | |  |
|  |  |  |  |  |  |  |  |  | **Treatment X time** | | 1.15 | | 0.29 | |  |
| Phenylalanine (Phe) | 1.97  1.87-2.38 | | 1.96  1.73-2.49 | | 1.98  1.86-2.33 | | 2.20  1.94-2.54 | | **Treatment** | | 0.99 | | 0.33 | |  |
|  |  |  |  |  |  |  |  |  | **Time** | | 0.78 | | 0.38 | |  |
|  |  |  |  |  |  |  |  |  | **Treatment X time** | | 2.58 | | 0.12 | |  |
| Proline (Pro) | 2.16  1.89-2.49 | | 1.98  1.72-2.54 | | 2.03  1.92-2.43 | | 2.33  1.96-2.51 | | **Treatment** | | 0.05 | | 0.82 | |  |
|  |  |  |  |  |  |  |  |  | **Time** | | 0.44 | | 0.51 | |  |
|  |  |  |  |  |  |  |  |  | **Treatment X time** | | 4.24 | | 0.05 | |  |
| Serine (Ser) | 2.26  2.12-2.63 | | 2.18  2.01-2.68 | | 2.24  2.14-2.48 | | 2.39  2.10-2.57 | | **Treatment** | | 0.09 | | 0.77 | |  |
|  |  |  |  |  |  |  |  |  | **Time** | | 0.27 | | 0.61 | |  |
|  |  |  |  |  |  |  |  |  | **Treatment X time** | | 0.73 | | 0.40 | |  |
| Threonine (Thr) | 2.36  2.19-2.54 | | 2.25  2.04-2.57 | | 2.37  2.24-2.53 | | 2.48  2.27-2.59 | | **Treatment** | | 0.49 | | 0.49 | |  |
|  |  |  |  |  |  |  |  |  | **Time** | | 3.08 | | 0.09 | |  |
|  |  |  |  |  |  |  |  |  | **Treatment X time** | | 2.56 | | 0.12 | |  |
| Tryptophan (Trp) | 2.15  2.04-2.34 | | 2.16  2.03-2.34 | | 2.13  2.03-2.26 | | 2.16  1.89-2.27 | | **Treatment** | | 0.08 | | 0.78 | |  |
|  |  |  |  |  |  |  |  |  | **Time** | | 0.53 | | 0.47 | |  |
|  |  |  |  |  |  |  |  |  | **Treatment X time** | | 0.007 | | 0.93 | |  |
| Tyrosine (Tyr) | 1.87  1.71-2.28 | | 1.78  1.55-2.37 | | 1.95  1.76-2.26 | | 2.10  1.68-2.43 | | **Treatment** | | 0.10 | | 0.75 | |  |
|  |  |  |  |  |  |  |  |  | **Time** | | 3.25 | | 0.08 | |  |
|  |  |  |  |  |  |  |  |  | **Treatment X time** | | 1.73 | | 0.20 | |  |
| Valine (Val) | 2.48  2.35-2.66 | | 2.40  2.30-2.70 | | 2.46  2.30-2.66 | | 2.49  2.23-2.72 | | **Treatment** | | 0.16 | | 0.69 | |  |
|  |  |  |  |  |  |  |  |  | **Time** | | 0.07 | | 0.80 | |  |
|  |  |  |  |  |  |  |  |  | **Treatment X time** | | 0.46 | | 0.50 | |  |
| **Biogenic Amines** | | | | | | | | | | | | | | |  |
| Acetylornithine (Ac-Orn) | 0.46  0.32-0.67 | | 0.39  0.23-0.69 | | 0.49  0.25-0.71 | | 0.43  0.34-0.76 | | **Treatment** | | 0.92 | | 0.34 | |  |
|  |  |  |  |  |  |  |  |  | **Time** | | 0.67 | | 0.42 | |  |
|  |  |  |  |  |  |  |  |  | **Treatment X time** | | 0.29 | | 0.59 | |  |
| Asymmetric dimethylarginine (ADMA) | -0.25  -0.40-(-0.06) | | -0.16  -0.48-0.04 | | -0.23  -0.38-0.26 | | -0.15  -0.44-(-0.05) | | **Treatment** | | 0.12 | | 0.73 | |  |
|  |  |  |  |  |  |  |  |  | **Time** | | 0.12 | | 0.74 | |  |
|  |  |  |  |  |  |  |  |  | **Treatment X time** | | 0.56 | | 0.46 | |  |
| Alpha-Aminoadipic acid  (alpha-AAA) | 0.38  0.13-0.55 | | 0.27  0.11-0.44 | | 0.42  0.33-0.58 | | 0.48  0.25-0.61 | | **Treatment** | | 1.30 | | 0.26 | |  |
|  |  |  |  |  |  |  |  |  | **Time** | | 7.08 | | 0.01 | |  |
|  |  |  |  |  |  |  |  |  | **Treatment X time** | | 1.25 | | 0.27 | |  |
| Carnosine | 0.56  0.19-0.69 | | 0.65  0.39-0.85 | | 0.33  0.19-0.57 | | 0.35  -0.05-0.51 | | **Treatment** | | 1.43 | | 0.24 | |  |
|  |  |  |  |  |  |  |  |  | **Time** | | 4.91 | | 0.03 | |  |
|  |  |  |  |  |  |  |  |  | **Treatment X time** | | 0.38 | | 0.54 | |  |
| Creatinine | 1.21  1.19-1.23 | | 1.20  1.19-1.23 | | 1.20  1.18-1.22 | | 1.22  1.20-1.25 | | **Treatment** | | 0.0002 | | 0.99 | |  |
|  |  |  |  |  |  |  |  |  | **Time** | | 0.44 | | 0.51 | |  |
|  |  |  |  |  |  |  |  |  | **Treatment X time** | | 0.40 | | 0.53 | |  |
| Histamine | 0.23  0.06-0.57 | | 0.33  0.13-0.48 | | 0.15  0.04-0.26 | | 0.19^#^  0.03-0.31 | | **Treatment** | | 0.19 | | 0.67 | |  |
|  |  |  |  |  |  |  |  |  | **Time** | | 13.70 | | 0.0008 | |  |
|  |  |  |  |  |  |  |  |  | **Treatment X time** | | 0.36 | | 0.55 | |  |
| Putrescine | 0.08  -0.01-0.17 | | -0.21  -0.61-0.35 | | 0.05  -0.07-0.58 | | 0.18^##^  0.01-0.40 | | **Treatment** | | 1.64 | | 0.21 | |  |
|  |  |  |  |  |  |  |  |  | **Time** | | 6.63 | | 0.02 | |  |
|  |  |  |  |  |  |  |  |  | **Treatment X time** | | 6.52 | | 0.02 | |  |
| Symmetric dimethylarginine (SDMA) | -0.22  -0.38-(-0.05) | | -0.25  -0.35-(-0.04) | | -0.23  -0.31-0.14 | | -0.24  -0.36-(-0.13) | | **Treatment** | | 0.13 | | 0.72 | |  |
|  |  |  |  |  |  |  |  |  | **Time** | | 0.21 | | 0.65 | |  |
|  |  |  |  |  |  |  |  |  | **Treatment X time** | | 0.01 | | 0.91 | |  |
| Serotonin (5-HT) | 1.21  1.07-1.46 | | 1.16  0.88-1.37 | | 1.01  0.64-1.20 | | 0.51**^###^  0.16-1.15 | | **Treatment** | | 14.88 | | 0.0005 | |  |
|  |  |  |  |  |  |  |  |  | **Time** | | 22.40 | | <0.0001 | |  |
|  |  |  |  |  |  |  |  |  | **Treatment X time** | | 3.90 | | 0.06 | |  |
| Spermidine | 0.76  0.70-1.23 | | 0.86  0.68-1.08 | | 0.70  0.52-0.82 | | 0.72  0.62-0.90 | | **Treatment** | | 0.24 | | 0.63 | |  |
|  |  |  |  |  |  |  |  |  | **Time** | | 6.31 | | 0.02 | |  |
|  |  |  |  |  |  |  |  |  | **Treatment X time** | | 0.46 | | 0.50 | |  |
| Spermine | 0.23  0.00-0.63 | | 0.33  0.14-0.47 | | 0.16  -0.06-0.29 | | 0.14  0.02-0.31 | | **Treatment** | | 0.33 | | 0.57 | |  |
|  |  |  |  |  |  |  |  |  | **Time** | | 9.65 | | 0.004 | |  |
|  |  |  |  |  |  |  |  |  | **Treatment X time** | | 0.001 | | 0.97 | |  |
| Trans-4-Hydroxyproline (t4-OH-Pro) | 1.20  0.98-1.38 | | 1.12  0.90-1.24 | | 1.07  0.82-1.30 | | 1.20  1.09-1.30 | | **Treatment** | | 0.29 | | 0.59 | |  |
|  |  |  |  |  |  |  |  |  | **Time** | | 0.06 | | 0.81 | |  |
|  |  |  |  |  |  |  |  |  | **Treatment X time** | | 7.64 | | 0.009 | |  |
| Taurine | 2.61  2.53-2.67 | | 2.61  2.53-2.65 | | 2.57  2.53-2.64 | | 2.59  2.56-2.66 | | **Treatment** | | 0.01 | | 0.91 | |  |
|  |  |  |  |  |  |  |  |  | **Time** | | 5.82 | | 0.02 | |  |
|  |  |  |  |  |  |  |  |  | **Treatment X time** | | 0.99 | | 0.33 | |  |
| Kynurenine | 0.09  -0.06-0.28 | | 0.03  -0.06-0.16 | | 0.20  0.04-0.40 | | 0.53****^####^  0.44-0.68 | | **Treatment** | | 18.01 | | 0.0002 | |  |
|  |  |  |  |  |  |  |  |  | **Time** | | 73.94 | | <0.0001 | |  |
|  |  |  |  |  |  |  |  |  | **Treatment X time** | | 33.00 | | <0.0001 | |  |
| Methionine sulfoxide (Met-SO) | -0.15  -0.66-0.51 | | -0.38  -0.78-0.46 | | -0.10  -0.43-0.34 | | 0.19  -0.43-0.38 | | **Treatment** | | 0.009 | | 0.93 | |  |
|  |  |  |  |  |  |  |  |  | **Time** | | 4.03 | | 0.05 | |  |
|  |  |  |  |  |  |  |  |  | **Treatment X time** | | 1.74 | | 0.20 | |  |
| **Glycerophospholipids** | | | | | | | | | | | | | | |  |
| ***Lysophosphatidylcholine acyls*** | | | | | | | | | | | | | | |  |
| lysoPC a C14:0 | 1.05  0.97-1.13 | | 1.06  0.89-1.19 | | 1.03  0.90-1.24 | | 1.00  0.88-1.14 | | **Treatment** | | 0.59 | | 0.45 | |  |
|  |  |  |  |  |  |  |  |  | **Time** | | 0.74 | | 0.40 | |  |
|  |  |  |  |  |  |  |  |  | **Treatment X time** | | 1.04 | | 0.31 | |  |
| lysoPC a C16:0 | 2.65  2.58-2.78 | | 2.67  2.57-2.76 | | 2.65  2.58-2.78 | | 2.58  2.49-2.68 | | **Treatment** | | 0.13 | | 0.72 | |  |
|  |  |  |  |  |  |  |  |  | **Time** | | 0.20 | | 0.66 | |  |
|  |  |  |  |  |  |  |  |  | **Treatment X time** | | 3.01 | | 0.09 | |  |
| lysoPC a C16:1 | 0.79  0.27-1.00 | | 0.91  0.73-1.01 | | 0.84  0.65-1.20 | | 0.85  0.76-0.97 | | **Treatment** | | 0.56 | | 0.46 | |  |
|  |  |  |  |  |  |  |  |  | **Time** | | 0.30 | | 0.59 | |  |
|  |  |  |  |  |  |  |  |  | **Treatment X time** | | 2.41 | | 0.13 | |  |
| lysoPC a C17:0 | 0.92  0.70-1.06 | | 0.95  0.87-1.13 | | 0.97  0.80-1.12 | | 0.75***^###^  0.64-0.86 | | **Treatment** | | 4.32 | | 0.05 | |  |
|  |  |  |  |  |  |  |  |  | **Time** | | 6.87 | | 0.01 | |  |
|  |  |  |  |  |  |  |  |  | **Treatment X time** | | 15.56 | | 0.0004 | |  |
| lysoPC a C18:0 | 2.20  2.13-2.34 | | 2.25  2.16-2.34 | | 2.24  2.12-2.35 | | 2.30  2.22-2.46 | | **Treatment** | | 4.89 | | 0.03 | |  |
|  |  |  |  |  |  |  |  |  | **Time** | | 2.32 | | 0.14 | |  |
|  |  |  |  |  |  |  |  |  | **Treatment X time** | | 1.56 | | 0.22 | |  |
| lysoPC a C18:1 | 1.72  1.44-1.93 | | 1.82  1.64-1.89 | | 1.79  1.69-2.12 | | 1.71  1.54-1.88 | | **Treatment** | | 1.36 | | 0.25 | |  |
|  |  |  |  |  |  |  |  |  | **Time** | | 0.31 | | 0.58 | |  |
|  |  |  |  |  |  |  |  |  | **Treatment X time** | | 6.37 | | 0.02 | |  |
| lysoPC a C18:2 | 2.19  2.06-2.31 | | 2.15  2.03-2.24 | | 2.28  2.07-2.50 | | 2.10**  1.87-2.18 | | **Treatment** | | 7.96 | | 0.008 | |  |
|  |  |  |  |  |  |  |  |  | **Time** | | 0.84 | | 0.37 | |  |
|  |  |  |  |  |  |  |  |  | **Treatment X time** | | 7.91 | | 0.008 | |  |
| lysoPC a C20:3 | 0.84  0.65-0.98 | | 0.86  0.65-0.96 | | 0.95  1.25-0.69 | | 0.42****^####^  0.32-0.83 | | **Treatment** | | 26.54 | | <0.0001 | |  |
|  |  |  |  |  |  |  |  |  | **Time** | | 7.21 | | 0.01 | |  |
|  |  |  |  |  |  |  |  |  | **Treatment X time** | | 29.50 | | <0.0001 | |  |
| lysoPC a C20:4 | 1.53  1.21-1.67 | | 1.57  1.46-1.70 | | 1.53  1.30-1.78 | | 1.25***^####^  1.07-1.46 | | **Treatment** | | 7.22 | | 0.01 | |  |
|  |  |  |  |  |  |  |  |  | **Time** | | 11.15 | | 0.002 | |  |
|  |  |  |  |  |  |  |  |  | **Treatment X time** | | 17.46 | | 0.0002 | |  |
| lysoPC a C24:0 | 0.00  -0.22-0.18 | | 0.14  -0.11-0.38 | | 0.15  -0.05-0.36 | | 0.21  -0.07-0.41 | | **Treatment** | | 2.23 | | 0.14 | |  |
|  |  |  |  |  |  |  |  |  | **Time** | | 2.14 | | 0.15 | |  |
|  |  |  |  |  |  |  |  |  | **Treatment X time** | | 1.16 | | 0.29 | |  |
| lysoPC a C26:0 | -0.10  -0.31-0.29 | | 0.22  -0.13-0.60 | | 0.34^@^  -0.05-0.70 | | 0.07  -0.36-0.32 | | **Treatment** | | 0.001 | | 0.97 | |  |
|  |  |  |  |  |  |  |  |  | **Time** | | 1.13 | | 0.30 | |  |
|  |  |  |  |  |  |  |  |  | **Treatment X time** | | 9.62 | | 0.004 | |  |
| lysoPC a C26:1 | -0.47  -0.70-(-0.19) | | -0.20  -0.67-0.10 | | -0.17  -0.46-0.19 | | -0.41  -0.70-(-0.13) | | **Treatment** | | 0.03 | | 0.87 | |  |
|  |  |  |  |  |  |  |  |  | **Time** | | 0.83 | | 0.37 | |  |
|  |  |  |  |  |  |  |  |  | **Treatment X time** | | 5.36 | | 0.03 | |  |
| lysoPC a C28:0 | -0.35  -0.51-(-0.08) | | -0.12  -0.47-0.28 | | 0.03  -0.34-0.33 | | -0.13  -0.59-0.13 | | **Treatment** | | 0.01 | | 0.92 | |  |
|  |  |  |  |  |  |  |  |  | **Time** | | 1.61 | | 0.21 | |  |
|  |  |  |  |  |  |  |  |  | **Treatment X time** | | 6.13 | | 0.02 | |  |
| lysoPC a C28:1 | -0.42  -0.65-(-0.18) | | -0.20  -0.59-0.07 | | -0.15  -0.49-0.20 | | -0.33  -0.85-(-0.01) | | **Treatment** | | 0.02 | | 0.88 | |  |
|  |  |  |  |  |  |  |  |  | **Time** | | 0.57 | | 0.46 | |  |
|  |  |  |  |  |  |  |  |  | **Treatment X time** | | 5.12 | | 0.03 | |  |
| ***Phosphatidylcholine diacyls*** | | | | | | | | | | | | | | |  |
| PC aa C24:0 | -0.30  -0.48-0.06 | | -0.07  -0.46-0.35 | | 0.03  -0.38-0.24 | | -0.10  -0.55-0.08 | | **Treatment** | | 0.41 | | 0.52 | |  |
|  |  |  |  |  |  |  |  |  | **Time** | | 0.29 | | 0.59 | |  |
|  |  |  |  |  |  |  |  |  | **Treatment X time** | | 4.17 | | 0.05 | |  |
| PC aa C26:0 | 0.34  0.11-0.69 | | 0.49  0.10-0.93 | | 0.75^@^  0.41-1.08 | | 0.57  0.10-0.84 | | **Treatment** | | 0.02 | | 0.89 | |  |
|  |  |  |  |  |  |  |  |  | **Time** | | 4.02 | | 0.05 | |  |
|  |  |  |  |  |  |  |  |  | **Treatment X time** | | 5.02 | | 0.03 | |  |
| PC aa C28:1 | -0.23  -0.49-0.08 | | -0.03  -0.42-0.35 | | 0.12^@^  -0.24-0.44 | | 0.03  -0.34-0.22 | | **Treatment** | | 0.45 | | 0.51 | |  |
|  |  |  |  |  |  |  |  |  | **Time** | | 4.44 | | 0.04 | |  |
|  |  |  |  |  |  |  |  |  | **Treatment X time** | | 4.39 | | 0.04 | |  |
| PC aa C30:0 | 0.17  -0.05-0.39 | | 0.24  -0.13-0.58 | | 0.18  0.09-0.44 | | 0.25  0.16-0.43 | | **Treatment** | | 0.47 | | 0.50 | |  |
|  |  |  |  |  |  |  |  |  | **Time** | | 0.52 | | 0.48 | |  |
|  |  |  |  |  |  |  |  |  | **Treatment X time** | | 0.03 | | 0.86 | |  |
| PC aa C32:0 | 1.17  1.01-1.23 | | 1.18  0.99-1.45 | | 1.06  1.00-1.28 | | 1.11  1.01-1.37 | | **Treatment** | | 1.49 | | 0.23 | |  |
|  |  |  |  |  |  |  |  |  | **Time** | | 0.93 | | 0.34 | |  |
|  |  |  |  |  |  |  |  |  | **Treatment X time** | | 0.001 | | 0.97 | |  |
| PC aa C32:1 | 0.62  0.50-0.80 | | 0.66  0.42-1.06 | | 0.64  0.47-0.97 | | 0.58  0.41-0.82 | | **Treatment** | | 0.01 | | 0.91 | |  |
|  |  |  |  |  |  |  |  |  | **Time** | | 0.26 | | 0.61 | |  |
|  |  |  |  |  |  |  |  |  | **Treatment X time** | | 0.62 | | 0.44 | |  |
| PC aa C32:2 | -0.31  -0.54-(-0.09) | | -0.23  -0.47-(-0.09) | | -0.27  -0.34-0.00 | | -0.28  -0.46-(-0.12) | | **Treatment** | | 0.12 | | 0.73 | |  |
|  |  |  |  |  |  |  |  |  | **Time** | | 0.005 | | 0.95 | |  |
|  |  |  |  |  |  |  |  |  | **Treatment X time** | | 3.33 | | 0.08 | |  |
| PC aa C32:3 | -1.01  -1.17-(-0.79) | | -0.85  -1.11-(-0.70) | | -0.86  -1.14-(-0.64) | | -0.72*^#^  -0.76-(-0.66) | | **Treatment** | | 11.86 | | 0.002 | |  |
|  |  |  |  |  |  |  |  |  | **Time** | | 12.77 | | 0.001 | |  |
|  |  |  |  |  |  |  |  |  | **Treatment X time** | | 0.16 | | 0.69 | |  |
| PC aa C34:1 | 1.84  1.69-1.98 | | 1.84  1.75-1.99 | | 1.90  1.62-2.11 | | 1.94  1.84-2.03 | | **Treatment** | | 0.77 | | 0.39 | |  |
|  |  |  |  |  |  |  |  |  | **Time** | | 2.55 | | 0.12 | |  |
|  |  |  |  |  |  |  |  |  | **Treatment X time** | | 0.95 | | 0.34 | |  |
| PC aa C34:2 | 2.45  2.36-2.55 | | 2.42  2.35-2.47 | | 2.50  2.35-2.56 | | 2.51^##^  2.47-2.62 | | **Treatment** | | 0.40 | | 0.53 | |  |
|  |  |  |  |  |  |  |  |  | **Time** | | 12.77 | | 0.001 | |  |
|  |  |  |  |  |  |  |  |  | **Treatment X time** | | 4.05 | | 0.05 | |  |
| PC aa C34:3 | 0.75  0.59-0.91 | | 0.84  0.61-0.89 | | 0.72  0.66-0.95 | | 0.94**  0.78-1.11 | | **Treatment** | | 12.81 | | 0.001 | |  |
|  |  |  |  |  |  |  |  |  | **Time** | | 2.24 | | 0.14 | |  |
|  |  |  |  |  |  |  |  |  | **Treatment X time** | | 5.06 | | 0.03 | |  |
| PC aa C34:4 | -0.71  -0.85-(-0.66) | | -0.61  -0.81-(-0.55) | | -0.68  -0.79-(-0.45) | | -0.70  -0.79-(-0.56) | | **Treatment** | | 1.99 | | 0.17 | |  |
|  |  |  |  |  |  |  |  |  | **Time** | | 0.01 | | 0.92 | |  |
|  |  |  |  |  |  |  |  |  | **Treatment X time** | | 3.46 | | 0.07 | |  |
| PC aa C36:0 | 0.38  0.32-0.50 | | 0.43  0.33-0.51 | | 0.43  0.27-0.51 | | 0.40  0.35-0.52 | | **Treatment** | | 1.53 | | 0.23 | |  |
|  |  |  |  |  |  |  |  |  | **Time** | | 0.27 | | 0.61 | |  |
|  |  |  |  |  |  |  |  |  | **Treatment X time** | | 0.36 | | 0.55 | |  |
| PC aa C36:1 | 1.11  0.99-1.14 | | 1.08  1.02-1.15 | | 1.05  0.94-1.17 | | 1.42****^####^  1.33-1.50 | | **Treatment** | | 69.84 | | <0.0001 | |  |
|  |  |  |  |  |  |  |  |  | **Time** | | 58.15 | | <0.0001 | |  |
|  |  |  |  |  |  |  |  |  | **Treatment X time** | | 68.80 | | <0.0001 | |  |
| PC aa C36:2 | 2.22  2.08-2.31 | | 2.18  2.12-2.28 | | 2.30  2.13-2.36 | | 2.39**^####^  2.26-2.47 | | **Treatment** | | 2.97 | | 0.09 | |  |
|  |  |  |  |  |  |  |  |  | **Time** | | 33.99 | | <0.0001 | |  |
|  |  |  |  |  |  |  |  |  | **Treatment X time** | | 9.29 | | 0.005 | |  |
| PC aa C36:3 | 1.65  1.55-1.82 | | 1.71  1.60-1.77 | | 1.77  1.69-1.93 | | 1.69  1.55-1.81 | | **Treatment** | | 1.53 | | 0.23 | |  |
|  |  |  |  |  |  |  |  |  | **Time** | | 3.42 | | 0.07 | |  |
|  |  |  |  |  |  |  |  |  | **Treatment X time** | | 2.60 | | 0.12 | |  |
| PC aa C36:4 | 2.00  1.86-2.05 | | 1.99  1.86-2.03 | | 1.93  1.79-2.05 | | 1.85^#^  1.80-2.00 | | **Treatment** | | 2.13 | | 0.15 | |  |
|  |  |  |  |  |  |  |  |  | **Time** | | 8.56 | | 0.006 | |  |
|  |  |  |  |  |  |  |  |  | **Treatment X time** | | 1.28 | | 0.27 | |  |
| PC aa C36:5 | 0.49  0.27-0.66 | | 0.53  0.28-0.56 | | 0.55  0.44-0.80 | | 0.47  0.39-0.61 | | **Treatment** | | 1.80 | | 0.19 | |  |
|  |  |  |  |  |  |  |  |  | **Time** | | 1.17 | | 0.29 | |  |
|  |  |  |  |  |  |  |  |  | **Treatment X time** | | 2.49 | | 0.12 | |  |
| PC aa C36:6 | -0.74  -0.94-(-0.57) | | -0.64  -0.81-(-0.57) | | -0.68  -0.74-(-0.40) | | -0.71  -0.78-(-0.57) | | **Treatment** | | 0.41 | | 0.53 | |  |
|  |  |  |  |  |  |  |  |  | **Time** | | 0.52 | | 0.48 | |  |
|  |  |  |  |  |  |  |  |  | **Treatment X time** | | 3.01 | | 0.09 | |  |
| PC aa C38:0 | 0.13  0.08-0.27 | | 0.21  0.12-0.34 | | 0.16  0.02-0.24 | | 0.24  0.14-0.30 | | **Treatment** | | 9.73 | | 0.004 | |  |
|  |  |  |  |  |  |  |  |  | **Time** | | 0.02 | | 0.89 | |  |
|  |  |  |  |  |  |  |  |  | **Treatment X time** | | 0.21 | | 0.65 | |  |
| PC aa C38:1 | -0.11  -0.25-0.14 | | 0.02  -0.13-0.15 | | 0.01  -0.47-0.17 | | 0.17**^#^  0.06-0.41 | | **Treatment** | | 14.04 | | 0.0007 | |  |
|  |  |  |  |  |  |  |  |  | **Time** | | 7.76 | | 0.009 | |  |
|  |  |  |  |  |  |  |  |  | **Treatment X time** | | 1.37 | | 0.25 | |  |
| PC aa C38:3 | 1.20  1.04-1.30 | | 1.22  1.07-1.29 | | 1.22  1.15-1.35 | | 1.21  1.17-1.40 | | **Treatment** | | 0.02 | | 0.90 | |  |
|  |  |  |  |  |  |  |  |  | **Time** | | 1.58 | | 0.22 | |  |
|  |  |  |  |  |  |  |  |  | **Treatment X time** | | 0.09 | | 0.77 | |  |
| PC aa C38:4 | 1.75  1.61-1.82 | | 1.75  1.59-1.86 | | 1.67  1.48-1.83 | | 1.83***  1.75-1.96 | | **Treatment** | | 11.48 | | 0.002 | |  |
|  |  |  |  |  |  |  |  |  | **Time** | | <0.0001 | | 1.00 | |  |
|  |  |  |  |  |  |  |  |  | **Treatment X time** | | 8.91 | | 0.005 | |  |
| PC aa C38:5 | 1.32  1.17-1.43 | | 1.37  1.18-1.44 | | 1.26  1.04-1.50 | | 1.26  1.15-1.38 | | **Treatment** | | 0.02 | | 0.89 | |  |
|  |  |  |  |  |  |  |  |  | **Time** | | 2.77 | | 0.11 | |  |
|  |  |  |  |  |  |  |  |  | **Treatment X time** | | 0.45 | | 0.51 | |  |
| PC aa C38:6 | 1.97  1.82-2.01 | | 1.97  1.83-2.02 | | 1.85  1.74-1.95 | | 2.01****^#^  2.00-2.12 | | **Treatment** | | 20.47 | | <0.0001 | |  |
|  |  |  |  |  |  |  |  |  | **Time** | | <0.0001 | | 1.00 | |  |
|  |  |  |  |  |  |  |  |  | **Treatment X time** | | 15.33 | | 0.0004 | |  |
| PC aa C40:2 | -0.25  -0.50-(-0.22) | | -0.22  -0.38-(-0.12) | | -0.24  -0.35-(-0.14) | | -0.42**^##^  -0.57-(-0.13) | | **Treatment** | | 1.86 | | 0.18 | |  |
|  |  |  |  |  |  |  |  |  | **Time** | | 3.90 | | 0.06 | |  |
|  |  |  |  |  |  |  |  |  | **Treatment X time** | | 12.15 | | 0.001 | |  |
| PC aa C40:3 | -0.15  -0.34-(-0.09) | | -0.12  -0.28-(-0.03) | | -0.19  -0.24-0.00 | | -0.31**^###^  -0.45-(-0.04) | | **Treatment** | | 3.04 | | 0.09 | |  |
|  |  |  |  |  |  |  |  |  | **Time** | | 8.02 | | 0.008 | |  |
|  |  |  |  |  |  |  |  |  | **Treatment X time** | | 10.51 | | 0.003 | |  |
| PC aa C40:4 | 0.40  0.19-0.42 | | 0.39  0.22-0.47 | | 0.30  0.15-0.44 | | 0.38  0.26-0.55 | | **Treatment** | | 3.56 | | 0.07 | |  |
|  |  |  |  |  |  |  |  |  | **Time** | | 0.22 | | 0.64 | |  |
|  |  |  |  |  |  |  |  |  | **Treatment X time** | | 0.71 | | 0.41 | |  |
| PC aa C40:5 | 0.56  0.39-0.67 | | 0.60  0.42-0.64 | | 0.52  0.33-0.67 | | 0.65***^#^  0.56-0.86 | | **Treatment** | | 10.75 | | 0.002 | |  |
|  |  |  |  |  |  |  |  |  | **Time** | | 1.68 | | 0.20 | |  |
|  |  |  |  |  |  |  |  |  | **Treatment X time** | | 7.61 | | 0.009 | |  |
| PC aa C40:6 | 1.47  1.30-1.51 | | 1.42  1.34-1.51 | | 1.37  1.22-1.53 | | 1.77****^####^  1.70-1.85 | | **Treatment** | | 77.72 | | <0.0001 | |  |
|  |  |  |  |  |  |  |  |  | **Time** | | 42.31 | | <0.0001 | |  |
|  |  |  |  |  |  |  |  |  | **Treatment X time** | | 80.19 | | <0.0001 | |  |
| PC aa C42:0 | -0.80  -0.92-(-0.73) | | -0.68*  -0.78-(-0.59) | | -0.75  -0.87-(-0.63) | | -0.77  -0.89-(-0.63) | | **Treatment** | | 4.21 | | 0.05 | |  |
|  |  |  |  |  |  |  |  |  | **Time** | | 0.42 | | 0.52 | |  |
|  |  |  |  |  |  |  |  |  | **Treatment X time** | | 7.25 | | 0.01 | |  |
| PC aa C42:1 | -0.74  -1.02-(-0.66) | | -0.69  -0.80-(-0.63) | | -0.78  -0.95-(-0.69) | | -0.75  -0.80-(-0.62) | | **Treatment** | | 7.78 | | 0.009 | |  |
|  |  |  |  |  |  |  |  |  | **Time** | | 0.95 | | 0.34 | |  |
|  |  |  |  |  |  |  |  |  | **Treatment X time** | | 0.02 | | 0.88 | |  |
| PC aa C42:2 | -0.54  -0.68-(-0.44) | | -0.52  -0.61-(-0.39) | | -0.52  -0.61-(-0.41) | | -0.61*^##^  -0.77-(-0.48) | | **Treatment** | | 1.68 | | 0.20 | |  |
|  |  |  |  |  |  |  |  |  | **Time** | | 3.25 | | 0.08 | |  |
|  |  |  |  |  |  |  |  |  | **Treatment X time** | | 10.65 | | 0.003 | |  |
| PC aa C42:4 | -0.59  -0.81-(-0.44) | | -0.61  -0.71-(-0.47) | | -0.62  -0.74-(-0.50) | | -0.47**^#^  -0.56-(-0.37) | | **Treatment** | | 9.15 | | 0.005 | |  |
|  |  |  |  |  |  |  |  |  | **Time** | | 5.64 | | 0.02 | |  |
|  |  |  |  |  |  |  |  |  | **Treatment X time** | | 5.51 | | 0.02 | |  |
| PC aa C42:5 | -0.44  -0.67-(-0.41) | | -0.45  -0.62-(-0.37) | | -0.56  -0.68-(-0.45) | | -0.41***  -0.50-(-0.24) | | **Treatment** | | 12.43 | | 0.001 | |  |
|  |  |  |  |  |  |  |  |  | **Time** | | 0.09 | | 0.76 | |  |
|  |  |  |  |  |  |  |  |  | **Treatment X time** | | 6.12 | | 0.02 | |  |
| PC aa C42:6 | -0.02  -0.23-0.08 | | -0.02  -0.13-0.09 | | -0.07  -0.23-0.03 | | -0.06  -0.21-0.15 | | **Treatment** | | 0.06 | | 0.81 | |  |
|  |  |  |  |  |  |  |  |  | **Time** | | 1.62 | | 0.21 | |  |
|  |  |  |  |  |  |  |  |  | **Treatment X time** | | 0.10 | | 0.76 | |  |
| ***Phosphatidylcholine acyl-alkyls*** | | | | | | | | | | | | | | |  |
| PC ae C30:1 | -0.43  -0.88-(-0.05) | | -0.24  -0.96-0.20 | | -0.11  -0.46-0.27 | | -0.25  -0.63-(-0.01) | | **Treatment** | | 0.02 | | 0.90 | |  |
|  |  |  |  |  |  |  |  |  | **Time** | | 2.59 | | 0.12 | |  |
|  |  |  |  |  |  |  |  |  | **Treatment X time** | | 3.14 | | 0.09 | |  |
| PC ae C30:2 | -0.92  -1.17-(-0.67) | | -0.72  -1.03-(-0.45) | | -0.73  -1.05-(-0.49) | | -0.69  -0.98-(-0.47) | | **Treatment** | | 3.20 | | 0.08 | |  |
|  |  |  |  |  |  |  |  |  | **Time** | | 2.11 | | 0.16 | |  |
|  |  |  |  |  |  |  |  |  | **Treatment X time** | | 1.12 | | 0.30 | |  |
| PC ae C32:1 | -0.25  -0.44-(-0.20) | | -0.20  -0.35-0.06 | | -0.15  -0.35-0.09 | | -0.14  -0.27-(-0.02) | | **Treatment** | | 1.29 | | 0.26 | |  |
|  |  |  |  |  |  |  |  |  | **Time** | | 4.26 | | 0.05 | |  |
|  |  |  |  |  |  |  |  |  | **Treatment X time** | | 1.11 | | 0.30 | |  |
| PC ae C32:2 | -0.61  -0.76-(-0.55) | | -0.56  -0.78-(-0.32) | | -0.50  -0.71-(-0.23) | | -0.60  -0.84-(-0.46) | | **Treatment** | | 0.12 | | 0.73 | |  |
|  |  |  |  |  |  |  |  |  | **Time** | | 0.40 | | 0.53 | |  |
|  |  |  |  |  |  |  |  |  | **Treatment X time** | | 4.68 | | 0.04 | |  |
| PC ae C34:0 | -0.32  -0.46-(-0.22) | | -0.25  -0.42-(-0.08) | | -0.36  -0.42-(-0.19) | | -0.36  -0.47-(-0.25) | | **Treatment** | | 1.05 | | 0.31 | |  |
|  |  |  |  |  |  |  |  |  | **Time** | | 2.78 | | 0.11 | |  |
|  |  |  |  |  |  |  |  |  | **Treatment X time** | | 3.89 | | 0.06 | |  |
| PC ae C34:1 | 0.24  0.16-0.36 | | 0.28  0.21-0.39 | | 0.28  0.26-0.45 | | 0.29  0.24-0.35 | | **Treatment** | | 0.74 | | 0.40 | |  |
|  |  |  |  |  |  |  |  |  | **Time** | | 1.54 | | 0.22 | |  |
|  |  |  |  |  |  |  |  |  | **Treatment X time** | | 1.23 | | 0.28 | |  |
| PC ae C34:2 | 0.39  0.26-0.51 | | 0.43  0.29-0.53 | | 0.41  0.19-0.54 | | 0.38  0.27-0.45 | | **Treatment** | | 0.23 | | 0.63 | |  |
|  |  |  |  |  |  |  |  |  | **Time** | | 0.51 | | 0.48 | |  |
|  |  |  |  |  |  |  |  |  | **Treatment X time** | | 1.47 | | 0.23 | |  |
| PC ae C34:3 | -0.16  -0.21-0.02 | | -0.11  -0.21-0.01 | | -0.12  -0.22-0.06 | | -0.20  -0.28-(-0.02) | | **Treatment** | | 1.06 | | 0.31 | |  |
|  |  |  |  |  |  |  |  |  | **Time** | | 0.68 | | 0.42 | |  |
|  |  |  |  |  |  |  |  |  | **Treatment X time** | | 3.65 | | 0.06 | |  |
| PC ae C36:0 | -0.33 -0.40-(-0.23) | | -0.28  -0.40-(-0.11) | | -0.30  -0.37-(-0.24) | | -0.32  -0.40-(-0.24) | | **Treatment** | | 0.45 | | 0.51 | |  |
|  |  |  |  |  |  |  |  |  | **Time** | | 0.08 | | 0.78 | |  |
|  |  |  |  |  |  |  |  |  | **Treatment X time** | | 1.85 | | 0.18 | |  |
| PC ae C36:1 | 0.46  0.29-0.54 | | 0.49  0.40-0.62 | | 0.51  0.42-0.64 | | 0.51  0.43-0.55 | | **Treatment** | | 0.60 | | 0.44 | |  |
|  |  |  |  |  |  |  |  |  | **Time** | | 2.09 | | 0.16 | |  |
|  |  |  |  |  |  |  |  |  | **Treatment X time** | | 1.39 | | 0.25 | |  |
| PC ae C36:2 | 0.95  0.80-1.08 | | 0.92  0.90-1.05 | | 1.03  0.81-1.12 | | 0.86**^#^  0.68-0.97 | | **Treatment** | | 8.17 | | 0.007 | |  |
|  |  |  |  |  |  |  |  |  | **Time** | | 1.20 | | 0.28 | |  |
|  |  |  |  |  |  |  |  |  | **Treatment X time** | | 8.34 | | 0.007 | |  |
| PC ae C36:3 | 0.07  -0.04-0.24 | | 0.12  0.01-0.25 | | 0.13  -0.06-0.23 | | 0.14  0.04-0.26 | | **Treatment** | | 0.53 | | 0.47 | |  |
|  |  |  |  |  |  |  |  |  | **Time** | | 1.16 | | 0.29 | |  |
|  |  |  |  |  |  |  |  |  | **Treatment X time** | | 0.07 | | 0.79 | |  |
| PC ae C36:4 | 0.33  0.18-0.39 | | 0.33  0.18-0.41 | | 0.29  0.14-0.41 | | 0.20*^###^  0.12-0.25 | | **Treatment** | | 3.12 | | 0.09 | |  |
|  |  |  |  |  |  |  |  |  | **Time** | | 15.29 | | 0.0004 | |  |
|  |  |  |  |  |  |  |  |  | **Treatment X time** | | 4.37 | | 0.04 | |  |
| PC ae C36:5 | 0.10  0.07-0.23 | | 0.11  0.00-0.16 | | 0.089  -0.12-0.26 | | 0.14  0.06-0.29 | | **Treatment** | | 1.78 | | 0.19 | |  |
|  |  |  |  |  |  |  |  |  | **Time** | | 0.06 | | 0.81 | |  |
|  |  |  |  |  |  |  |  |  | **Treatment X time** | | 4.67 | | 0.04 | |  |
| PC ae C38:0 | 0.33  0.20-0.48 | | 0.37  0.12-0.56 | | 0.21  0.11-0.40 | | 0.36  0.21-0.62 | | **Treatment** | | 5.22 | | 0.03 | |  |
|  |  |  |  |  |  |  |  |  | **Time** | | 0.26 | | 0.61 | |  |
|  |  |  |  |  |  |  |  |  | **Treatment X time** | | 2.46 | | 0.13 | |  |
| PC ae C38:1 | 0.01  -0.14-0.07 | | 0.02  -0.11-0.17 | | 0.08  -0.08-0.25 | | 0.04  -0.25-0.20 | | **Treatment** | | 0.11 | | 0.74 | |  |
|  |  |  |  |  |  |  |  |  | **Time** | | 0.57 | | 0.46 | |  |
|  |  |  |  |  |  |  |  |  | **Treatment X time** | | 2.71 | | 0.11 | |  |
| PC ae C38:2 | 0.87  0.70-1.09 | | 0.85  0.78-1.01 | | 0.99  0.81-1.09 | | 0.57****^####^  0.43-0.75 | | **Treatment** | | 38.85 | | <0.0001 | |  |
|  |  |  |  |  |  |  |  |  | **Time** | | 10.31 | | 0.003 | |  |
|  |  |  |  |  |  |  |  |  | **Treatment X time** | | 36.08 | | <0.0001 | |  |
| PC ae C38:3 | 0.21  0.06-0.33 | | 0.26  0.16-0.38 | | 0.30  0.17-0.43 | | 0.14***^##^  0.04-0.27 | | **Treatment** | | 4.54 | | 0.04 | |  |
|  |  |  |  |  |  |  |  |  | **Time** | | 1.08 | | 0.31 | |  |
|  |  |  |  |  |  |  |  |  | **Treatment X time** | | 15.06 | | 0.0005 | |  |
| PC ae C38:4 | 0.57  0.39-0.64 | | 0.56  0.46-0.69 | | 0.52  0.29-0.66 | | 0.39^##^  0.30-0.53 | | **Treatment** | | 1.30 | | 0.26 | |  |
|  |  |  |  |  |  |  |  |  | **Time** | | 13.03 | | 0.001 | |  |
|  |  |  |  |  |  |  |  |  | **Treatment X time** | | 3.95 | | 0.06 | |  |
| PC ae C38:5 | 0.27  0.16-0.37 | | 0.30  0.18-0.36 | | 0.28  0.09-0.31 | | 0.11**^###^  0.06-0.22 | | **Treatment** | | 4.41 | | 0.04 | |  |
|  |  |  |  |  |  |  |  |  | **Time** | | 15.08 | | 0.0005 | |  |
|  |  |  |  |  |  |  |  |  | **Treatment X time** | | 8.56 | | 0.006 | |  |
| PC ae C38:6 | 0.22  0.04-0.27 | | 0.22  0.04-0.33 | | 0.18  0.05-0.27 | | 0.31**  0.21-0.38 | | **Treatment** | | 9.63 | | 0.004 | |  |
|  |  |  |  |  |  |  |  |  | **Time** | | 2.86 | | 0.10 | |  |
|  |  |  |  |  |  |  |  |  | **Treatment X time** | | 4.49 | | 0.04 | |  |
| PC ae C40:1 | 0.32  0.19-0.37 | | 0.36  0.27-0.47 | | 0.31  0.20-0.42 | | 0.23^##^  0.15-0.32 | | **Treatment** | | 0.01 | | 0.91 | |  |
|  |  |  |  |  |  |  |  |  | **Time** | | 10.29 | | 0.003 | |  |
|  |  |  |  |  |  |  |  |  | **Treatment X time** | | 7.12 | | 0.01 | |  |
| PC ae C40:2 | -0.21  -0.36-(-0.09) | | -0.15  -0.23-(-0.01) | | -0.19  -0.29-(-0.06) | | -0.25^#^  -0.29-(-0.12) | | **Treatment** | | 0.45 | | 0.51 | |  |
|  |  |  |  |  |  |  |  |  | **Time** | | 1.85 | | 0.18 | |  |
|  |  |  |  |  |  |  |  |  | **Treatment X time** | | 7.26 | | 0.01 | |  |
| PC ae C40:3 | -0.16  -0.22-(-0.11) | | -0.12  -0.20-0.02 | | -0.11  -0.25-(-0.03) | | -0.28**^##^  -0.31-(-0.04) | | **Treatment** | | 1.36 | | 0.25 | |  |
|  |  |  |  |  |  |  |  |  | **Time** | | 3.96 | | 0.05 | |  |
|  |  |  |  |  |  |  |  |  | **Treatment X time** | | 15.23 | | 0.0004 | |  |
| PC ae C40:4 | 0.29  0.10-0.40 | | 0.32  0.20-0.49 | | 0.28  0.04-0.38 | | 0.12^###^  0.06-0.23 | | **Treatment** | | 0.60 | | 0.44 | |  |
|  |  |  |  |  |  |  |  |  | **Time** | | 13.64 | | 0.0008 | |  |
|  |  |  |  |  |  |  |  |  | **Treatment X time** | | 6.31 | | 0.02 | |  |
| PC ae C40:5 | 0.02  -0.06-0.11 | | 0.06  -0.01-0.18 | | 0.01  -0.04-0.11 | | 0.04  0.01-0.16 | | **Treatment** | | 6.22 | | 0.02 | |  |
|  |  |  |  |  |  |  |  |  | **Time** | | 0.002 | | 0.96 | |  |
|  |  |  |  |  |  |  |  |  | **Treatment X time** | | 0.03 | | 0.86 | |  |
| PC ae C40:6 | 0.41  0.24-0.47 | | 0.41  0.34-0.55 | | 0.37  0.11-0.52 | | 0.45  0.34-0.57 | | **Treatment** | | 5.96 | | 0.02 | |  |
|  |  |  |  |  |  |  |  |  | **Time** | | 0.11 | | 0.75 | |  |
|  |  |  |  |  |  |  |  |  | **Treatment X time** | | 0.90 | | 0.35 | |  |
| PC ae C42:1 | -0.22  -0.37-(-0.16) | | -0.15  -0.33-0.01 | | -0.21  -0.38-(-0.03) | | -0.10  -0.20-0.05 | | **Treatment** | | 11.04 | | 0.002 | |  |
|  |  |  |  |  |  |  |  |  | **Time** | | 2.95 | | 0.10 | |  |
|  |  |  |  |  |  |  |  |  | **Treatment X time** | | 0.27 | | 0.61 | |  |
| PC ae C42:2 | -0.41  -0.52-(-0.37) | | -0.39  -0.46-(-0.22) | | -0.41  -0.60-(-0.28) | | -0.51^#^  -0.68-(-0.29) | | **Treatment** | | 0.23 | | 0.64 | |  |
|  |  |  |  |  |  |  |  |  | **Time** | | 4.83 | | 0.04 | |  |
|  |  |  |  |  |  |  |  |  | **Treatment X time** | | 6.00 | | 0.02 | |  |
| PC ae C42:3 | -0.10  -0.31-(-0.05) | | -0.08  -0.16-0.10 | | -0.11  -0.24-0.12 | | -0.11  -0.38-(-0.03) | | **Treatment** | | 0.003 | | 0.96 | |  |
|  |  |  |  |  |  |  |  |  | **Time** | | 1.46 | | 0.24 | |  |
|  |  |  |  |  |  |  |  |  | **Treatment X time** | | 3.18 | | 0.08 | |  |
| PC ae C44:3 | -0.82  1.10-(-0.70) | | -0.77  -1.01-(-0.58) | | -0.73  -1.03-(-0.49) | | -0.70  -0.91-(-0.40) | | **Treatment** | | 1.82 | | 0.19 | |  |
|  |  |  |  |  |  |  |  |  | **Time** | | 4.34 | | 0.05 | |  |
|  |  |  |  |  |  |  |  |  | **Treatment X time** | | 0.01 | | 0.91 | |  |
| PC ae C44:4 | -0.88  -1.02-(-0.80) | | -0.82  -0.95-(-0.61) | | -0.81  -1.05-(-0.58) | | -0.78  -0.90-(-0.65) | | **Treatment** | | 3.06 | | 0.09 | |  |
|  |  |  |  |  |  |  |  |  | **Time** | | 1.71 | | 0.20 | |  |
|  |  |  |  |  |  |  |  |  | **Treatment X time** | | 0.39 | | 0.54 | |  |
| PC ae C44:6 | -0.86  -1.01-(-0.73) | | -0.86  -0.97-(-0.72) | | -0.89  -0.94-(-0.77) | | -0.84  -0.91-(-0.77) | | **Treatment** | | 0.86 | | 0.36 | |  |
|  |  |  |  |  |  |  |  |  | **Time** | | 0.65 | | 0.43 | |  |
|  |  |  |  |  |  |  |  |  | **Treatment X time** | | 0.02 | | 0.88 | |  |
| **Sphingolipids** | | | | | | | | | | | | | | |  |
| SM(OH) C14:1 | -0.30  -0.48-(-0.23) | | -0.32  -0.39-(-0.14) | | -0.30  -0.38-(-0.19) | | -0.05****^####^  -0.15-(0.08) | | **Treatment** | | 25.14 | | <0.0001 | |  |
|  |  |  |  |  |  |  |  |  | **Time** | | 30.46 | | <0.0001 | |  |
|  |  |  |  |  |  |  |  |  | **Treatment X time** | | 14.74 | | 0.0005 | |  |
| SM(OH) C16:1 | -1.09  -1.23-(-0.84) | | -0.94  -1.01-(-0.83) | | -1.00  -1.09-(-0.82) | | -0.79****^##^  -0.88-(-0.53) | | **Treatment** | | 34.42 | | <0.0001 | |  |
|  |  |  |  |  |  |  |  |  | **Time** | | 11.83 | | 0.002 | |  |
|  |  |  |  |  |  |  |  |  | **Treatment X time** | | 2.39 | | 0.13 | |  |
| SM(OH) C22:1 | 0.19  0.16-0.28 | | 0.21  0.18-0.32 | | 0.07^@^  -0.04-0.21 | | 0.52****^####^  0.41-0.58 | | **Treatment** | | 135.60 | | <0.0001 | |  |
|  |  |  |  |  |  |  |  |  | **Time** | | 15.48 | | 0.0004 | |  |
|  |  |  |  |  |  |  |  |  | **Treatment X time** | | 100.05 | | <0.0001 | |  |
| SM(OH) C22:2 | -0.05  -0.12-0.11 | | 0.02  -0.06-0.12 | | -0.11  -0.20-(-0.03) | | 0.18****^####^  0.12-0.21 | | **Treatment** | | 64.27 | | <0.0001 | |  |
|  |  |  |  |  |  |  |  |  | **Time** | | 2.83 | | 0.10 | |  |
|  |  |  |  |  |  |  |  |  | **Treatment X time** | | 29.63 | | <0.0001 | |  |
| SM(OH) C24:1 | -0.70  -0.79-(-0.61) | | -0.68  -0.85-(-0.52) | | -0.79  -0.87-(-0.64) | | -0.46****^####^  -0.56-(-0.30) | | **Treatment** | | 44.37 | | <0.0001 | |  |
|  |  |  |  |  |  |  |  |  | **Time** | | 5.26 | | 0.03 | |  |
|  |  |  |  |  |  |  |  |  | **Treatment X time** | | 29.49 | | <0.0001 | |  |
| SM C16:0 | 0.75  0.74-0.82 | | 0.80  0.71-0.86 | | 0.75  0.69-0.87 | | 1.18****^####^  1.14-1.32 | | **Treatment** | | 185.43 | | <0.0001 | |  |
|  |  |  |  |  |  |  |  |  | **Time** | | 151.75 | | <0.0001 | |  |
|  |  |  |  |  |  |  |  |  | **Treatment X time** | | 146.46 | | <0.0001 | |  |
| SM C16:1 | 0.16  0.08-0.22 | | 0.19  0.11-0.26 | | 0.12  0.04-0.22 | | 0.40****^####^  0.34-0.50 | | **Treatment** | | 74.70 | | <0.0001 | |  |
|  |  |  |  |  |  |  |  |  | **Time** | | 33.97 | | <0.0001 | |  |
|  |  |  |  |  |  |  |  |  | **Treatment X time** | | 49.59 | | <0.0001 | |  |
| SM C18:0 | -0.20  -0.27-(-0.17) | | -0.22  -0.30-(-0.13) | | -0.31  -0.39-(-0.11) | | 0.20****^####^  -0.05-0.44 | | **Treatment** | | 67.52 | | <0.0001 | |  |
|  |  |  |  |  |  |  |  |  | **Time** | | 33.85 | | <0.0001 | |  |
|  |  |  |  |  |  |  |  |  | **Treatment X time** | | 68.58 | | <0.0001 | |  |
| SM C18:1 | -0.70  -0.81-(-0.57) | | -0.67  -0.76-(-0.60) | | -0.74  -0.82-(-0.57) | | -0.32****^####^  -0.46-(-0.17) | | **Treatment** | | 60.94 | | <0.0001 | |  |
|  |  |  |  |  |  |  |  |  | **Time** | | 40.40 | | <0.0001 | |  |
|  |  |  |  |  |  |  |  |  | **Treatment X time** | | 58.66 | | <0.0001 | |  |
| SM C20:2 | -1.72  -2.05-(-1.48) | | -1.78  -1.92-(-1.37) | | -1.83  -3.00-(-1.49) | | -1.32*  -2.15-(-0.79) | | **Treatment** | | 5.15 | | 0.03 | |  |
|  |  |  |  |  |  |  |  |  | **Time** | | 0.01 | | 0.91 | |  |
|  |  |  |  |  |  |  |  |  | **Treatment X time** | | 4.85 | | 0.04 | |  |
| SM C24:0 | 0.67  0.57-0.74 | | 0.66  0.58-0.72 | | 0.60  0.50-0.68 | | 0.88****^####^  0.77-1.00 | | **Treatment** | | 61.85 | | <0.0001 | |  |
|  |  |  |  |  |  |  |  |  | **Time** | | 14.64 | | 0.0005 | |  |
|  |  |  |  |  |  |  |  |  | **Treatment X time** | | 60.06 | | <0.0001 | |  |
| SM C24:1 | 0.83  0.73-1.00 | | 0.91  0.81-0.97 | | 0.76  0.63-0.85 | | 1.32****^####^  1.25-1.38 | | **Treatment** | | 199.23 | | <0.0001 | |  |
|  |  |  |  |  |  |  |  |  | **Time** | | 63.44 | | <0.0001 | |  |
|  |  |  |  |  |  |  |  |  | **Treatment X time** | | 134.34 | | <0.0001 | |  |
| SM C26:0 | -1.64  -3.00-(-1.27) | | -1.51  -2.05-(-1.26) | | -1.66  -2.70-(-1.41) | | -1.42  -2.15-(-1.15) | | **Treatment** | | 4.38 | | 0.05 | |  |
|  |  |  |  |  |  |  |  |  | **Time** | | 0.15 | | 0.71 | |  |
|  |  |  |  |  |  |  |  |  | **Treatment X time** | | 0.30 | | 0.59 | |  |
| SM C26:1 | -1.54  -2.00-(-1.32) | | -1.52  -2.30-(-1.32) | | -1.60  -2.15-(-1.22) | | -1.24  -1.70-(-1.07) | | **Treatment** | | 2.69 | | 0.11 | |  |
|  |  |  |  |  |  |  |  |  | **Time** | | 2.36 | | 0.14 | |  |
|  |  |  |  |  |  |  |  |  | **Treatment X time** | | 4.00 | | 0.06 | |  |
| **Monosaccharides** | | | | | | | | | | | | | | |  |
| Hexoses (H1) | 3.86  3.68-3.94 | | 3.87  3.72-4.17 | | 3.80  3.68-3.96 | | 3.73  3.61-4.05 | | **Treatment** | | 0.26 | | 0.62 | |  |
|  |  |  |  |  |  |  |  |  | **Time** | | 2.05 | | 0.16 | |  |
|  |  |  |  |  |  |  |  |  | **Treatment X time** | | 0.15 | | 0.70 | |  |
| **Metabolite ratios** | | | | | | | | | | | | | | |  |
| C2/C0 | -0.19  -0.32-0.09 | | -0.30  -0.40-(-0.18) | | -0.20  -0.25-0.10 | | 0.12^###^  -0.32-0.24 | | **Treatment** | | 0.20 | | 0.66 | |  |
|  |  |  |  |  |  |  |  |  | **Time** | | 12.30 | | 0.001 | |  |
|  |  |  |  |  |  |  |  |  | **Treatment X time** | | 9.75 | | 0.004 | |  |
| C3/C0 | -1.55  -1.73-(-1.50) | | -1.73***  -1.80-(-1.68) | | -1.59  -1.66-(-1.47) | | -1.56^####^  -1.67-(-1.47) | | **Treatment** | | 10.15 | | 0.003 | |  |
|  |  |  |  |  |  |  |  |  | **Time** | | 16.28 | | 0.0003 | |  |
|  |  |  |  |  |  |  |  |  | **Treatment X time** | | 15.02 | | 0.0005 | |  |
| C4-/C0 | -1.59  -1.71-(-1.43) | | -1.67  -1.80-(-1.59) | | -1.54  -1.59-(-1.42) | | -1.32*^####^  -1.61-(-1.16) | | **Treatment** | | 0.56 | | 0.46 | |  |
|  |  |  |  |  |  |  |  |  | **Time** | | 28.26 | | <0.0001 | |  |
|  |  |  |  |  |  |  |  |  | **Treatment X time** | | 11.18 | | 0.002 | |  |
| C5-/C0 | -1.80  -1.98-(-1.76) | | -1.98**  -2.08-(-1.88) | | -1.87  -1.97-(-1.74) | | -1.68****^####^  -1.76-(-1.61) | | **Treatment** | | 0.90 | | 0.35 | |  |
|  |  |  |  |  |  |  |  |  | **Time** | | 34.57 | | <0.0001 | |  |
|  |  |  |  |  |  |  |  |  | **Treatment X time** | | 45.40 | | <0.0001 | |  |
| C18/C18:1 | -0.33  -0.45-0.01 | | -0.33  -0.39-(-0.30) | | -0.33  -0.49-(-0.21) | | -0.47  -0.55-(-0.22) | | **Treatment** | | 5.41 | | 0.03 | |  |
|  |  |  |  |  |  |  |  |  | **Time** | | 6.60 | | 0.01 | |  |
|  |  |  |  |  |  |  |  |  | **Treatment X time** | | 0.52 | | 0.48 | |  |
| C16 + C18 | -0.21  -0.27-(-0.11) | | -0.17  -0.25-(-0.10) | | -0.24  -0.36-(-0.11) | | -0.04****^##^  -0.18-0.14) | | **Treatment** | | 22.02 | | <0.0001 | |  |
|  |  |  |  |  |  |  |  |  | **Time** | | 3.78 | | 0.06 | |  |
|  |  |  |  |  |  |  |  |  | **Treatment X time** | | 13.54 | | 0.0008 | |  |
| C14 + C16 + C18 | -0.12  -0.18-(-0.02) | | -0.09  -0.17-(-0.02) | | -0.15  -0.27-(-0.02) | | 0.04****^##^  -0.10-0.23 | | **Treatment** | | 20.09 | | <0.0001 | |  |
|  |  |  |  |  |  |  |  |  | **Time** | | 4.90 | | 0.03 | |  |
|  |  |  |  |  |  |  |  |  | **Treatment X time** | | 13.20 | | 0.0009 | |  |
| C14 + C16 + C18/C0 | -1.64  -1.77-(-1.43) | | -1.57  -1.67-(-1.49) | | -1.66  -1.75-(-1.46) | | -1.24****^###^  -1.54-(-1.00) | | **Treatment** | | 23.11 | | <0.0001 | |  |
|  |  |  |  |  |  |  |  |  | **Time** | | 10.73 | | 0.003 | |  |
|  |  |  |  |  |  |  |  |  | **Treatment X time** | | 11.75 | | 0.002 | |  |
| CRT-1 | -2.14  -2.21-(-1.93) | | -2.06  -2.15-(-1.94) | | -2.20  -2.30-(-1.97) | | -1.84****^##^  -2.07-(-1.52) | | **Treatment** | | 22.69 | | <0.0001 | |  |
|  |  |  |  |  |  |  |  |  | **Time** | | 4.71 | | 0.04 | |  |
|  |  |  |  |  |  |  |  |  | **Treatment X time** | | 11.20 | | 0.002 | |  |
| CPT-1 | -1.72  -1.84-(-1.52) | | -1.65  -1.75-(-1.57) | | -1.75  -1.84-(-1.56) | | -1.33****^###^  -1.63-(-1.09) | | **Treatment** | | 24.53 | | <0.0001 | |  |
|  |  |  |  |  |  |  |  |  | **Time** | | 9.85 | | 0.004 | |  |
|  |  |  |  |  |  |  |  |  | **Treatment X time** | | 12.05 | | 0.002 | |  |
| Hydroxylated LCACs | -0.80  -0.89-(-0.71) | | -0.77  -0.91-(-0.70) | | -0.82  -0.91-(-0.73) | | -0.66****^###^  -0.76-(-0.50) | | **Treatment** | | 16.25 | | 0.0003 | |  |
|  |  |  |  |  |  |  |  |  | **Time** | | 5.98 | | 0.02 | |  |
|  |  |  |  |  |  |  |  |  | **Treatment X time** | | 12.17 | | 0.001 | |  |
| BCAA | 2.83  2.74-3.09 | | 2.76  2.57-3.18 | | 2.82  2.66-3.06 | | 2.94  2.68-3.21 | | **Treatment** | | 0.14 | | 0.71 | |  |
|  |  |  |  |  |  |  |  |  | **Time** | | 0.58 | | 0.45 | |  |
|  |  |  |  |  |  |  |  |  | **Treatment X time** | | 4.08 | | 0.05 | |  |
| AAA | 2.22  2.10-2.64 | | 2.18  1.95-2.74 | | 2.26  2.13-2.60 | | 2.46  2.13-2.79 | | **Treatment** | | 0.40 | | 0.53 | |  |
|  |  |  |  |  |  |  |  |  | **Time** | | 2.45 | | 0.13 | |  |
|  |  |  |  |  |  |  |  |  | **Treatment X time** | | 4.09 | | 0.05 | |  |
| BCAA/AAA | 0.58  0.44-0.64 | | 0.58  0.44-0.69 | | 0.53  0.46-0.59 | | 0.47^#^  0.37-0.55 | | **Treatment** | | 1.16 | | 0.29 | |  |
|  |  |  |  |  |  |  |  |  | **Time** | | 9.56 | | 0.004 | |  |
|  |  |  |  |  |  |  |  |  | **Treatment X time** | | 2.34 | | 0.14 | |  |
| Glucogenic amino acids | 3.46  3.33-3.67 | | 3.36  3.25-3.75 | | 3.42  3.31-3.60 | | 3.55  3.29-3.70 | | **Treatment** | | 0.42 | | 0.52 | |  |
|  |  |  |  |  |  |  |  |  | **Time** | | 0.10 | | 0.75 | |  |
|  |  |  |  |  |  |  |  |  | **Treatment X time** | | 3.25 | | 0.08 | |  |
| Tyrosine/  Phenylalanine | -0.11  -0.19-(-0.03) | | -0.19*  -0.37-(-0.13) | | -0.06  -0.13-0.08 | | -0.12**  -0.27-(-0.09) | | **Treatment** | | 22.04 | | <0.0001 | |  |
|  |  |  |  |  |  |  |  |  | **Time** | | 11.68 | | 0.002 | |  |
|  |  |  |  |  |  |  |  |  | **Treatment X time** | | 0.19 | | 0.67 | |  |
| Glycine/Serine | 0.35  0.18-0.43 | | 0.35  0.05-0.46 | | 0.22  0.10-0.37 | | 0.21  0.13-0.33 | | **Treatment** | | 0.31 | | 0.58 | |  |
|  |  |  |  |  |  |  |  |  | **Time** | | 9.21 | | 0.005 | |  |
|  |  |  |  |  |  |  |  |  | **Treatment X time** | | 0.21 | | 0.65 | |  |
| Glutamine/  Glutamate | 0.43  -0.02-0.53 | | 0.43  0.23-0.63 | | 0.39  0.33-0.58 | | 0.53  0.37-0.68 | | **Treatment** | | 5.82 | | 0.02 | |  |
|  |  |  |  |  |  |  |  |  | **Time** | | 3.87 | | 0.06 | |  |
|  |  |  |  |  |  |  |  |  | **Treatment X time** | | 0.0004 | | 0.98 | |  |
| Asparagine/  Aspartate | -0.09  -0.30-0.14 | | -0.17  -0.35-0.01 | | -0.02  -0.16-0.28 | | 0.26^####^  -0.08-0.42 | | **Treatment** | | 0.68 | | 0.41 | |  |
|  |  |  |  |  |  |  |  |  | **Time** | | 29.42 | | <0.0001 | |  |
|  |  |  |  |  |  |  |  |  | **Treatment X time** | | 8.11 | | 0.007 | |  |
| Arginine/Citrulline | 0.61  0.05-0.80 | | 0.47  0.12-1.12 | | 0.47  0.23-0.88 | | 1.09***^##^  0.60-1.30 | | **Treatment** | | 10.78 | | 0.002 | |  |
|  |  |  |  |  |  |  |  |  | **Time** | | 5.87 | | 0.02 | |  |
|  |  |  |  |  |  |  |  |  | **Treatment X time** | | 7.79 | | 0.009 | |  |
| ADMA/Arginine | -2.54  -2.90-(-2.19) | | -2.45  -2.81-(-1.92) | | -2.63  -2.92-(-2.13) | | -2.79^#^  -3.11-(-2.51) | | **Treatment** | | 0.28 | | 0.60 | |  |
|  |  |  |  |  |  |  |  |  | **Time** | | 2.94 | | 0.10 | |  |
|  |  |  |  |  |  |  |  |  | **Treatment X time** | | 6.34 | | 0.02 | |  |
| Methionine/  Methionine-SO | 2.16  1.67-2.47 | | 2.29  1.78-2.40 | | 2.17  1.85-2.28 | | 2.25  1.85-2.60 | | **Treatment** | | 0.75 | | 0.40 | |  |
|  |  |  |  |  |  |  |  |  | **Time** | | 0.12 | | 0.73 | |  |
|  |  |  |  |  |  |  |  |  | **Treatment X time** | | 0.26 | | 0.62 | |  |
| Spermidine/  Putrescine | 0.71  0.55-1.16 | | 1.07  0.68-1.31 | | 0.61  0.10-0.83 | | 0.57^###^  0.46-0.75 | | **Treatment** | | 2.88 | | 0.10 | |  |
|  |  |  |  |  |  |  |  |  | **Time** | | 24.54 | | <0.0001 | |  |
|  |  |  |  |  |  |  |  |  | **Treatment X time** | | 4.78 | | 0.04 | |  |
| Serotonin/  Kynurenine | 1.12  0.86-1.39 | | 1.05  0.88-1.42 | | 0.85^@^  0.24-1.13 | | 0.07****^####^  -0.34-0.54 | | **Treatment** | | 31.51 | | <0.0001 | |  |
|  |  |  |  |  |  |  |  |  | **Time** | | 94.52 | | <0.0001 | |  |
|  |  |  |  |  |  |  |  |  | **Treatment X time** | | 28.75 | | <0.0001 | |  |
| Kynurenine/Alpha-Aminoadipic acid | -0.32  -0.50-0.15 | | -0.17  -0.49-(-0.11) | | -0.27  -0.51-0.07 | | 0.09***^##^  -0.13-0.28 | | **Treatment** | | 9.81 | | 0.004 | |  |
|  |  |  |  |  |  |  |  |  | **Time** | | 8.15 | | 0.008 | |  |
|  |  |  |  |  |  |  |  |  | **Treatment X time** | | 7.65 | | 0.01 | |  |
| Leucine/Kynurenine | 2.40  2.02-2.71 | | 2.35  2.16-2.83 | | 2.16  1.91-2.62 | | 2.12^#^  1.77-2.26 | | **Treatment** | | 1.25 | | 0.27 | |  |
|  |  |  |  |  |  |  |  |  | **Time** | | 13.47 | | 0.0008 | |  |
|  |  |  |  |  |  |  |  |  | **Treatment X time** | | 1.38 | | 0.25 | |  |
| Kynurenine/  Tryptophan | -2.10  -2.22-(-1.92) | | -2.06  -2.32-(-1.99) | | -1.97  -2.16-(-1.69) | | -1.65****^####^  -1.73-(-1.41) | | **Treatment** | | 17.01 | | 0.0002 | |  |
|  |  |  |  |  |  |  |  |  | **Time** | | 66.76 | | <0.0001 | |  |
|  |  |  |  |  |  |  |  |  | **Treatment X time** | | 26.24 | | <0.0001 | |  |
| Serotonin/  Tryptophan | -0.97  -1.25-(-0.67) | | -0.98  -1.18-(-0.83) | | -1.14  -1.45-(-0.83) | | -1.62****^####^  -2.01-(-1.11) | | **Treatment** | | 18.15 | | 0.0002 | |  |
|  |  |  |  |  |  |  |  |  | **Time** | | 45.43 | | <0.0001 | |  |
|  |  |  |  |  |  |  |  |  | **Treatment X time** | | 11.11 | | 0.002 | |  |
| lysoPC a C16:1/ lysoPC a C16:0 | -1.85  -1.97-(-1.68) | | -1.76  -1.90-(-1.67) | | -1.80  -2.01-(-1.57) | | -1.73  -1.90-(-1.61) | | **Treatment** | | 2.26 | | 0.14 | |  |
|  |  |  |  |  |  |  |  |  | **Time** | | 1.63 | | 0.21 | |  |
|  |  |  |  |  |  |  |  |  | **Treatment X time** | | 0.46 | | 0.50 | |  |
| lysoPC a C18:2/ lysoPC a C18:1 | 0.42  0.29-0.53 | | 0.38  0.17-0.46 | | 0.48  0.25-0.62 | | 0.35  0.30-0.47 | | **Treatment** | | 6.04 | | 0.02 | |  |
|  |  |  |  |  |  |  |  |  | **Time** | | 0.30 | | 0.59 | |  |
|  |  |  |  |  |  |  |  |  | **Treatment X time** | | 0.25 | | 0.62 | |  |
| lysoPC a C20:4/ lysoPC a C20:3 | 0.71  0.56-0.75 | | 0.75  0.63-0.81 | | 0.57  0.50-0.75 | | 0.78****  0.62-0.93 | | **Treatment** | | 20.85 | | <0.0001 | |  |
|  |  |  |  |  |  |  |  |  | **Time** | | 0.48 | | 0.49 | |  |
|  |  |  |  |  |  |  |  |  | **Treatment X time** | | 7.19 | | 0.01 | |  |

# **Supplementary Table 3**

Main effect of mouse strain on blood plasma levels of metabolites (Bl6 1.5 and 24 h saline group compared to 129Sv 1.5 and 24 saline group, respectively). Statistically significant regression coefficients (ß), confidence intervals (CI) and *t*- and *p* values (derived from General linear model GLM analysis) of log_10_-transformed metabolite levels.

| Bl6 vs 129Sv 1.5 h saline | | | | |
| --- | --- | --- | --- | --- |
| Metabolites | **Beta (ß)** | **ß (95 % Cl)** | ***t*-value** | ***p*-value** |
| Acylcarnitines | | | | |
| Isovalerylcarnitine and 2-methybutyrylcarnitine (C5-) | 0.88 | 0.64, 1.11 | 7.73 | <0.0001 |
| Biogenic Amines | | | | |
| Acetylornithine (Ac-Orn) | -0.90 | -1.12, -0.69 | -8.98 | <0.0001 |
| Alpha-aminoadipic-acid (Alpha-AAA) | -0.91 | -1.12, -0.71 | -9.37 | <0.0001 |
| Carnosine | -0.89 | -1.12, -0.66 | -8.14 | <0.0001 |
| Glycerophospholipids | | | | |
| *Lysophosphatidylcholine acyls* | | | | |
| lysoPC a C16:1 | -0.75 | -1.08, -0.42 | -4.80 | 0.0001 |
| lysoPC a C20:3 | -0,57 | -0,98, -0,16 | -2.92 | 0.009 |
| *Phosphatidylcholine diacyls* | | | | |
| PC aa C34:3 | -0.66 | -1.03, -0.29 | -3.75 | 0.001 |
| *Phosphatidylcholine acyl-alkyls* | | | | |
| PC ae C38:4 | 0.61 | 0.21, 1.00 | 3.23 | 0.0046 |
| Sphingolipids | | | | |
| SM (OH) C22:2 | 0.80 | 0.51, 1.10 | 5.75 | <0.0001 |
| Monosaccharides | | | | |
| Sum of hexoses (H1) | -0.60 | -1.00, -0.21 | -3.21 | 0.0049 |
| Bl6 vs 129Sv 24 h saline | | | | |
| Acylcarnitines | | | | |
| Isovalerylcarnitine and 2-methybutyrylcarnitine (C5-) | 0.87 | 0.58, 1.17 | 6.49 | <0.0001 |
| Biogenic Amines | | | | |
| Acetylornithine (Ac-Orn) | -0.94 | -1.14, -0.74 | -10.19 | <0.0001 |
| Alpha-aminoadipic-acid (Alpha-AAA) | -0.93 | -1.15, -0.71 | -9.25 | <0.0001 |
| Carnosine | -0.80 | -1.16, -0.44 | -4.77 | 0.00036 |
| Glycerophospholipids | | | | |
| *Lysophosphatidylcholine acyls* | | | | |
| lysoPC a C16:1 | -0.76 | -1.15, -0.38 | -4.26 | 0.0009 |
| *Phosphatidylcholine acyl-alkyls* | | | | |
| PC ae C36:2 | 0.70 | 0.27, 1.13 | 3.51 | 0.0038 |
| PC ae C38:2 | 0.65 | 0.20, 1.11 | 3.10 | 0.008 |
| PC ae C40:6 | 0.80 | 0.45, 1.16 | 4.88 | 0.0003 |
| Sphingolipids | | | | |
| SM (OH) C14:1 | 0.69 | 0.26, 1.13 | 3.47 | 0.004 |
| SM (OH) C22:2 | 0.82 | 0.47, 1.16 | 5.11 | 0.0002 |

# **Supplementary Table 4**

Main effect of the LPS administration on blood plasma levels of metabolites and body weight change (Δ body weight) in Bl6 and 129Sv 1.5 and 24 h after LPS administration. Statistically significant regression coefficients (ß), confidence intervals (CI) and *t*- and *p* values (derived from General linear model GLM analysis) of log_10_-transformed metabolite levels.

| Bl6 1.5 h LPS | | | | |
| --- | --- | --- | --- | --- |
| Metabolites | **Beta (ß)** | **ß (95 % Cl)** | ***t*-value** | ***p*-value** |
| Acylcarnitines | | | | |
| Propionylcarnitine (C3) | -0.84 | -1.15, -0.52 | -5.68 | <0.0001 |
| Amino Acids | | | | |
| Alanine (Ala) | -0.81 | -1.15, -0.47 | -5.16 | 0.0001 |
| Glycine (Gly) | -0.74 | -1.12, -0.35 | -4.08 | 0.001 |
| Histidine (His) | -0.78 | -1.14, -0.41 | -4.59 | 0.0004 |
| Phenylalanine (Phe) | -0.73 | -1.12, -0.33 | -3.96 | 0.001 |
| Proline (Pro) | -0.86 | -1.15, -0.56 | -6.20 | <0.0001 |
| Serine (Ser) | -0.86 | -1.15, -0.56 | -6.20 | <0.0001 |
| Tyrosine (Tyr) | -0.72 | -1.12, -0.32 | -3.92 | 0.002 |
| Biogenic Amines | | | | |
| Acetylornithine (Ac-Orn) | -0.73 | -1.12, -0.34 | -4.01 | 0.001 |
| Bl6 24 h LPS | | | | |
| Metabolites | **Beta (ß)** | **ß (95 % Cl)** | ***t*-value** | ***p*-value** |
| 24 h weight change | | | | |
| Δ body weight | -0.90 | -1.12, -0.68 | -8.64 | <0.0001 |
| Amino Acids | | | | |
| Citrulline (Cit) | -0.83 | -1.11, -0.54 | -6.05 | <0.0001 |
| Biogenic Amines | | | | |
| Acetylornithine (Ac-Orn) | -0.86 | -1.12, -0.60 | -6.90 | <0.0001 |
| Kynurenine | 0.87 | 0.62, 1.12 | 7.23 | <0.0001 |
| Putrescine | 0.88 | 0.64, 1.12 | 7.80 | <0.0001 |
| Glycerophospholipids | | | | |
| *Lysophosphatidylcholine acyls* | | | | |
| lysoPC a C16:1 | -0.88 | -1.12, -0.64 | -7.64 | <0.0001 |
| lysoPC a C20:3 | -0.88 | -1.12, -0.64 | -7.61 | <0.0001 |
| lysoPC a C20:4 | -0.81 | -1.11, -0.52 | -5.78 | <0.0001 |
| *Phosphatidylcholine diacyls* | | | | |
| PC aa C36:1 | 0.85 | 0.57, 1.12 | 6.56 | <0.0001 |
| PC aa C40:6 | 0.85 | 0.58, 1.12 | 6.68 | <0.0001 |
| *Phosphatidylcholine acyl-alkyls* | | | | |
| PC ae C38:2 | -0.85 | -1.12, -0.59 | -6.74 | <0.0001 |
| Sphingolipids | | | | |
| SM (OH) C22:1 | 0.92 | 0.72, 1.12 | 9.58 | <0.0001 |
| SM (OH) C22:2 | 0.89 | 0.66, 1.12 | 8.05 | <0.0001 |
| SM C16:0 | 0.94 | 0.76, 1.12 | 11.02 | <0.0001 |
| SM C24:0 | 0.85 | 0.57, 1.12 | 6.53 | <0.0001 |
| SM C24:1 | 0.95 | 0.80, 1.11 | 12.84 | <0.0001 |
| Monosaccharides | | | | |
| Sum of Hexoses (H1) | -0.78 | -1.10, -0.45 | -5.06 | <0.0001 |
| 129Sv 1.5 h LPS | | | | |
| Metabolites | **Beta (ß)** | **ß (95 % Cl)** | ***t*-value** | ***p*-value** |
| Acylcarnitines | | | | |
| Acetylcarnitine (C2) | -0.84 | -1.14, -0.54 | -5.93 | <0.0001 |
| Propionylcarnitine (C3) | -0.87 | -1.14, -0.60 | -6.81 | <0.0001 |
| Butyryl- and isobutyrylcarnitine (C4-) | -0.85 | -1.14, -0.56 | -6.22 | <0.0001 |
| Isovalerylcarnitine and 2-methybutyrylcarnitine (C5-) | -0.78 | -1.12, -0.43 | -4.78 | 0.0002 |
| Amino Acids | | | | |
| Citrulline (Cit) | -0.58 | -1.03, -0.13 | -2.76 | 0.01 |
| Glycerophospholipids | | | | |
| *Phosphatidylcholine diacyls* | | | | |
| PC aa C42:0 | 0.64 | 0.22, 1.06 | 3.22 | 0.006 |
| Sphingolipids | | | | |
| SM (OH) C16:1 | 0.56 | 0.11, 1.02 | 2.64 | 0.02 |
| 129Sv 24 h LPS | | | | |
| Metabolites | **Beta (ß)** | **ß (95 % Cl)** | ***t*-value** | ***p*-value** |
| 24 h body weight change | | | | |
| Δ body weight | -0.93 | -1.12, -0.74 | -10.19 | <0.0001 |
| Acylcarnitines | | | | |
| Tetradecenoylcarnitine (C14:1) | 0.76 | 0.42, 1.11 | 4.73 | 0.0002 |
| Hexadecanoylcarnitine (C16) | 0.77 | 0.43, 1.11 | 4.83 | 0.0002 |
| Hydroxyhexadecanoylcarnitine  (C16-OH) | 0.76 | 0.42, 1.10 | 4.70 | 0.0002 |
| Octadecenoylcarnitine (C18:1) | 0.78 | 0.45, 1.11 | 5.04 | 0.0001 |
| Amino Acids | | | | |
| Citrulline (Cit) | -0.89 | -1.13, -0.65 | -7.77 | <0.0001 |
| Biogenic Amines | | | | |
| Kynurenine | 0.87 | 0.61, 1.13 | 7.01 | <0.0001 |
| Glycerophospholipids | | | | |
| *Lysophosphatidylcholine acyls* | | | | |
| lysoPC a C20:3 | -0.85 | -1.13, -0.56 | -6.34 | <0.0001 |
| *Phosphatidylcholine diacyls* | | | | |
| PC aa C36:1 | 0.93 | 0.74, 1.12 | 10.19 | <0.0001 |
| PC aa C38:6 | 0.85 | 0.58, 1.13 | 6.52 | <0.0001 |
| PC aa C40:6 | 0.95 | 0.80, 1.11 | 12.82 | <0.0001 |
| PC aa C42:5 | 0.80 | 0.48, 1.12 | 5.36 | <0.0001 |
| Sphingolipids | | | | |
| SM (OH) C14:1 | 0.88 | 0.64, 1.13 | 7.55 | <0.0001 |
| SM (OH) C16:1 | 0.79 | 0.47, 1.12 | 5.23 | <0.0001 |
| SM (OH) C22:1 | 0.95 | 0.79, 1.11 | 12.55 | <0.0001 |
| SM (OH) C22:2 | 0.95 | 0.80, 1.11 | 12.40 | <0.0001 |
| SM (OH) C24:1 | 0.92 | 0.72, 1.13 | 9.73 | <0.0001 |
| SM C16:0 | 0.97 | 0.84, 1.10 | 15.98 | <0.0001 |
| SM C16:1 | 0.94 | 0.75, 1.12 | 10.60 | <0.0001 |
| SM C18:0 | 0.91 | 0.70, 1.13 | 8.91 | <0.0001 |
| SM C18:1 | 0.92 | 0.71, 1.13 | 9.39 | <0.0001 |
| SM C24:0 | 0.93 | 0.72, 1.13 | 9.74 | <0.0001 |
| SM C24:1 | 0.98 | 0.88, 1.08 | 20.55 | <0.0001 |

**Supplementary Figure 1**. Differences in basal levels of **(A)** Acetyl-ornithine (Ac-Orn), **(B)** alpha-Aminoadipic acid (alpha-AAA), **(C)** carnosine, **(D)** short-chain acylcarnitine, C4-, **(E)** short-chain acylcarnitine, C5- and **(F)** sphingolipid, SM(OH) C22:2 between Bl6 and 129Sv saline administration groups. Data of metabolite concentrations of 1.5 h and 24 h control groups are pooled together and expressed as mean ± SD. Data was analyzed using *t*-test and Bonferroni correction (statistically significant *p* value less or equal to 0.0002).
